# Physiologically Based Pharmacokinetic Modeling of Rosuvastatin to Predict Transporter-Mediated Drug-Drug Interactions

**DOI:** 10.1007/s11095-021-03109-6

**Published:** 2021-10-18

**Authors:** Nina Hanke, José David Gómez-Mantilla, Naoki Ishiguro, Peter Stopfer, Valerie Nock

**Affiliations:** 1 Translational Medicine & Clinical Pharmacology, Boehringer Ingelheim Pharma GmbH & Co. KG, Birkendorfer Str. 65, 88397 Biberach, Germany; 2 Kobe Pharma Research Institute, Nippon Boehringer Ingelheim Co. Ltd, Kobe, Japan

**Keywords:** Physiologically based pharmacokinetic modeling (PBPK), Rosuvastatin, Drug-drug interactions (DDIs), Organic anion transporting polypeptide 1B1/1B3 (OATP1B1/1B3), Model-informed drug discovery and development (MID3)

## Abstract

**Purpose:**

To build a physiologically based pharmacokinetic (PBPK) model of the clinical OATP1B1/OATP1B3/BCRP victim drug rosuvastatin for the investigation and prediction of its transporter-mediated drug-drug interactions (DDIs).

**Methods:**

The Rosuvastatin model was developed using the open-source PBPK software PK-Sim®, following a middle-out approach. 42 clinical studies (dosing range 0.002–80.0 mg), providing rosuvastatin plasma, urine and feces data, positron emission tomography (PET) measurements of tissue concentrations and 7 different rosuvastatin DDI studies with rifampicin, gemfibrozil and probenecid as the perpetrator drugs, were included to build and qualify the model.

**Results:**

The carefully developed and thoroughly evaluated model adequately describes the analyzed clinical data, including blood, liver, feces and urine measurements. The processes implemented to describe the rosuvastatin pharmacokinetics and DDIs are active uptake by OATP2B1, OATP1B1/OATP1B3 and OAT3, active efflux by BCRP and Pgp, metabolism by CYP2C9 and passive glomerular filtration. The available clinical rifampicin, gemfibrozil and probenecid DDI studies were modeled using in vitro inhibition constants without adjustments. The good prediction of DDIs was demonstrated by simulated rosuvastatin plasma profiles, DDI AUC_last_ ratios (AUC_last_ during DDI/AUC_last_ without co-administration) and DDI C_max_ ratios (C_max_ during DDI/C_max_ without co-administration), with all simulated DDI ratios within 1.6-fold of the observed values.

**Conclusions:**

A whole-body PBPK model of rosuvastatin was built and qualified for the prediction of rosuvastatin pharmacokinetics and transporter-mediated DDIs. The model is freely available in the Open Systems Pharmacology model repository, to support future investigations of rosuvastatin pharmacokinetics, rosuvastatin therapy and DDI studies during model-informed drug discovery and development (MID3).

**Supplementary Information:**

The online version contains supplementary material available at 10.1007/s11095-021-03109-6.

## Introduction

The most commonly recommended clinical substrates for the study of organic anion transporting polypeptide 1B1 and 1B3 (OATP1B1/OATP1B3) mediated drug-drug interactions (DDIs) are rosuvastatin, pravastatin and pitavastatin [1–3]. However, these drugs are not only substrates of OATP1B1 and OATP1B3, but also of OATP2B1, breast cancer resistance protein (BCRP) and P-glycoprotein (Pgp) [1, 3]. Additionally, unspecific inhibitors (or inducers) that affect multiple transporters are routinely used, which complicates the interpretation of clinical DDI study results. Given these challenges, physiologically based pharmacokinetic (PBPK) modeling is a valuable tool to help with the design of transporter DDI studies and to delineate and understand their findings.

Rosuvastatin is a competitive inhibitor of the liver 3-hydroxy-3-methyl-glutaryl-coenzyme A reductase (HMG-CoA reductase), and it is prescribed to treat hyperlipidemia, to slow the progression of atherosclerosis and to prevent cardiovascular events [4]. Inhibition of HMG-CoA reductase decreases the production of cholesterol in the liver, which stimulates hepatocellular uptake of low-density lipoprotein (LDL). Rosuvastatin has also been shown to increase high-density lipoprotein (HDL) and to lower triglyceride concentrations in the blood [5].

**Fig. 1:**
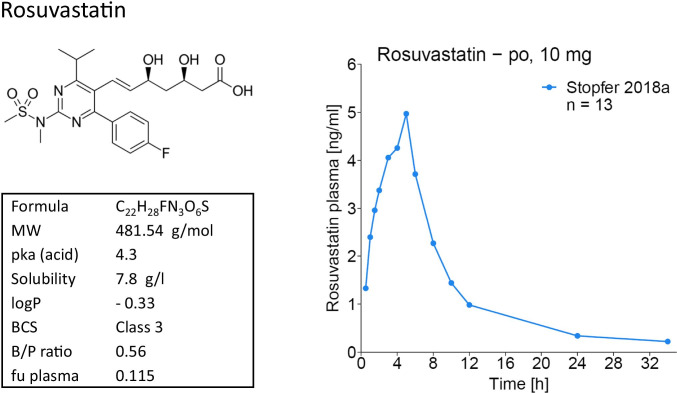
Rosuvastatin skeletal formula, properties and a typical plasma concentration–time profile plotted with data from [61]

Rosuvastatin is a carboxylic acid, administered as its calcium salt. It is a hydrophilic agent with low lipophilicity (BCS class 3) and poor passive permeation of biological membranes (blood/plasma ratio 0.56 [6–8]). The structural formula, physicochemical properties and a typical plasma concentration–time profile of rosuvastatin are shown in Fig. 1. Absorption is estimated at 25% of an administered dose with an absolute bioavailability of 20% [9, 10]. Rosuvastatin is mainly distributed into the liver, via active transport by OATP1B1 and, to a lesser extent, by OATP1B3 [11, 12]. In vitro studies have shown that rosuvastatin undergoes extremely slow metabolism (0% over 3 h in human liver microsomes, 5%–50% over 3 days in human hepatocytes), with CYP2C9 as the primary enzyme involved [13]. In a clinical study, co-administration of the potent CYP2C9 inhibitor fluconazole increased rosuvastatin AUC_last_ and C_max_ by 14% and 9%, respectively [14]. Following oral administration of radiolabeled rosuvastatin, 90% of the dose were recovered in the feces and 10% in the urine, both mostly as parent compound [15]. Maximum rosuvastatin plasma concentrations are reached approximately 5 h after oral administration. Following intravenous administration of rosuvastatin, 30% of the dose were excreted unchanged in the urine, with a renal clearance of 227 mL/min [10]. This relatively high renal clearance (fraction unbound in plasma is 11.5% [16]) suggests active tubular secretion. The terminal elimination half-life of rosuvastatin is approximately 19 h, and patients are typically treated once daily with a maximum dose of 40 mg (only for patients not reaching their LDL-cholesterol goal with 20 mg [4]). Rosuvastatin exposure is dose proportional in the range of 10–80 mg [17].

Compared to other statins, rosuvastatin shows the highest LDL-lowering efficacy, with minimal uptake into non-hepatic tissues, limiting adverse effects [18]. In addition, it has low potential for drug-drug interactions via CYP enzymes, but as it is actively transported by several different drug transporters, there are DDIs described with inhibitors of OATP1B1/1B3 (rifampicin single dose), BCRP and OATP1B1/1B3 (cyclosporine), OAT3 and OATP1B1/1B3 (gemfibrozil, probenecid), or Pgp and OATP1B1/1B3 (ritonavir) [19, 20].

The objective of this study was to develop a whole-body PBPK model of rosuvastatin to support the investigation of drug-drug interactions, that (i) reliably predicts the rosuvastatin concentrations in blood, liver, feces and urine, (ii) incorporates the most important transporters involved in rosuvastatin pharmacokinetics and drug-drug interactions and (iii) mechanistically describes the impact of the perpetrator drugs rifampicin, gemfibrozil and probenecid on the pharmacokinetics of rosuvastatin. The thoroughly evaluated model is freely available in the Open Systems Pharmacology PBPK model repository (https://www.open-systems-pharmacology.org), and the Electronic Supplementary Material (ESM) to this paper was compiled to serve as comprehensive reference manual with full documentation of the model evaluation.

## Materials and Methods

### PBPK Model Building

PBPK model building was started with an extensive literature search to collect physicochemical parameters, information on drug transporters and metabolizing enzymes involved in the pharmacokinetics of rosuvastatin as well as data of clinical rosuvastatin studies (intravenous and oral administration, single- and multiple dose regimens, urine, feces and tissue data).

To curate the dataset for rosuvastatin model development, published plasma concentration–time profiles were digitized [21], evaluated and divided into a training dataset for model building (approx. 1/3) and a test dataset for model evaluation (approx. 2/3). The clinical studies for the training dataset were selected from rosuvastatin plasma concentration–time profile dose-normalized plots, to include representative studies of intravenous, oral and multiple-dose administration over a broad dosing range, as well as information on rosuvastatin in urine, feces and tissue. A table listing all utilized clinical studies with details on the administration regimens, study demographics and references is provided in the ESM (Table S3.2.1).

Model input parameters that could not be informed from experimental reports were optimized by fitting the model simultaneously to the observed data of all studies assigned to the training dataset, using the Levenberg–Marquardt algorithm with multiple random starting values, as well as starting values from literature to avoid trapping in local minima. To limit the parameters to be optimized during model building, the minimal number of processes necessary to mechanistically describe the pharmacokinetics and DDIs of rosuvastatin were implemented into the model. If two transporters show very similar expression patterns and affinity, optimizing the transport rate constants of both transporters would lead to identifiability issues. Therefore, only the transporter with the higher affinity for the respective substrate was implemented, to describe a transport that probably is accomplished by both transporters in vivo.

### Virtual Individuals

The rosuvastatin model was based on data from healthy volunteers. For each simulated study, a virtual mean individual was generated according to the ethnicity, sex, age, weight and height documented in the respective study report. If demographic information was missing, the following default values were substituted: European, male, 30 years of age, 73 kg body weight and 176 cm body height (characteristics from the PK-Sim® population database [22, 23]). Drug transporters and metabolizing enzymes were implemented in accordance with the literature, using the PK-Sim® expression database to define their relative expression in the different organs of the body [24].

### PopPK Model Building and Evaluation

The typical rosuvastatin plasma concentration–time profile shows an unusual shape with a slow absorption phase and late C_max_ (t_max_ = 5.0 h). This delayed absorption has been described previously [25], but a mechanistic explanation was not provided in the literature. Therefore, a population pharmacokinetic (PopPK) analysis was performed to better understand the rosuvastatin absorption phase and use that as an input for the PBPK model.

PopPK analysis was performed using nonlinear mixed-effects modeling techniques in NONMEM 7.4.3. Model selection was based on the objective function value (OFV), goodness-of-fit plots and the precision of the parameter estimates. A nested model was considered superior to another, if the OFV was reduced by 3.84 units or more (χ^2^ test statistic, p < 0.05, 1 degree of freedom). The First-Order Conditional Estimation with Interaction (FOCE-I) method was applied, and models were coded in the ADVAN6 subroutine. Model building was performed using individual rosuvastatin plasma concentration–time profiles from two representative studies of oral rosuvastatin administration [26, 27], complemented with the digitized mean data of the only available intravenous study [10]. One-, two- and three-compartment models were tested, with first-order and saturable elimination (Michaelis–Menten) kinetics. Afterwards, different absorption models, such as zero-order, first-order and mixed parallel zero- and first-order absorption processes as well as split doses and saturable absorption rates were evaluated. Based on the structural base model, interindividual variabilities (IIVs) were modeled exponentially and evaluated univariately. IIVs were added to the model if they improved the model in a statistically significant manner and if the parameter estimates of the model remained stable.

After a solid rosuvastatin PopPK model was established, rosuvastatin data from rifampicin-rosuvastatin, gemfibrozil-rosuvastatin and probenecid-rosuvastatin DDI studies were added to the dataset, and the model was applied to investigate the differences in rosuvastatin absorption, bioavailability and clearance during these DDIs, using covariate factors on the respective model parameters, to describe the effects of the different DDIs. As observed data, individual rosuvastatin plasma concentration–time profiles before and during co-administration of rifampicin or probenecid [28], as well as digitized geometric mean data of the only published gemfibrozil-rosuvastatin DDI study [29] were added to the dataset.

The results of the PopPK analysis regarding the rosuvastatin absorption phase (please see PopPK modeling results section) were integrated into the administration protocols of all oral rosuvastatin PBPK simulations in PK-Sim®, which greatly improved the results of the PBPK parameter identification.

### PBPK Model Evaluation

Model performance was evaluated using various approaches. The predicted plasma concentrations were compared to the observed clinical data in plasma concentration–time plots and goodness-of-fit plots. In addition, the model performance was evaluated by comparison of predicted to observed area under the plasma concentration–time curve from the time of drug administration to the time of the last measured concentration (AUC_last_) and peak plasma concentration (C_max_) values. As quantitative measures of the model performance, the mean relative deviation (MRD) of all predicted plasma concentrations (Eq. 1) and the geometric mean fold error (GMFE) of all predicted AUC_last_ and C_max_ values (Eq. 2) were calculated. MRD and GMFE values ≤ 2 were interpreted as signs of adequate model performance.

1 $$\begin{array}{cc}\mathrm{MRD}=10^{\mathrm x};&\mathrm x=\sqrt{\frac1{\mathrm k}{\textstyle\sum_{\mathrm i=1}^{\mathrm k}}\left(\log_{10}\;{\mathrm c}_{\mathrm{predicted},\;\mathrm i}-\log_{10}\;{\mathrm c}_{\mathrm{observed},\;\mathrm i}\right)^2}\end{array}$$

where c_predicted, i_ = predicted plasma concentration, c_observed, i_ = corresponding observed plasma concentration, k = number of observed values.

2 $$\begin{array}{cc}\mathrm{GMFE}=10^{\mathrm x};&\mathrm x=\frac1{\mathrm m}\end{array}{\textstyle\sum_{\mathrm i=1}^{\mathrm m}}\left|\log_{10}\left(\frac{\mathrm{predicted}\;\mathrm{PK}{\;\mathrm{parameter}}_{\mathrm i}}{\mathrm{observed}\;\mathrm{PK}{\;\mathrm{parameter}}_{\mathrm i}}\right)\right|$$

where predicted PK parameter_i_ = predicted AUC_last_ or C_max_ value, observed PK parameter_i_ = corresponding observed AUC_last_ or C_max_ value, m = number of studies.

Furthermore, sensitivity analysis results were assessed. A detailed description of the sensitivity calculation and settings is provided in Sect. 1.4 of the ESM and in the Open Systems Pharmacology Suite manual [30].

### PBPK DDI Modeling

Further information to develop the rosuvastatin model was gathered from clinical DDI studies. A good description of the rosuvastatin pharmacokinetics during co-treatment with inhibitors of rosuvastatin transport increases the confidence in the model implementation of these transport processes. Therefore, the DDIs of rifampicin, gemfibrozil and probenecid with rosuvastatin were modeled using previously developed and qualified perpetrator models [31–36]. Interaction parameters were collected from in vitro literature and in-house measurements of drug transporter and metabolic enzyme inhibition. The mathematical implementation of competitive inhibition in PK-Sim is shown in Sect. 1.5 of the ESM and in the Open Systems Pharmacology Suite manual [30].

This model required the implementation of a relatively high number of different transporters and enzymes, which increases the risk of identifiability problems during the parameter optimizations. Therefore, contrary to our usual approach to predict the clinical DDI data as a means of model evaluation, data of all three analyzed DDIs were included into the training dataset, to inform and distinguish the fractions transported and metabolized by the five different transporters and one CYP enzyme implemented in the final rosuvastatin model. Therefore, the observed data of four of the seven available clinical DDI studies were included for model building (rosuvastatin with intravenous and oral rifampicin [28, 37], gemfibrozil [29] and probenecid [28]), and the data of three studies were used for model evaluation (rosuvastatin with intravenous and oral rifampicin [1, 38]). Tables listing all utilized clinical DDI studies with details on the administration regimens, study demographics and references are provided in the ESM (Tables S4.3.1, S5.3.1 and S6.3.1).

### PBPK DDI Performance Evaluation

All DDI predictions were evaluated by comparison of the predicted victim drug plasma concentration–time profiles with the clinically observed ones, during monotherapy and perpetrator co-administration. In addition, the predicted DDI AUC_last_ ratios (Eq. 3) and DDI C_max_ ratios (Eq. 4) were assessed, applying the DDI prediction success criteria proposed by Guest et al., who suggested new prediction success limits that keep the two-fold criterion for the prediction of strong DDIs, but have continuously stricter requirements for the prediction of moderate (5 > AUC ratio ≥ 2) and weak (2 > AUC ratio ≥ 1.25) DDIs. These new limits avoid bias towards successful prediction at lower interaction levels but still allow for the normal variability observed between clinical studies within the bioequivalence limits (0.8- to 1.25-fold) [39].

3 $$\mathrm{DDI}\;{\mathrm{AUC}}_{\mathrm{last}}\;\mathrm{ratio}\;=\;\frac{{\mathrm{AUC}}_{\mathrm{last}}\;\mathrm{victim}\;\mathrm{drug}\;\mathrm{during}\;\mathrm{co}-\mathrm{administration}}{{\mathrm{AUC}}_{\mathrm{last}}\;\mathrm{victim}\;\mathrm{drug}\;\mathrm{control}}$$

4 $$\text{DDI C}_{\text{max}}\, {\text{ratio}} = \frac{{\text{C}}_{{\text{max}}}\,{\text{victim drug during co-administration}}}{{\text{C}}_{{\text{max}}}\,{\text{victim drug control}}}$$

In addition, GMFEs of the predicted DDI AUC_last_ ratios and DDI C_max_ ratios were calculated according to Eq. 2, and GMFE values ≤ 2 were interpreted as a sign of adequate model performance.

### Software

PBPK modeling was accomplished using the free and open-source modeling software PK-Sim® (Open Systems Pharmacology Suite 9.1, www.open-systems-pharmacology.org [40, 41]). Published plasma concentration–time profiles from the literature were digitized with GetData Graph Digitizer (version 2.26.0.20, © S. Fedorov). Population pharmacokinetic analysis was accomplished using nonlinear mixed-effects modeling techniques implemented in NONMEM® (version 7.4.3). Generation of graphics and calculation of model performance measures were performed with R (version 4.0.2, The R Foundation for Statistical Computing) in RStudio (version 1.3.1093, RStudio, Inc., Boston, MA, USA).

## Results

### PBPK Model Building and Evaluation

The rosuvastatin PBPK model was developed using data of 42 clinical studies (10 in the training, 32 in the test dataset), including intravenous and oral administration, microdosing, multiple-dose regimens, urine and feces measurements, and positron emission tomography (PET) data. In addition, data of 7 clinical DDI studies was included for model building and evaluation (4 in the training, 3 in the test dataset). A complete list of the utilized studies of rosuvastatin monotherapy, covering a dose range of 0.002–80.0 mg rosuvastatin, with details on study demographics and assignment to training or test dataset, is provided in Table S3.2.1. Lists of the employed DDI studies with rifampicin, gemfibrozil and probenecid are provided in Tables S4.3.1, S5.3.1 and S6.3.1. Studies in Asian individuals were excluded, as Asians show significantly higher rosuvastatin exposure than Caucasians, but this difference cannot be fully explained, yet [42, 43].

**Fig. 2:**
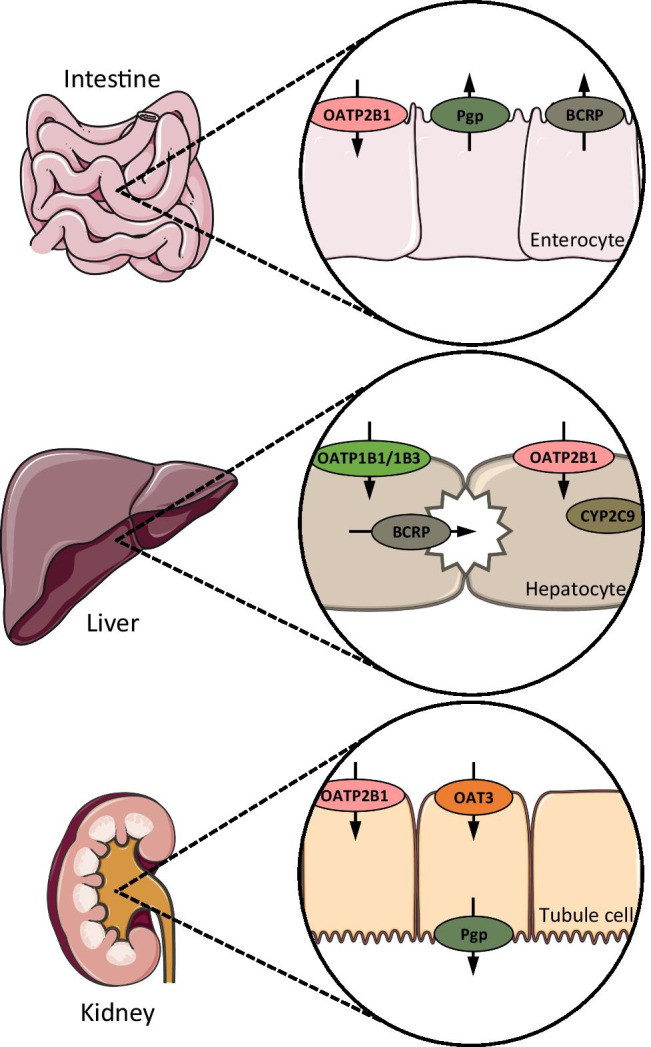
Rosuvastatin transport and metabolism. Main sites of action of the transporters and enzymes implemented to model the absorption, distribution, metabolism and excretion of rosuvastatin. Processes implemented in the model but with less than 20% relative expression in the depicted organs were excluded from this illustration (namely CYP2C9 in the intestine, Pgp in the liver and BCRP in the kidney). Drawings by Servier, licensed under CC BY 3.0

To describe the pharmacokinetics of rosuvastatin, active transport by OATP2B1, OATP1B1/1B3, OAT3, Pgp and BCRP as well as metabolism by CYP2C9 were implemented into the model. These drug transporters/enzymes were incorporated into the different modeled organs and tissues in agreement with the current literature, with their main sites of action in the model illustrated in Fig. 2. OATP1B1 was implemented as a substitute for the combined rosuvastatin transport by OATP1B1/1B3, to avoid identifiability problems fitting both transport rate constants and as OATP1B1 was reported to be more relevant for rosuvastatin liver uptake with a contribution of 77–88% [11, 12]. In addition, very similar inhibition constants of rifampicin for these two members of the OATP family were published most recently (OATP1B1 Ki = 0.63 µM and OATP1B3 Ki = 0.69 µM [44], OATP1B1 Ki = 0.29 µM and OATP1B3 Ki = 0.50 µM [45]), that could not be utilized to differentiate the impact of these two OATP family members. In addition to the implemented active processes, the model calculates passive glomerular filtration and enterohepatic circulation of the rosuvastatin that is effluxed into the bile. The drug-dependent model parameters are summarized in Table S3.3.1. The system-dependent parameters of the implemented transporters and enzymes are listed in Table S7.0.1.

**Fig. 3:**
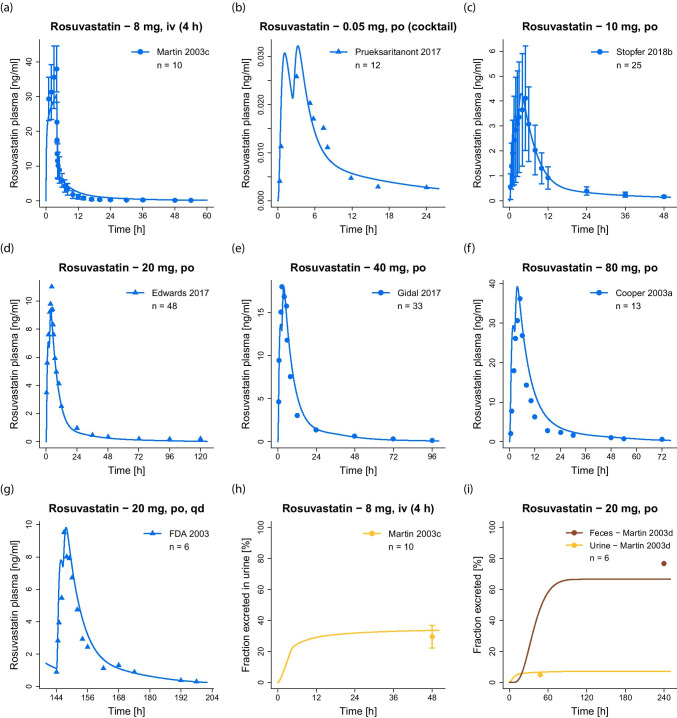
Rosuvastatin model predictions. (**a–g**) Selected simulations of rosuvastatin plasma concentration–time profiles, compared to observed data [9, 10, 27, 38, 62–64]. (**h–i**) Selected simulations of rosuvastatin fractions excreted unchanged in urine and feces, compared to observed data [10, 65]. Simulations are shown as lines, clinical data are shown as dots (training dataset) or triangles (test dataset) ± standard deviation, if available. Details on study populations are summarized in Table S3.2.1. Simulations of all 42 clinical studies used for model development, compared to observed data, are documented in Fig. S3.4.1 (plasma, semilogarithmic plots), Fig. S3.4.2 (plasma, linear plots), Figs. S3.4.3 and S3.4.4 (PET data) and Fig. S3.4.5 (fractions excreted)

**Fig. 4:**
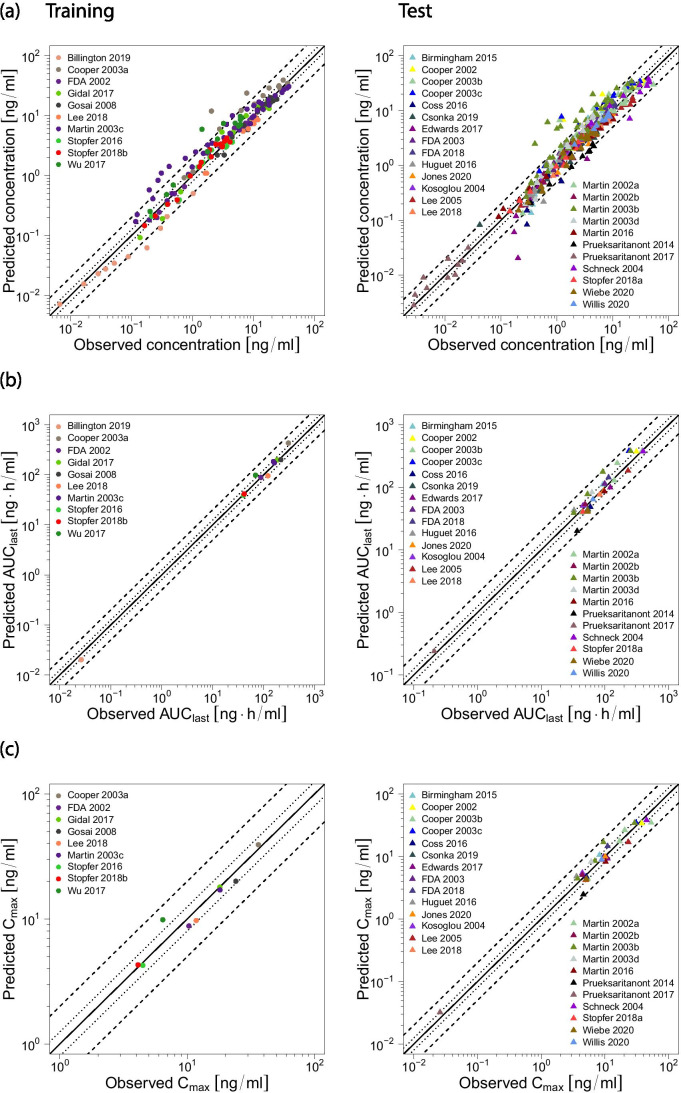
Rosuvastatin model performance. Predicted compared to observed rosuvastatin (**a**) plasma concentrations, (**b**) AUC_last_ and (**c**) C_max_ values of all analyzed clinical studies, separated by training (left column, dots) and test dataset (right column, triangles). The solid line marks the line of identity. Dotted lines indicate 1.25-fold, dashed lines indicate twofold deviation. The individual AUC_last_ and C_max_ values, mean GMFE values and ranges are listed in Table S3.5.2

The good model performance is illustrated in Fig. 3, showing predicted compared to observed plasma concentration–time profiles of selected studies covering different administration protocols, as well as fractions excreted in urine and feces. Model simulations of all 42 clinical studies, superimposed with their respective observed data, are documented in the ESM (both semilogarithmic and linear plots). Goodness-of-fit plots showing the predicted versus observed plasma concentrations of all studies are presented in Fig. 4a. The MRD value over all studies was calculated at 1.48 (range 1.08–2.17), MRD values of all 42 analyzed studies are listed in Table S3.5.1.

Predicted compared to observed rosuvastatin AUC_last_ and C_max_ values are shown in Fig. 4b-c, with 42/42 of the predicted AUC_last_ and 40/40 of the predicted C_max_ values within twofold of the observed data. The GMFE values over all studies were calculated at 1.24 (range 1.00–1.89) for the predicted AUC_last_ values and at 1.22 (range 1.00–1.86) for the predicted C_max_ values, further demonstrating the good model performance. AUC_last_, C_max_ and corresponding GMFE values of all 42 analyzed studies are listed in Table S3.5.2.

The sensitivity analysis of the final rosuvastatin model showed that the predictions are sensitive to the values of rosuvastatin fraction unbound in plasma, lipophilicity, OATP1B1/1B3 Km and Pgp Km (all literature values), as well as to the values of intestinal permeability, OATP1B1/1B3 kcat and Pgp kcat (all optimized), confirming that the most impactful transport in the model is OATP1B1/1B3, followed by Pgp (Fig. S3.5.3).

### PopPK Modeling of Rosuvastatin

Rosuvastatin PBPK model building was supported by a PopPK analysis to investigate and improve the description of the slow absorption phase and very late C_max_ (t_max_ = 5.0 h) observed in the rosuvastatin plasma concentration–time profiles (see Fig. 1).

The pharmacokinetics of rosuvastatin were best described by a two-compartment model with first-order elimination from the central compartment. The absorption phase was best described using a split dose approach where the fraction of the second dose was estimated, and the fraction of the first dose was calculated (1 – fraction second dose). Both doses were absorbed with the same absorption rate constant, and the absorption of the second dose was delayed by a lag time. A schematic representation of the PopPK model is shown in Fig. S2.4.1.

The parameter estimates of the final model are presented in Table S2.4.1. All parameters were estimated precisely with relative standard errors < 25%. Interindividual variability was incorporated on the fraction of dose attributed to the second dose, the total bioavailability and the clearance. The final rosuvastatin PopPK model was then applied to investigate the rosuvastatin absorption phase during the different DDIs. Adding the data of the DDI studies to the PopPK dataset and using covariate factors on the bioavailability and clearance, the effects of the different DDIs could be well described and the parameter estimates between the model without and with DDI data were very similar (Table S2.4.1). Diagnostic goodness-of-fit plots (Fig. S2.4.2) and plots of predicted compared to observed concentration–time profiles (Figs. S2.4.3 to S2.4.6) demonstrate the good descriptive performance of the model. The NONMEM code of the final model is provided in Sect. 2.4.2 of the ESM.

The final PopPK model adequately captures the slow absorption phase and shape of all individual profiles. A population median of 63.4% of the total dose was estimated for the first dose, absorbed immediately without a lag time. The remaining 36.6% of the total dose were assigned to the second dose, absorbed with a median lag time of 2.3 h. These PopPK estimates for fraction of total dose released from the second absorption compartment (VF2) and its lag time (ALAG2) were incorporated into the PBPK model, by splitting the doses in the oral administration protocols accordingly (63.4% of the dose at time = 0 h, 36.6% of the dose at time = 2.3 h). Then PBPK model building and parameter optimization was resumed, with greatly improved results. No other parameters of the PopPK analysis were used in the PBPK model.

During the DDIs with rifampicin and probenecid, a much faster rosuvastatin absorption and earlier C_max_ (t_max_ = 1.3–2.0 h) were observed in the rosuvastatin plasma concentration–time profiles [1, 28, 37, 38] (see Fig. 6). The rosuvastatin data of these DDI study arms were best described in the PopPK model using a single absorption compartment without a lag time. For the gemfibrozil DDI still two absorption compartments were required. Therefore, in the administration protocols of the PBPK model, only the rosuvastatin administration protocols for rosuvastatin monotherapy and gemfibrozil co-administration were split as described above; during rifampicin and probenecid co-treatment the total rosuvastatin dose was released at once (100% at time = 0 h).

### PBPK DDI Modeling and Evaluation

**Fig. 5:**
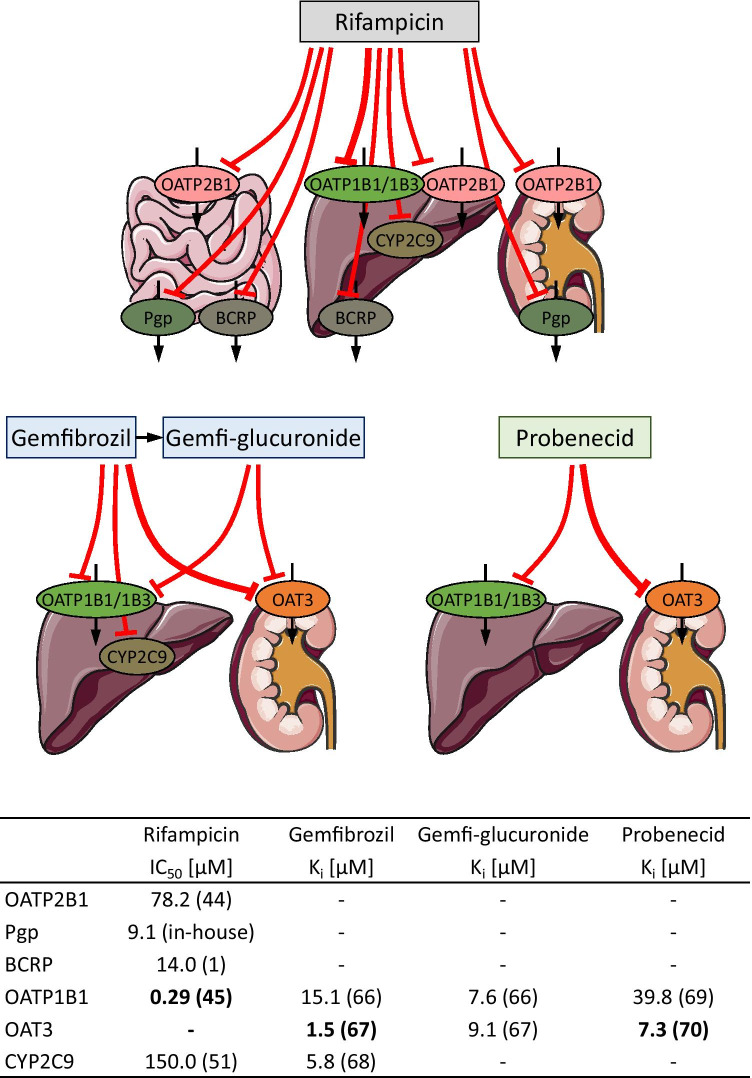
Modeled rosuvastatin DDIs. Schematic illustration of the rosuvastatin transporters and metabolic enzymes inhibited by rifampicin, gemfibrozil, gemfibrozil glucuronide and probenecid, with the in vitro inhibition constants applied for DDI modeling [1, 44, 45, 51, 66–70]. Numbers in parentheses refer to the in vitro literature references. Drawings by Servier, licensed under CC BY 3.0

Rosuvastatin DDIs with the perpetrator drugs rifampicin, gemfibrozil and probenecid were included in the model development. The interaction parameters to describe these DDIs were collected from in vitro literature and in-house inhibition measurements, and implemented into the previously developed and thoroughly tested perpetrator models [31–33]. To model the rifampicin-rosuvastatin DDI, competitive inhibition of OATP2B1, Pgp, BCRP, OATP1B1/1B3 and CYP2C9 by rifampicin was incorporated. To model the gemfibrozil-rosuvastatin DDI, competitive inhibition of OATP1B1/1B3, OAT3 and CYP2C9 by gemfibrozil and of OATP1B1/1B3 and OAT3 by gemfibrozil glucuronide was implemented. To model the probenecid-rosuvastatin DDI, competitive inhibition of OATP1B1/1B3 and OAT3 by probenecid was incorporated. To account for the absorption differences in the rifampicin and probenecid arms, the rosuvastatin dose during these DDIs was modeled as a single dose without a lag time, as indicated by the PopPK analysis. A schematic illustration of all modeled DDIs and the applied in vitro inhibition constants are shown in Fig. 5. Interaction parameters are also listed in the drug-dependent parameter tables of rifampicin, gemfibrozil and probenecid that are reproduced in ESM Tables S4.2.1, S5.2.1 and S6.2.1.

**Fig. 6:**
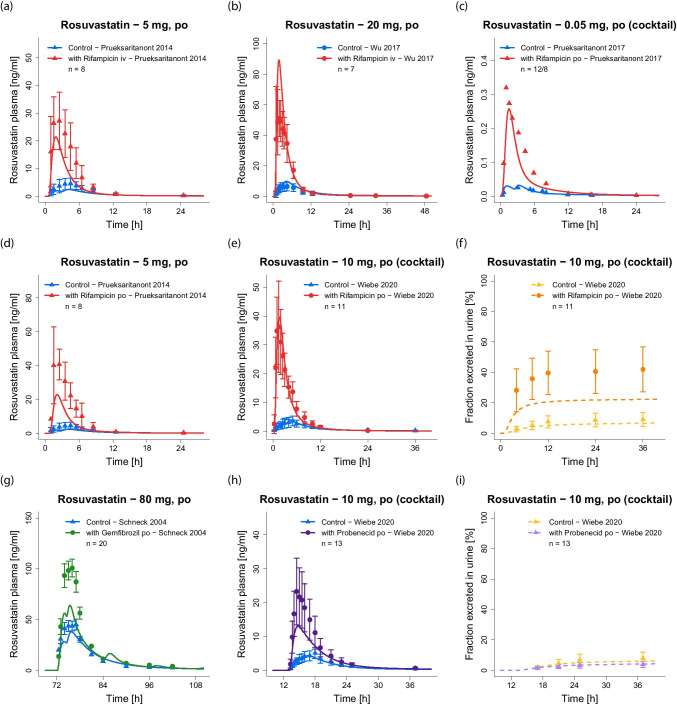
Rosuvastatin DDI model predictions. Simulated rosuvastatin profiles during (**a–b**) intravenous co-administration of rifampicin, (**c–f**) oral co-administration of rifampicin, (**g**) oral co-administration of gemfibrozil and (**h–i**) oral co-administration of probenecid, compared to observed data [1, 28, 29, 37, 38]. Simulations are shown as lines, clinical data are shown as dots (training dataset) or triangles (test dataset) ± standard deviation, if available. Details on dosing regimens and study populations are summarized in Tables S4.3.1, S5.3.1 and S6.3.1

The good predictive performance of the model for DDIs is illustrated in Fig. 6, showing the rosuvastatin plasma concentration–time profiles before and during perpetrator administration, compared to the respective clinical data of all 7 DDI studies. If fraction excreted unchanged in urine data were available, predicted and superimposed observed data are shown as well. Semilogarithmic plots of the plasma profiles are provided in the ESM (Figs. S4.4.1, S5.4.1 and S6.4.1).

**Fig. 7:**
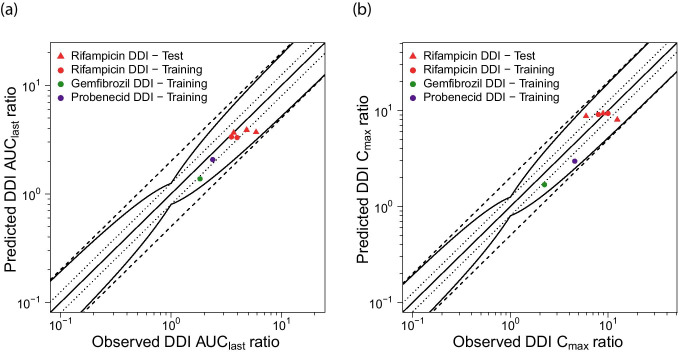
Rosuvastatin DDI model performance. Predicted compared to observed rosuvastatin (**a**) DDI AUC_last_ ratios and (**b**) DDI C_max_ ratios of all analyzed clinical DDI studies. The straight solid line marks the line of identity; curved lines show the DDI prediction success limits proposed by Guest et al. [39]. Dotted lines indicate 1.25-fold, dashed lines indicate twofold deviation. The individual DDI AUC_last_ ratios, DDI C_max_ ratios and GMFE values are listed in Tables S4.5.1, S5.5.1 and S6.5.1

A visualization of the DDI effects is shown in Fig. 7, comparing predicted with observed DDI AUC_last_ ratios and DDI C_max_ ratios of all analyzed studies. The modeled DDI ratios are within 1.6-fold of the observed data and within the DDI prediction success limits proposed by Guest et al. [39], with a GMFE of 1.20 (range 1.01–1.59) over all predicted DDI AUC_last_ ratios and of 1.32 (range 1.07–1.55) over all predicted DDI C_max_ ratios, further demonstrating the good DDI performance. All predicted and observed DDI ratios and GMFE values are listed in ESM Tables S4.5.1, S5.5.1 and S6.5.1.

## Discussion

A whole-body PBPK model of rosuvastatin that integrates the current mechanistic knowledge about the ADME processes that determine the pharmacokinetics of rosuvastatin was carefully built and evaluated. The established model adequately predicts the rosuvastatin plasma concentrations-time profiles over the full reported dosing range, fractions excreted unchanged in urine and feces, liver tissue and gallbladder PET data, as well as the DDI impact of the perpetrator drugs rifampicin, gemfibrozil and probenecid.

There are several previously published PBPK models of rosuvastatin [7, 25, 46–48], but only the model by Jamei et al. [47] and its extended version by Wang et al. [25] were applied for, and therefore challenged and evaluated by, DDI prediction. The development and refinement of this model were described in two very insightful publications; however, to capture the effects of the rifampicin-rosuvastatin, gemfibrozil-rosuvastatin and cyclosporine-rosuvastatin DDIs the authors reduced most of the applied in vitro Ki values by a factor of 10. To develop a model that shows good DDI performance using the measured in vitro inhibition constants, we included PET measured liver concentrations as well as data from clinical DDI studies into our training dataset, to get a better description of the fractions transported by OATP1B1/1B3, OAT3, BCRP and Pgp. This approach entails fixing the Ki values for the different DDIs to literature values and could not be applied to distinguish between OATP1B1 and OATP1B3, since their reported rifampicin Ki values are very similar. On the other hand, this similarity in Ki values (for rifampicin and probably gemfibrozil) allows to model these two transporters as one during DDI predictions. In addition, the contribution of OATP1B1 to the hepatic uptake of rosuvastatin is estimated at 80% or even higher [11, 12]. Nevertheless, if selective inhibitors for OATP1B1 or OATP1B3 emerge, the current model will overpredict the impact of selective OATP1B1 inhibitors and underpredict the DDI with selective OATP1B3 inhibitors. Further transporters that probably contribute to rosuvastatin transport are the sodium/taurocholate cotransporting polypeptide (NTCP), an uptake transporter at the basolateral membrane of hepatocytes similar to OATP1B1 and OATP1B3, and the multidrug resistance-associated protein 2 (MRP2), an efflux transporter at the apical membranes of liver, kidney, gastrointestinal tract and placenta similar to BCRP. The model could be extended to include these transporters when the relative contributions of the different transporters with similar function have been identified, or when clinical DDI studies with selective inhibitors become available to distinguish their impact on rosuvastatin pharmacokinetics. A previously modeled cyclosporine-rosuvastatin DDI study [25, 47] was not included in our analysis, as the only available clinical data [49] reports rosuvastatin plasma concentrations in heart transplant patients on chronic treatment with cyclosporine, prednisone and azathioprine, without a control group from the same clinical trial. The ritonavir-rosuvastatin DDI was not modeled, because in the reported clinical DDI studies, ritonavir was co-administered as a booster of atazanavir, darunavir, lopinavir or tipranavir [50], and these drugs are similarly potent inhibitors of OATP1B1 (and some also of OATP1B3 and Pgp) as ritonavir itself [51].

In addition to the DDI studies, we used ^11^C-rosuvastatin PET measured liver concentrations to inform the active rosuvastatin uptake into the liver [52]. The reported liver tissue concentrations following an intravenous ^11^C-rosuvastatin microdose bolus to a representative subject are adequately captured by our developed rosuvastatin model (see Figs. S3.4.3 and S3.4.4), and so are the concentrations in the plasma, whole blood (Figs. S3.4.3 and S3.4.4) and gallbladder (Fig. S3.4.5). The published kidney tissue concentrations are overpredicted by the model (approximately twofold, see Figs. S3.4.3 and S3.4.4), which could either be due to an overestimation of the OAT3 activity (OAT3 is expressed exclusively in the kidney, so that a wrong OAT3 reference concentration would be compensated by the optimized OAT3 transport rate constant), or to an underestimation of the active secretion into the renal tubules. However, the rosuvastatin fraction excreted unchanged to urine is well described. Possible reasons could be the expression level of Pgp in the kidney relative to other organs, as Pgp is the main transporter for rosuvastatin urinary secretion in the model, or missing unidentified transporters on either side of the kidney.

The DDI performance of our model using in vitro inhibition parameter values is summarized in Fig. 7, showing that the DDI AUC_last_ ratios of all three modeled DDIs are slightly underpredicted, but within the DDI prediction success limits proposed by Guest et al. [39]. The DDI C_max_ ratios of the rifampicin-rosuvastatin DDI (intravenous and oral rifampicin administration) are well predicted, those of the gemfibrozil-rosuvastatin and probenecid-rosuvastatin DDIs are also slightly underpredicted but within the limits of Guest et al. These small underpredictions of the DDI effects could be caused by the application of inaccurate in vitro inhibition constants (for some inhibitions there were no in vitro results available with rosuvastatin as the substrate) or by missing transporters in the model, for example for rosuvastatin uptake into the liver or kidney, that are inhibited more strongly by the applied perpetrator drugs than OATP1B1/1B3 or OAT3.

The delayed absorption of rosuvastatin evident in the plasma concentrations-time profiles (see Fig. 1) has been described previously [25], but the mechanistic reasons for the unusual shape of the rosuvastatin plasma profiles have not been elucidated, yet. As the passive permeability of rosuvastatin is low, absorption must be facilitated by transporters, but knowledge about transporters on both sides of the enterocytes is still limited. We implemented OATP2B1, Pgp and BCRP into the luminal gut membrane, but we were not able to describe the very slow rosuvastatin absorption and late C_max_ by fitting the activity of these transporters. Addition of the organic solute transporter (OST_α_/OST_β_) at the basolateral side of the enterocytes as described by Wang et al. [25] also did not help to capture the shape of the plasma profiles with our model. Although this is a desirable mechanistic approach, we decided against optimizing the expression of this transporter in all 11 intestinal compartments in PK-Sim to avoid identifiability issues and fitted the passive permeability out of the enterocytes instead. To investigate the delayed rosuvastatin absorption and late C_max_, a PopPK model was developed, utilizing individual rosuvastatin data. The individual profiles were well described by the final PopPK model using a split dose approach (see Figs. S2.4.3 to S2.4.6), and the median estimates for the split dose administration were subsequently incorporated into the PK-Sim administration protocols before the parameter optimization, greatly improving the fit of the PBPK model. No other parameters of the PopPK analysis were used in the PBPK model. This combined PopPK-PBPK approach has been applied previously, to successfully capture the double-peak phenomenon during administration of cimetidine in the fasted state [53]. There are several PopPK models of rosuvastatin in the literature, but they all use different methods to describe the rosuvastatin absorption (first-order absorption, sequential zero- and first-order absorption, simultaneous zero- and first-order absorption with different lag times) [54–56]. The published PBPK models that accomplished an adequate representation of the absorption phase also used different techniques to capture the delay in C_max_ (two sequential first-order absorption compartments, one first-order stomach and three first-order intestinal compartments, implementation of OST_α_/OST_β_ on the basolateral intestinal membrane with optimization of its expression levels in the 8 utilized intestinal compartments) [7, 25, 48], illustrating the current lack of knowledge that prevents a more mechanistic approach.

The observed shift of the rosuvastatin C_max_ to earlier time points during different DDIs has also been previously observed by Wang et al. [25] for the rifampicin-rosuvastatin (with intravenous and oral rifampicin) and cyclosporine-rosuvastatin DDIs, but the reasons for this shift are also not clear, yet. Inhibition of intestinal uptake transporters or of transporters that facilitate the subsequent basolateral transport from the enterocytes to the blood would not result in an earlier rosuvastatin C_max_ in blood plasma. Inhibition of intestinal Pgp and BCRP could contribute to a shorter t_max_, however, healthy volunteers expressing reduced function BCRP (*ABCG2* c.421AA genotype) do not exhibit an earlier rosuvastatin C_max_ than individuals with *ABCG2* c.421CC genotype [57]. Similarly, the typical shape of the rosuvastatin plasma concentration–time profiles is not altered in subjects expressing reduced function OATP1B1 (*SLCO1B1* c.521CC genotype) compared to wild-type (*SLCO1B1* c.521TT) individuals [58]. Macrolides, such as erythromycin, clarithromycin and azithromycin, were shown to stimulate gastric emptying and thereby to accelerate the absorption process in general [59, 60], but no direct evidence explaining the effects of rifampicin, cyclosporine or probenecid on rosuvastatin absorption could be found in the literature. Application of the developed PopPK model demonstrated that the rifampicin-rosuvastatin and probenecid-rosuvastatin DDIs were best described with only one absorption compartment without a lag time, supporting the hypothesis that these effects occur during the absorption phase of rosuvastatin. The co-administration arms of the rifampicin-rosuvastatin and probenecid-rosuvastatin DDI studies were therefore simulated without the split dose administration in the PBPK model. To predict DDIs with new drugs, both scenarios, with and without split dose administration, should be simulated. As soon as the first clinical DDI data become available, it can be judged from the rosuvastatin t_max_ during co-administration if the investigated perpetrator drug accelerates the absorption of rosuvastatin. One possible explanation could be an unknown rosuvastatin efflux transporter in the gut (in addition to Pgp and BCRP), that is strongly inhibited by rifampicin (intravenous and oral co-administration) and probenecid, but not by gemfibrozil.

In summary, the newly developed rosuvastatin PBPK model features the important processes governing rosuvastatin pharmacokinetics, namely OATP1B1/1B3 and BCRP transport for excretion in bile, and OAT3 and Pgp transport for excretion in urine. These transporters were implemented with their transport rate constants estimated in an optimization to a training dataset that included clinical blood, liver, gallbladder, feces, urine and DDI study data. Addition of OATP2B1 and CYP2C9 improved the model fit, and therefore these processes were retained in the final model. Future applications and model extensions include the modeling of transporter polymorphisms (for example as the cause of the observed ethnic differences), as soon as conclusive clinical data become available. To enable a more detailed representation of the absorption process, abundance/activity data of the responsible rosuvastatin transporters on both sides of the enterocytes are needed, as well as in vitro studies of their transport characteristics and their inhibition by perpetrator drugs. The continuing research regarding the function, isoforms, abundance and distribution of drug transporters and the development of more specific substrates and inhibitors to characterize their susceptibility to DDIs will steadily improve our understanding of ethnic differences, food effects, interindividual variability and DDI liabilities of drugs and will help us to refine our models and further improve their DDI performance.

## Conclusion

A mechanistic, whole-body PBPK model of the clinical OATP1B1/OATP1B3/BCRP substrate rosuvastatin for the investigation of transporter-mediated drug-drug interactions was carefully developed and evaluated. The model adequately (i) predicts the available clinical data of rosuvastatin in blood, liver (PET data), feces and urine, (ii) incorporates the important transporters involved in rosuvastatin pharmacokinetics and drug-drug interactions and (iii) mechanistically describes the impact of the clinical inhibitors rifampicin, gemfibrozil and probenecid on the pharmacokinetics of rosuvastatin, using published in vitro inhibition constants without adjustment. The model is shared in the Open Systems Pharmacology model repository (https://www.open-systems-pharmacology.org), to support future investigations of rosuvastatin pharmacokinetics, rosuvastatin therapy (for example during co-treatment with interacting drugs) and DDI studies during model-informed drug discovery and development.

## Supplementary Information

Below is the link to the electronic supplementary material.

Supplementary file1 (PDF 7.86 mb)

## Abbreviations

ADME: Absorption, distribution, metabolism and excretion
AUC: Area under the plasma concentration–time curve
AUC_last_: AUC from the time of drug administration to the last measured concentration
BCRP: Breast cancer resistance protein
C_max_: Peak plasma concentration
CYP: Cytochrome P450
DDI: Drug-drug interaction
EMA: European Medicines Agency
FDA: U.S. Food and Drug Administration
GFR: Glomerular filtration rate
GMFE: Geometric mean fold error
HMG-CoA reductase: 3-Hydroxy-3-methyl-glutaryl-coenzyme A reductase
iv: Intravenous administration
kcat: Catalytic or transport rate constant
Km: Michaelis–Menten constant
md: Multiple dose
MRD: Mean relative deviation
OAT: Organic anion transporter
OATP: Organic anion transporting polypeptide
PBPK: Physiologically based pharmacokinetic modeling
PET: Positron emission tomography
Pgp: P-glycoprotein
po: Oral administration
qd: Once daily
sd: Single dose
t_max_: Time to peak plasma concentration
