# Supplementary material for: Physiologically Based Pharmacokinetic Modeling of Rosuvastatin to Predict Transporter-Mediated Drug-Drug Interactions
Source: Pharm Res. 2021 Oct 18;38(10):1645–61. doi: 10.1007/s11095-021-03109-6 (PMC8602162; doi:10.1007/s11095-021-03109-6)
Supplement: Supplementary file 1 — Supplementary file1 (PDF 7.86 mb) [file 11095_2021_3109_MOESM1_ESM.pdf]

Pharmaceutical Research

Physiologically based pharmacokinetic modeling of rosuvastatin to predict transporter-mediated drug-drug interactions

Electronic Supplementary Material (ESM)

Nina Hanke [1], José David Gómez-Mantilla [1], Naoki Ishiguro [2], Peter Stopfer [1], Valerie Nock [1]

[1] Translational Medicine & Clinical Pharmacology, Boehringer Ingelheim Pharma GmbH & Co. KG, Biberach, Germany
[2] Kobe Pharma Research Institute, Nippon Boehringer Ingelheim Co. Ltd., Kobe, Japan

Funding
This project has received funding from Boehringer Ingelheim Pharma GmbH & Co. KG.

Conflict of Interest
All authors are employees of Boehringer Ingelheim.

Corresponding Author
Dr. Nina Hanke
Translational Medicine & Clinical Pharmacology, Boehringer Ingelheim Pharma GmbH & Co. KG
Birkendorfer Str. 65, 88397 Biberach, Germany
Phone: +49 7351 54 177 567
Email: nina.hanke@boehringer-ingelheim.com
ORCID: 0000-0001-6166-8838

Contents

1 Physiologically based pharmacokinetic (PBPK) modeling 4

1.1 PBPK model building . . . 4
1.2 Virtual individuals . . . 4
1.3 PBPK model evaluation . . . 4
1.4 PBPK model sensitivity analysis . . . 5
1.5 Mathematical implementation of drug-drug interactions . . . 5
1.5.1 Competitive inhibition . . . 5

2 Rosuvastatin population pharmacokinetic (PopPK) analysis 6

2.1 Background . . . 6
2.2 Objectives . . . 6
2.3 Methods . . . 6
2.3.1 Dataset . . . 6
2.3.2 Model building and evaluation . . . 7
2.4 Results . . . 7
2.4.1 Rosuvastatin PopPK model . . . 7
2.4.2 NONMEM code of the final rosuvastatin PopPK model . . . 16

3 Rosuvastatin 17

3.1 PBPK model development . . . 17
3.2 Rosuvastatin clinical studies . . . 18
3.3 Rosuvastatin drug-dependent parameters . . . 20
3.4 Profiles . . . 21
3.4.1 Semilogarithmic plots - Plasma . . . 21
3.4.2 Linear plots - Plasma . . . 26
3.4.3 Semilogarithmic plots – PET study . . . 31
3.4.4 Linear plots – PET study . . . 32
3.4.5 Linear plots – Fraction excreted . . . 33
3.5 Model evaluation . . . 34
3.5.1 Plasma concentration goodness-of-fit plots . . . 34
3.5.2 Mean relative deviation of plasma concentration predictions . . . 35
3.5.3 AUC_{last} and C_{max} goodness-of-fit plots . . . 36
3.5.4 Geometric mean fold error of predicted AUC_{last} and C_{max} values . . . 37
3.5.5 Sensitivity analysis . . . 39

4 Rifampicin-rosuvastatin drug-drug interaction (DDI) 40

4.1 DDI modeling . . . 40
4.2 Rifampicin drug-dependent parameters . . . 41
4.3 Rifampicin-rosuvastatin clinical DDI studies . . . 42
4.4 Profiles . . . 43
4.5 Model evaluation . . . 45
4.5.1 DDI AUC_{last} and DDI C_{max} ratio goodness-of-fit plots . . . 45
4.5.2 Geometric mean fold error of predicted DDI AUC_{last} and DDI C_{max} ratios . . . 46

5 Gemfibrozil-rosuvastatin drug-drug interaction (DDI) 47

5.1 DDI modeling . . . 47
5.2 Gemfibrozil and gemfibrozil 1-O-β-glucuronide drug-dependent parameters . . . 48
5.3 Gemfibrozil-rosuvastatin clinical DDI studies . . . 49

5.4 Profiles 50
5.5 Model evaluation 51
5.5.1 DDI AUC_{last} and DDI C_{max} ratio goodness-of-fit plots 51
5.5.2 Geometric mean fold error of predicted DDI AUC_{last} and DDI C_{max} ratios . . . 52

6 Probenecid-rosuvastatin drug-drug interaction (DDI) 53
6.1 DDI modeling 53
6.2 Probenecid drug-dependent parameters 54
6.3 Probenecid-rosuvastatin clinical DDI studies 55
6.4 Profiles 56
6.5 Model evaluation 57
6.5.1 DDI AUC_{last} and DDI C_{max} ratio goodness-of-fit plots 57
6.5.2 Geometric mean fold error of predicted DDI AUC_{last} and DDI C_{max} ratios . . . 58

7 System-dependent parameters 59

References 60

1 Physiologically based pharmacokinetic (PBPK) modeling

1.1 PBPK model building

PBPK model building was started with an extensive literature search to collect physicochemical parameters, information on absorption, distribution, metabolism and excretion (ADME) processes and clinical studies of intravenous and oral administration in single- and multiple-dose regimens. In addition to drug plasma concentration-time profiles, observed data on fraction excreted in urine or feces and tissue concentrations were integrated. The data of the clinical studies was digitized and divided into a training dataset for model building and a test dataset for model evaluation. The studies for the training dataset were selected to include intravenous and oral studies covering the whole published dosing range. If multiple studies of the same dose were available, studies with many participants, modern bioanalytical methods and frequent as well as late sampling were chosen for the training dataset. Model input parameters that could not be informed from literature were optimized by fitting the model simulations of all studies assigned to the training dataset simultaneously to their respective observed data.

1.2 Virtual individuals

Virtual mean individuals were generated for each study according to the published demographic information with corresponding age, weight, height, sex and ethnicity. If no information was provided, a default value was substituted (30 years of age, male, European, mean weight and height characteristics from the PK-Sim® population database). Enzymes and transporters relevant to the pharmacokinetics of rosuvastatin were incorporated in agreement with current literature, utilizing the PK-Sim® expression database [1] to define their relative expression in the different organs of the body. Details and references on the distribution and localization of the implemented metabolizing enzymes and drug transporters are provided in Table S7.0.1.

1.3 PBPK model evaluation

Model performance was evaluated with multiple methods. First, predicted plasma concentration-time profiles were compared visually with the data observed in the respective clinical studies. Second, the predicted plasma concentration values of all studies were plotted against their corresponding observed values in goodness-of-fit plots. In addition, model performance was evaluated by comparison of predicted to observed values of area under the plasma concentration-time curve from the time of drug administration to the last concentration measurement (AUC_{last}) and peak plasma concentrations (C_{max}).

As quantitative measures of the model performance, the mean relative deviation (MRD) of all predicted plasma concentrations (Equation S1) and the geometric mean fold error (GMFE) of all predicted AUC_{last} and C_{max} values (Equation S2) were calculated. MRD and GMFE values ≤ 2 characterize an adequate model performance.

$$MRD = 10^x \text{ with } x = \sqrt{\frac{1}{k}\sum_{i=1}^{k}(\log_{10} c_{\text{predicted},i} - \log_{10} c_{\text{observed},i})^2} \quad \text{(S1)}$$

where $c_{\text{predicted},i}$ = predicted plasma concentration, $c_{\text{observed},i}$ = corresponding observed plasma concentration, k = number of observed values.

$$GMFE = 10^x \text{ with } x = \frac{1}{m}\sum_{i=1}^{m}\left|\log_{10}\left(\frac{\text{predicted PK parameter}_i}{\text{observed PK parameter}_i}\right)\right| \quad \text{(S2)}$$

where predicted PK parameter$_i$ = predicted AUC_{last} or C_{max} value, observed PK parameter$_i$ = corresponding observed AUC_{last} or C_{max} value, m = number of studies.

Furthermore, the physiological plausibility of the parameter estimates and the results of a sensitivity analysis were assessed.

1.4 PBPK model sensitivity analysis

Sensitivity of the final model to single parameters (local sensitivity analysis) was calculated as relative change of AUC_{0-24} using the Sensitivity Analysis tool implemented in PK-Sim® [2]. Sensitivity analysis was performed applying a relative perturbation of 1000 % (variation range 10.0, maximum number of 9 steps). Parameters were included into the analysis if they were optimized, if they are associated with optimized parameters or if they might have a strong impact due to calculation methods used in the model.

Sensitivity to a parameter was calculated as the ratio of the relative change of the simulated AUC to the relative variation of the parameter around its value used in the final model according to Equation S3.

$$S = \frac{\Delta AUC}{AUC} \cdot \frac{p}{\Delta p} \tag{S3}$$

where S = sensitivity of the simulated AUC_{0-24} to the examined model parameter value, ΔAUC = change of the simulated AUC_{0-24}, AUC = simulated AUC_{0-24} with the original parameter value, Δp = change of the examined parameter value, p = original parameter value. A sensitivity of 0.5 signifies that a 100 % change of the examined parameter value causes a 50 % change of the simulated AUC_{0-24}.

1.5 Mathematical implementation of drug-drug interactions

1.5.1 Competitive inhibition

Competitive inhibitors reversibly bind to the active site of an enzyme or transporter and compete with the substrate for binding. Competitive inhibition can be overcome by high substrate concentrations (concentration-dependency); therefore, the maximum reaction velocity (v_{max}) remains unaffected, while the Michaelis-Menten constant (K_m) is increased by the inhibition ($K_{m,app}$, Equation S4). The reaction velocity (v) during co-administration of substrate and competitive inhibitor is described by Equation S5 [2]:

$$K_{m,app} = K_m \cdot \left(1 + \frac{[I]}{K_i}\right) \tag{S4}$$

$$v = \frac{v_{max} \cdot [S]}{K_{m,app} + [S]} \tag{S5}$$

where $K_{m,app}$ = Michaelis-Menten constant in the presence of the inhibitor, K_m = Michaelis-Menten constant, $[I]$ = free inhibitor concentration, K_i = dissociation constant of the inhibitor-enzyme or the inhibitor-transporter complex, v = reaction velocity, v_{max} = maximum reaction velocity, $[S]$ = free substrate concentration.

2 Rosuvastatin population pharmacokinetic (PopPK) analysis

2.1 Background

Typical rosuvastatin plasma concentration-time profiles show an unusual shape with a slow absorption phase and late C_{max} (t = 5.0 h). This delayed absorption has been described previously [3], but a mechanistic explanation could not be found in the literature. Therefore, rosuvastatin PBPK model building was supported by a population pharmacokinetic (PopPK) analysis to investigate and improve the description of the slow rosuvastatin absorption.

2.2 Objectives

The first objective of this analysis was to develop a PopPK model of rosuvastatin based on the digitized mean data from the only published intravenous study of rosuvastatin [4], and individual data from two oral studies [5, 6]. The model should focus on the description of the absorption phase and late C_{max} after oral rosuvastatin administration and support the PBPK model development.

In addition, during the DDIs with rifampicin and probenecid, a much faster rosuvastatin absorption and earlier C_{max} (t = 1.5 h) were observed, but not during the DDI with gemfibrozil. The second objective of this analysis was to extend the developed rosuvastatin PopPK model to analyze and describe the differences in absorption, bioavailability and clearance of rosuvastatin during the DDIs with rifampicin, probenecid and gemfibrozil, adding data of three different DDI studies to the dataset [7, 7, 8].

2.3 Methods

2.3.1 Dataset

For rosuvastatin PopPK model development, the mean data from the only published intravenous study of rosuvastatin [4] and individual rosuvastatin plasma concentration-time profiles from two oral rosuvastatin studies [5, 6], were used. For the DDI analysis, individual rosuvastatin plasma profiles before and during administration of rifampicin [7], individual rosuvastatin plasma profiles before and during administration of probenecid [7] and mean data of the only published study of the gemfibrozil-rosuvastatin DDI [8] were added to the dataset, see Table S2.3.1.

Table S2.3.1: Clinical rosuvastatin studies used for PopPK modeling

Study design	IDs	Age [years]	Weight [kg]	Height [cm]	Reference
8.0 mg rosuvastatin iv, 4 h (n=10)	1	36 (21–51)	78 (68–85)	177 (169–182)	Martin et al. 2003c [4]
10.0 mg rosuvastatin po, tab, fasted	19	37 (23–49)	85 (68–99)	181 (171–197)	Stopfer et al. 2016 [5]
10.0 mg rosuvastatin po, tab, fasted	25	35 (20–55)	84 (67–105)	179 (163–195)	Stopfer et al. 2018b [6]
Rifampicin-rosuvastatin DDI	11	40 (25–53)	86 (72–104)	181 (170–191)	Wiebe et al. 2020 [7]
Probenecid-rosuvastatin DDI	13	34 (21–51)	78 (62–95)	179 (169–186)	Wiebe et al. 2020 [7]
Gemfibrozil-rosuvastatin DDI (n=20)	1	41 ± 6	76 ± 10	172 ± 8	Schneck et al. 2004 [8]

IDs: number of plasma concentration-time profiles available for PopPK analysis, **iv**: intravenous, **n**: number of individuals studied for mean data digitized from literature, **po**: oral, **tab**: tablet

2.3.2 Model building and evaluation

Population pharmacokinetic analysis was performed using non-linear mixed-effects modeling techniques implemented in NONMEM (version 7.4.3). These allow estimation of population medians for pharmacokinetic model parameters with simultaneous quantification of interindividual variability (IIV). Model selection was based on the objective function value (OFV) provided by NONMEM, visual inspection of goodness-of-fit plots and the precision of parameter estimates. A nested model was considered superior to another when the OFV was reduced by 3.84 units (χ^2-test statistic, $p < 0.05$, 1 degree of freedom).

The First-Order Conditional Estimation with Interaction (FOCE-I) method was applied and models were coded in the ADVAN6 subroutine. For the structural base model one-, two- and three-compartment models were tested with first-order and saturated elimination (Michaelis-Menten) kinetics. Subsequently, different absorption models, such as zero-order, first-order and mixed parallel zero- and first-order absorption processes as well as split doses were evaluated. Saturable processes on absorption rates were tested using Michaelis-Menten kinetics. Based on the structural base model, IIVs were modeled exponentially and evaluated univariately. IIVs were added to the model if they improved the model in a statistically significant manner and if the parameter estimates of the model remained stable.

After the rosuvastatin model was established, the DDI profiles were added to the dataset and all data were modeled together using the same model with the DDI effects implemented via covariates on the model parameters of the initial model. For the DDI arms, covariate factors on the bioavailability and clearance were needed to account for the effects during the different DDIs. In addition, it was tested whether the absorption was influenced by the DDI.

2.4 Results

2.4.1 Rosuvastatin PopPK model

The pharmacokinetics of rosuvastatin were best described by a two-compartment model with first-order elimination (CL) from the central compartment. To describe the absorption phase and shape of the rosuvastatin plasma concentration-time profiles appropriately, the total rosuvastatin dose was split into a first (Dose 1) and a second dose (Dose 2), where the fraction of the second dose (VF2) was estimated and the fraction of the first dose was calculated as (1-VF2). Both doses were absorbed with the same absorption rate constant (Ka) and the same bioavailability, but the absorption of the second dose was delayed by a lag time (ALAG2). A schematic representation of the model is illustrated in Figure S2.4.1.

Parameter estimates of the final model are presented in Table S2.4.1 and the final NONMEM model code is provided in Section 2.4.2. All parameters were estimated precisely with relative standard errors (RSE) < 25%. Interindividual variability was incorporated on the bioavailability (Ftot), the fraction of dose attributed to the second dose (VF2), and the clearance (CL).

The final model adequately captures the observed rosuvastatin plasma concentrations-time profiles. 63.4% of the total dose were assigned to the first dose, absorbed directly without a lag time. The remaining 36.6% of the dose were assigned to the second dose with a lag time of 2.3 h. These estimated population median dose fractions and lag time were incorporated into the rosuvastatin PBPK model, by splitting the doses in all oral administration protocols accordingly. Then PBPK model building and parameter optimization was resumed, with greatly improved results. No other parameters of the PopPK analysis were used in the PBPK model.

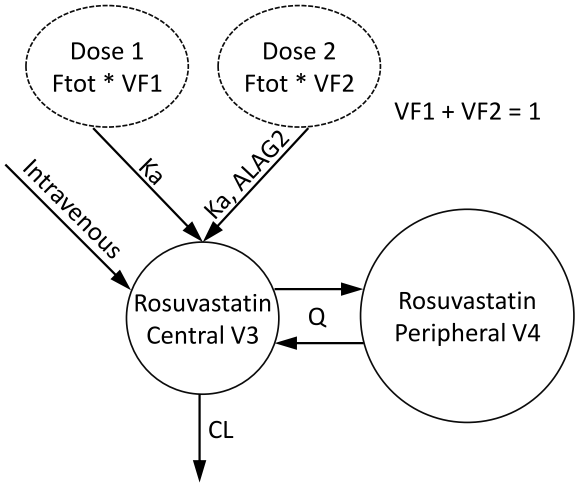

Figure S2.4.1: Schematic representation of the final rosuvastatin PopPK model

The final rosuvastatin PopPK model was then applied to investigate the rosuvastatin absorption phase during the different DDIs. Adding the data of the DDI studies to the PopPK dataset and using covariate factors on the bioavailability and clearance, the effects of the different DDIs could be well described. For the rifampicin and the probenecid DDIs, the second absorption process was no longer needed and a single absorption compartment described the data best. For gemfibrozil, two absorption compartments were still necessary. Therefore, only the rosuvastatin administration protocols for rosuvastatin monotherapy and during gemfibrozil co-administration were split as described above; during rifampicin and probenecid co-treatment the total rosuvastatin dose was released immediately. The parameter estimates between the model without and with DDI were comparable (Table S2.4.1).

Table S2.4.1: Parameter estimates of the final rosuvastatin PopPK model without and with DDI

		without DDI		with DDI		
**Parameter**	**Unit**	**Value**	**RSE [%]**	**Value**	**RSE [%]**	**Description**
Ka	1/h	0.464	4.8	0.397	5.8	Absorption rate constant
Ftot	%	7.93	10.8	8.40	9.0	Absolute bioavailability
VF2	-	0.366	7.0	0.338 *	7.2 *	Fraction of total dose attributed to 2nd dose
ALAG2	h	2.30	1.5	2.26 *	1.7 *	Lag time 2nd dose
CL	l/h	19.1	4.2	18.9	5.1	Clearance from the central volume
V3	l	79.4	1.7	83.2	3.0	Central volume of distribution
Q	l/h	12.0	7.5	12.5	5.6	Intercompartmental clearance
V4	l	199	11.8	211	10.1	Peripheral volume of distribution
IIV Ftot	%CV	80.1	9.0	69.8	8.4	Interindividual variability Ftot
IIV VF2	%CV	77.8	14.4	64.8	15.2	Interindividual variability VF2
IIV CL	%CV	26.1	22.7	27.1	18.2	Interindividual variability CL
Prop RE	%	22.2	5.8	26.9	3.5	Proportional residual error
Add RE	± ng/ml	0.00775	fixed	n.a.	n.a.	Additive residual error

***** no dose split and lag time for the rifampicin-rosuvastatin and probenecid-rosuvastatin DDI study arms,
RSE: relative standard error

Diagnostic goodness-of-fit plots (Figure S2.4.2) and plots of predicted rosuvastatin plasma concentration-time profiles compared to the corresponding observed data (Figures S2.4.3 to S2.4.8) demonstrate the good model performance.

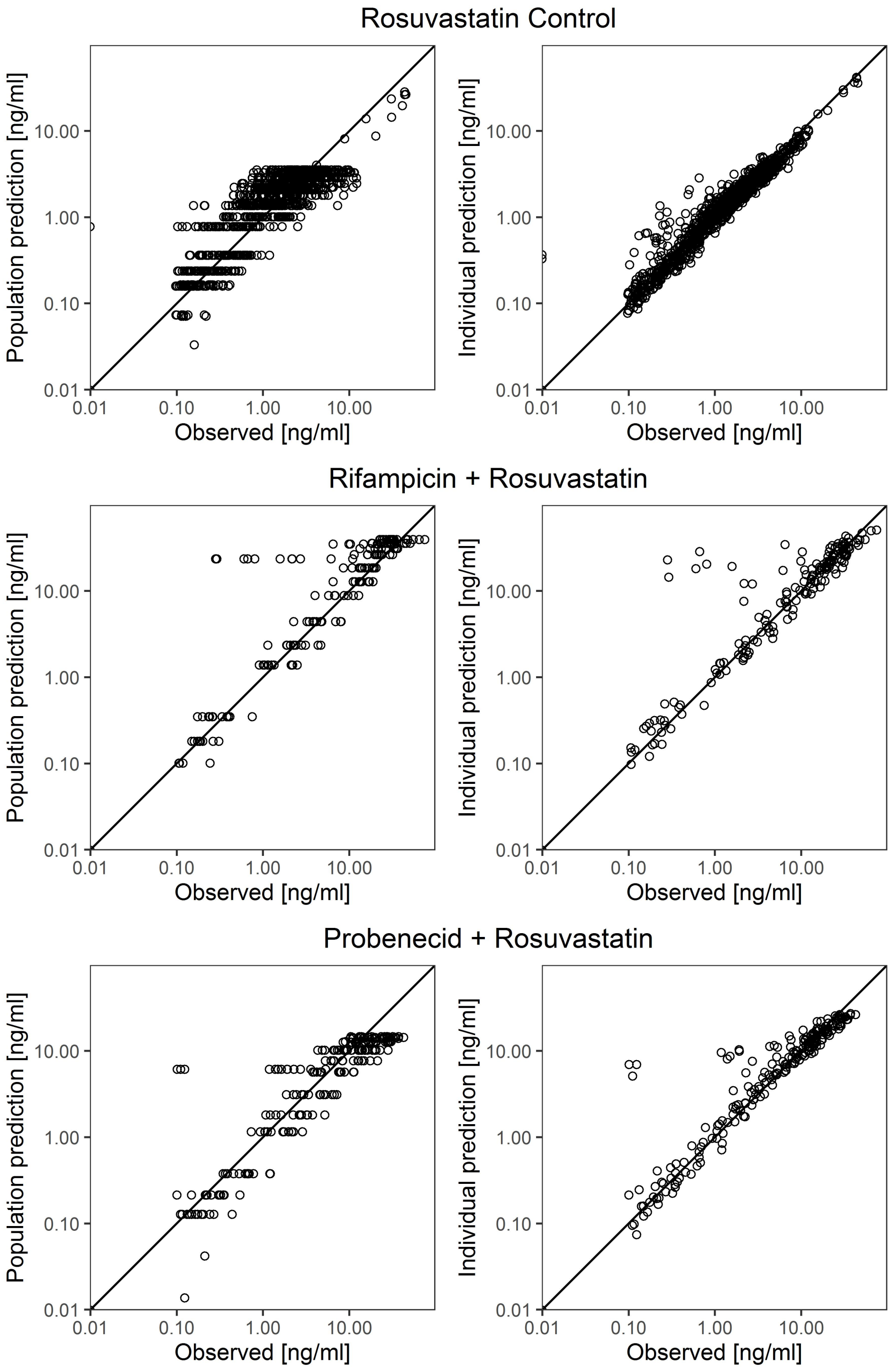

Figure S2.4.2: Goodness-of-fit plots of the rosuvastatin DDI PopPK model. Population (left) and individual (right) predictions versus observed concentrations. Upper panel: Rosuvastatin control study arms. Middle panel: Rosuvastatin plasma concentrations during co-administration of rifampicin. Lower panel: Rosuvastatin plasma concentrations during co-administration of probenecid. The diagonal lines indicate the line of identity

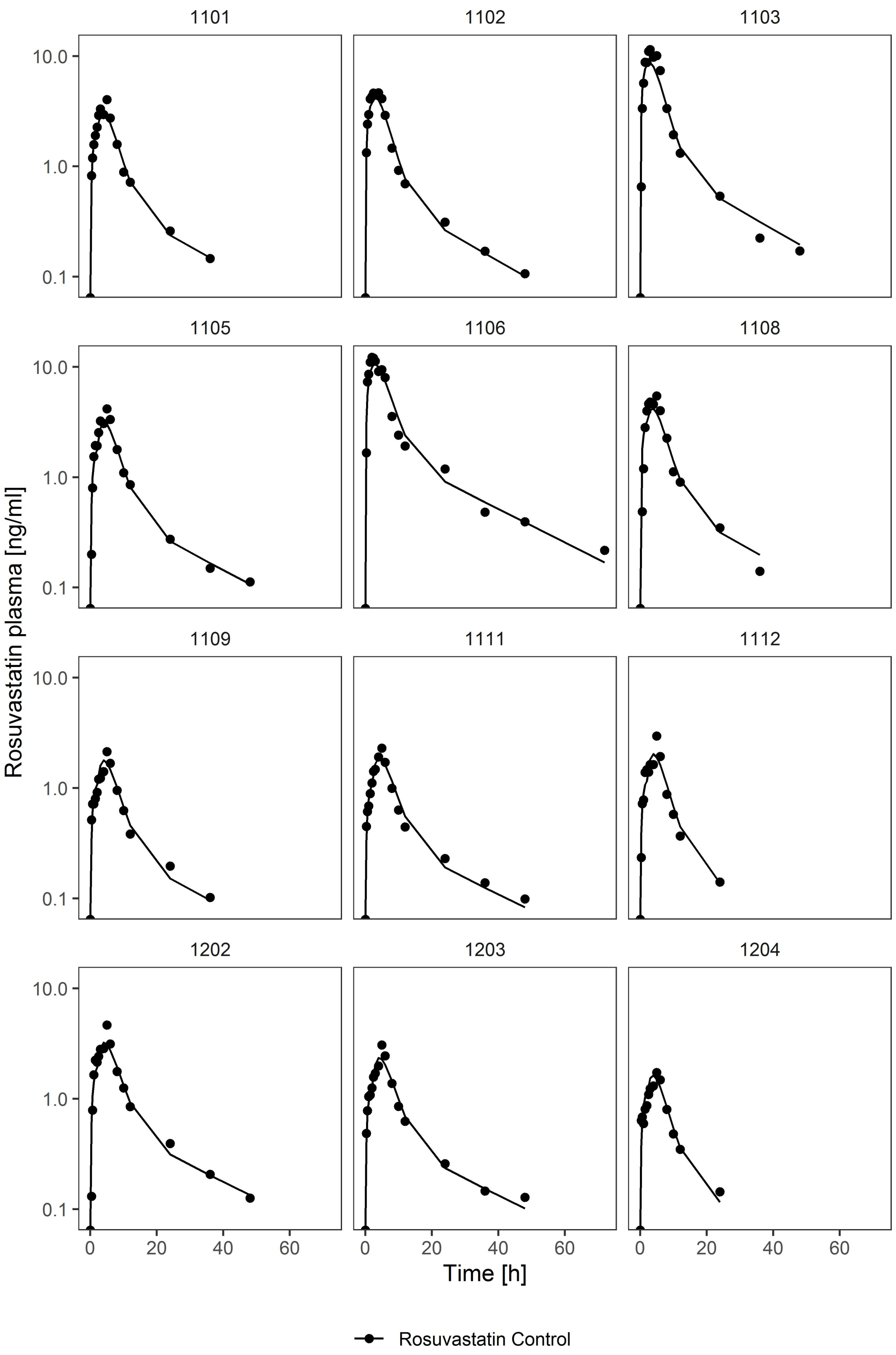

Figure S2.4.3: Plasma concentration-time profiles (observed data and individual predictions) of the rosuvastatin DDI PopPK model. Shown are the individuals 1101 - 1204 of the study by Stopfer et al. 2016 [5]

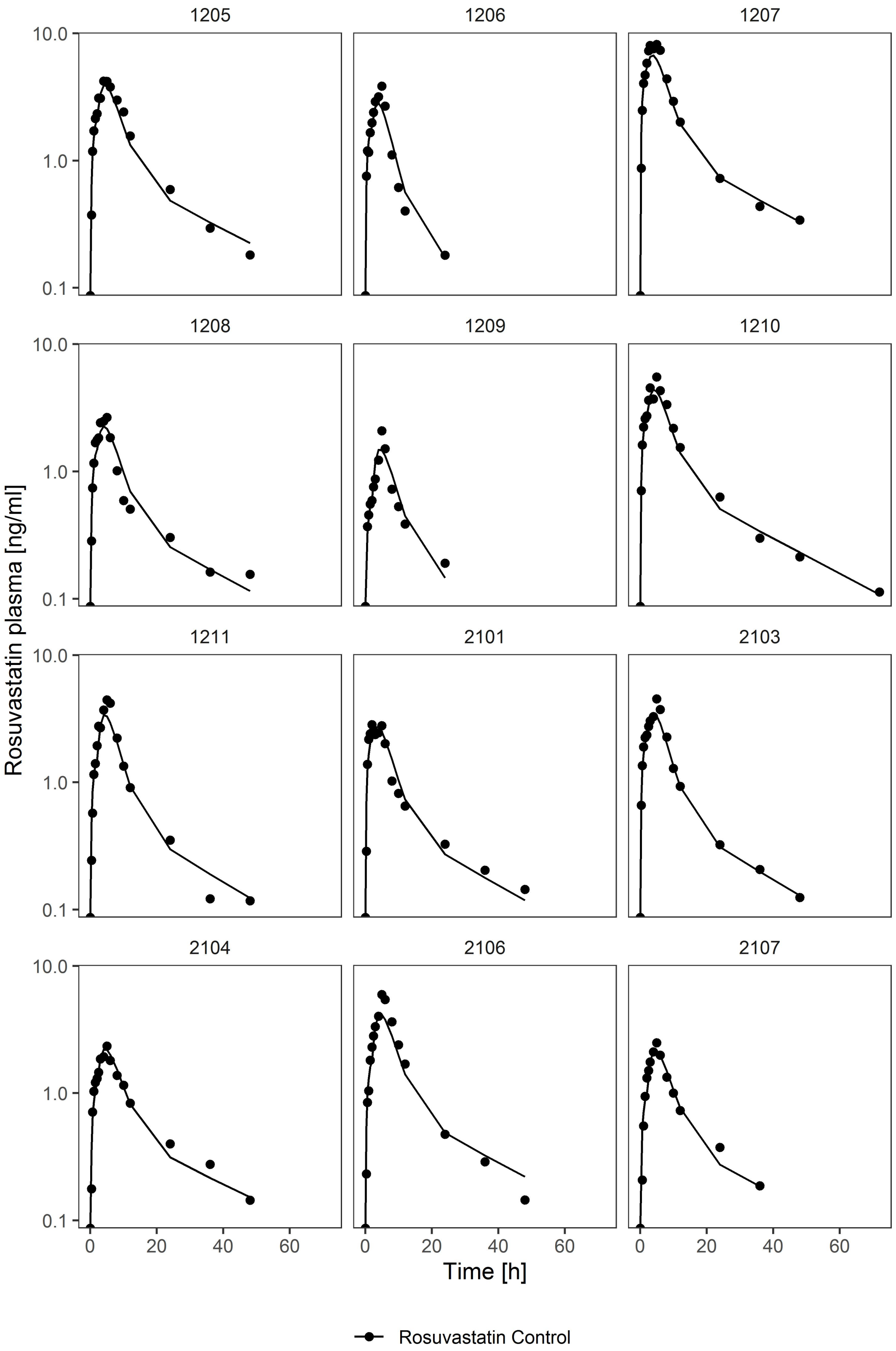

Figure S2.4.4: Plasma concentration-time profiles (observed data and individual predictions) of the rosuvastatin DDI PopPK model. Shown are the individuals 1205 - 1211 of the study by Stopfer et al. 2016 [5] and the individuals 2101 - 2107 of the study by Stopfer et al. 2018b [6]

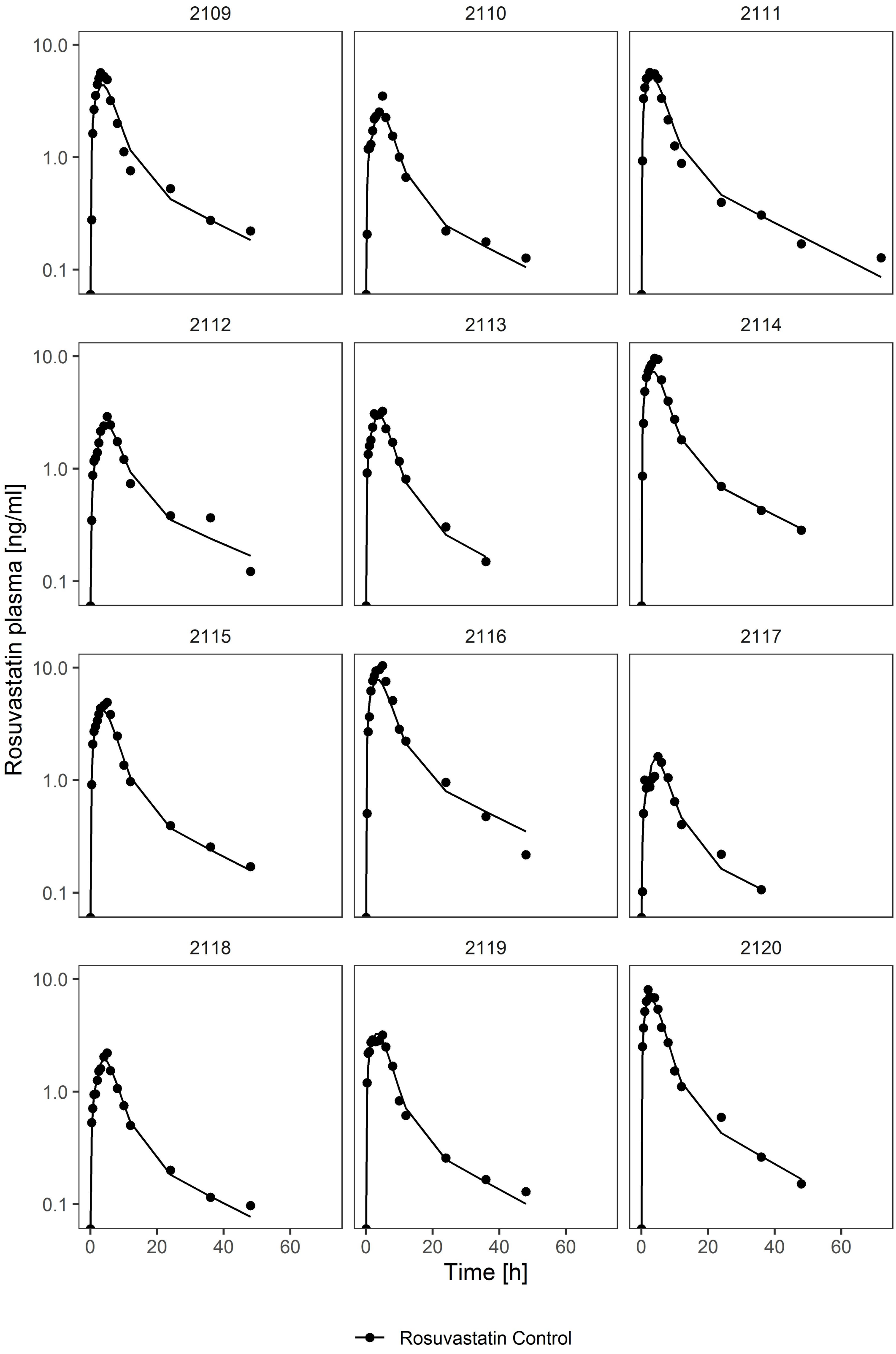

Figure S2.4.5: Plasma concentration-time profiles (observed data and individual predictions) of the rosuvastatin DDI PopPK model. Shown are the individuals 2109 - 2120 of the study by Stopfer et al. 2018b [6]

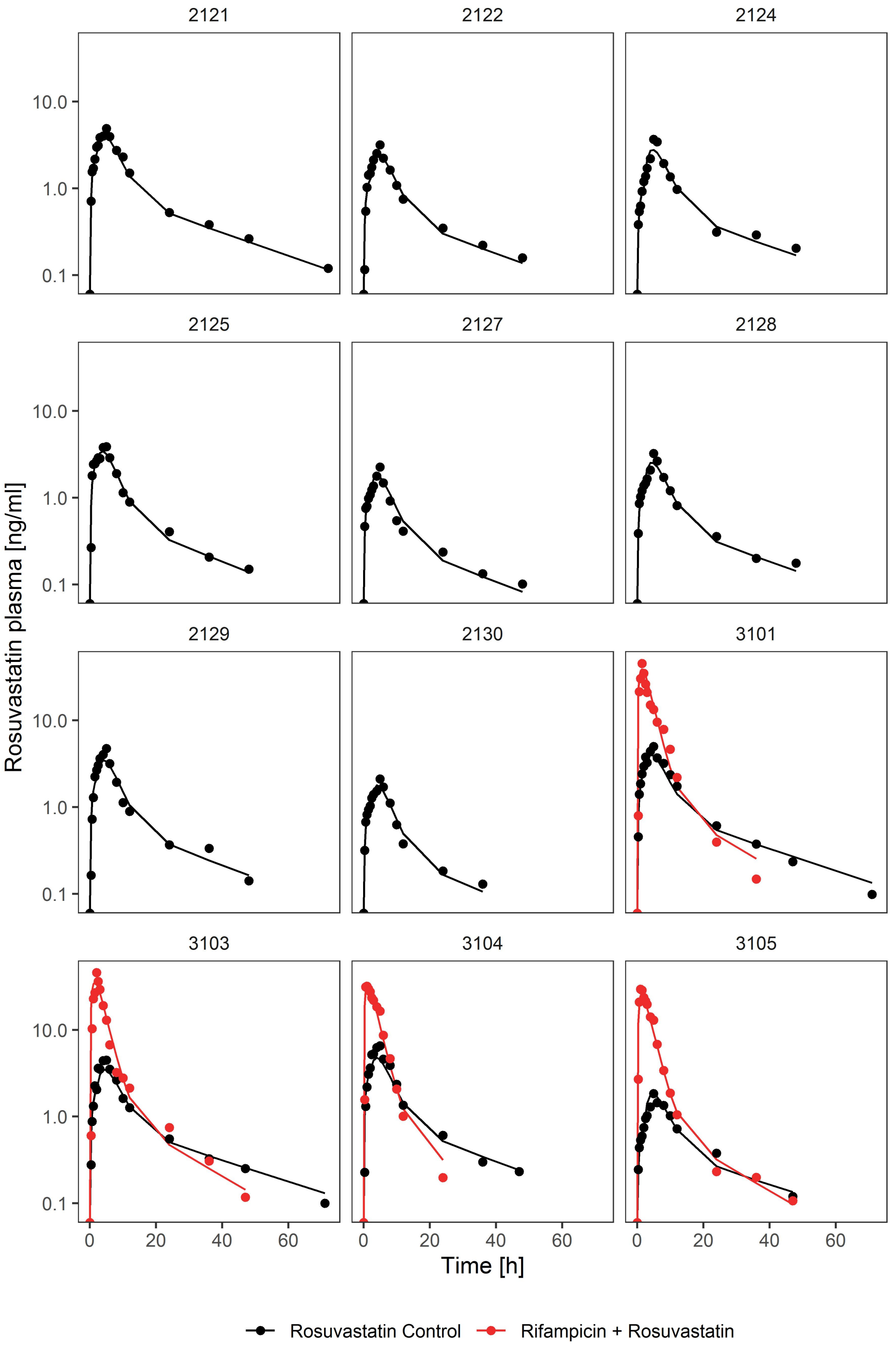

Figure S2.4.6: Plasma concentration-time profiles (observed data and individual predictions) of the rosuvastatin DDI PopPK model. Shown are the individuals 2121 - 2130 of the study by Stopfer et al. 2018b [6] and the individuals 3101 - 3105 of the study by Wiebe et al. 2020 [7] before and during rifampicin co-administration

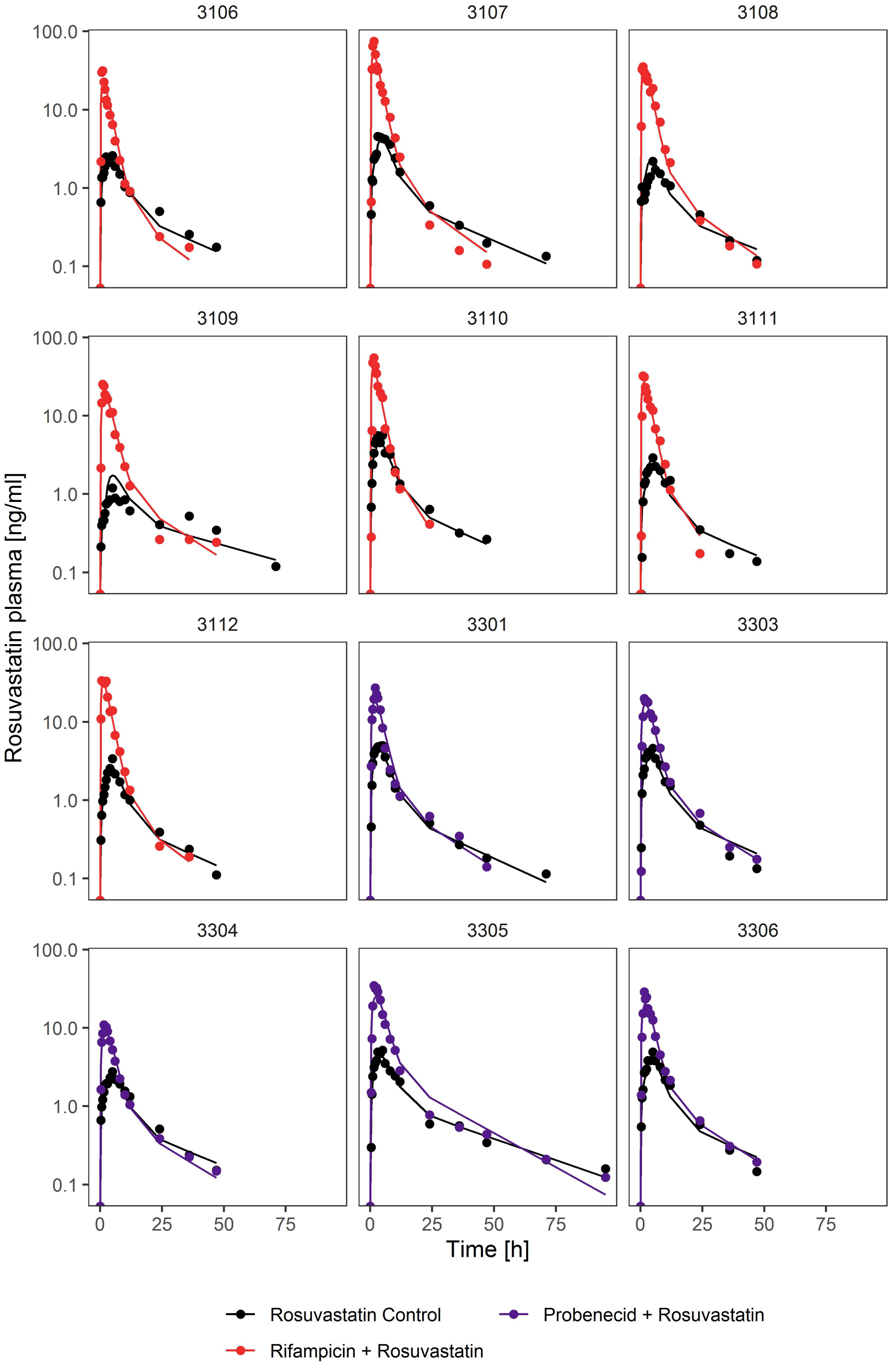

Figure S2.4.7: Plasma concentration-time profiles (observed data and individual predictions) of the rosuvastatin DDI PopPK model. Shown are the individuals 3106 - 3112 of the study by Wiebe et al. 2020 [7] before and during rifampicin co-administration and the individuals 3301 - 3306 of the study by Wiebe et al. 2020 [7] before and during probenecid co-administration

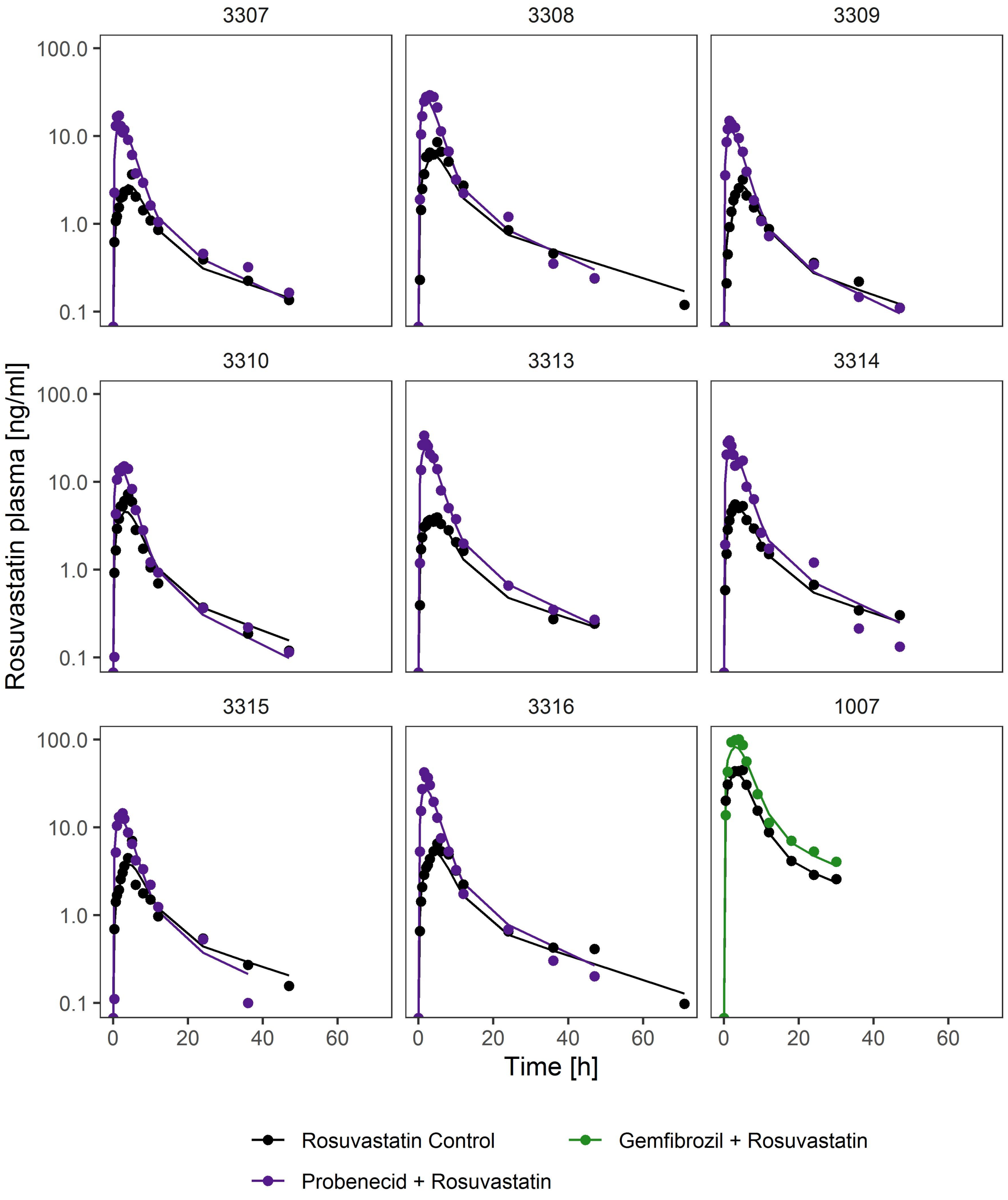

Figure S2.4.8: Plasma concentration-time profiles (observed data and individual predictions) of the rosuvastatin DDI PopPK model. Shown are the individuals 3307 - 3316 of the study by Wiebe et al. 2020 [7] before and during probenecid co-administration and the geometric mean plasma concentrations of the study by Schneck et al. 2004 [8] before and during gemfibrozil co-administration

2.4.2 NONMEM code of the final rosuvastatin PopPK model

```
$PROBLEM ROSUVASTATIN

$INPUT ID TIME TAD AMT RATE DOSE DV CMT MDV EVID STUDY

$DATA ../DATASET/DATASET_ROSUVASTATIN_V01.csv IGNORE=@
$SUBROUTINES ADVAN6 TOL=6

$MODEL
NCOMPARTMENTS = 4
COMP     = (DEPOT1)
COMP     = (DEPOT2)
COMP     = (CENTRAL, DEFOBS)
COMP     = (PERIPH1)

$PK
KA1      = THETA(1)
ALAG2    = THETA(2)
CL       = THETA(3)*EXP(ETA(2))                          ; Elimination from central
V3       = THETA(4)                                      ; Central volume
Q        = THETA(5)                                      ; Q
V4       = THETA(6)                                      ; Peripheral volume
S3       = V3/1000

FTOT     = THETA(7)
VF2      = THETA(8)

PHI_2    = LOG(VF2/(1 - VF2))
VF2_2    = EXP(PHI_2 + ETA(1))/(1 + EXP(PHI_2 + ETA(1)))
F2       = VF2_2*FTOT                                    ; Bioavailability of Depot 2

VF1      = (1 - VF2_2)
F1       = VF1*FTOT*EXP(ETA(3))                          ; Bioavailability of Depot 1

$DES
K30      = CL/V3
K34      = Q/V3
K43      = Q/V4

DADT(1) = - KA1*A(1)
DADT(2) = - KA1*A(2)
DADT(3) =   KA1*A(1) + KA1*A(2) - K30*A(3) - K34*A(3) + K43*A(4)
DADT(4) =                                    K34*A(3) - K43*A(4)

$ERROR
IPRED    = F
DEL      = 0
IF(IPRED.EQ.0) DEL = 0.0001

W        = F
IRES     = DV - IPRED
IWRES    = IRES/(W + DEL)
Y        = IPRED + W*EPS(1) + EPS(2)

$THETA
(0, 0.3)                        ; KA1
(0, 4)                          ; ALAG2
(0, 55)                         ; CL
(0, 70)                         ; V3
(0, 10)                         ; Q
(0, 300)                        ; V4
(0, 0.2, 1)                     ; FTOT
(0, 0.5)                        ; VF2

$OMEGA
0.1                             ; IIV VF2
0.01                            ; IIV CL
0.01                            ; IIV FTOT

$SIGMA
0.1                             ; Prop RE
0.00006 FIX                     ; Add RE

$EST METHOD=1 INTER MAXEVAL=9999 NOABORT PRINT=1 SIG=3 POSTHOC
$COV PRINT=E
$TABLE ID TIME TAD ETAS(1:LAST) DV IPRED IWRES CWRES EVID F1 F2 ALAG2 NOPRINT ONEHEADER FILE=sdtab0007b
```

3 Rosuvastatin

3.1 PBPK model development

The rosuvastatin model was established using 42 clinical studies, covering a broad dosing range of 0.00174 - 80 mg rosuvastatin (Table S3.2.1). The results of the PopPK analysis (absorption phase best described by a split dose approach with lag time for the second dose) were integrated into the PBPK simulations, using the estimated PopPK median split dose parameters in all oral rosuvastatin administration protocols in PK-Sim®, which greatly improved the results of the PBPK parameter identification. The final model applies active rosuvastatin transport by OATP2B1, OATP1B1/1B3, OAT3, Pgp and BCRP, as well as metabolism by CYP2C9 (Table S3.3.1). Details on the implementation of these drug transporters and metabolic enzymes in the different organs are provided in the system-dependent parameter table (Table S7.0.1)).

The good model performance is demonstrated in semilogarithmic (Figure S3.4.1) as well as linear plots (Figure S3.4.2) of predicted compared to observed plasma concentration-time profiles for all 42 clinical studies. Predicted compared to observed fractions excreted in urine, feces and bile are presented in Figure S3.4.5. In addition, goodness-of-fit plots comparing all predicted to their corresponding observed plasma concentrations are shown in Figure S3.5.1, and MRD values for each study are provided in Table S3.5.1. The correlation of predicted to observed AUC_{last} and C_{max} values is shown in Figure S3.5.2, and Table S3.5.2 lists the corresponding predicted and observed AUC_{last} and C_{max} values of all 42 studies including calculated GMFE values.

Sensitivity analysis (see Figure S3.5.3) revealed that the rosuvastatin model is sensitive to the values of rosuvastatin fraction unbound in plasma, lipophilicity, OATP1B1/1B3 K_m and Pgp K_m (all literature values) as well as intestinal permeability, OATP1B1/1B3 k_{cat} and Pgp k_{cat} (all optimized), confirming that the most impactful drug transport in the model is OATP1B1/1B3, followed by Pgp.

3.2 Rosuvastatin clinical studies

The clinical studies used for rosuvastatin model development and evaluation are summarized in Table S3.2.1.

Table S3.2.1: Rosuvastatin study table

Dose [mg]	Route	n	Male [%]	Age [years]	Weight [kg]	Height [cm]	BMI [kg/m^2]	Ethnicity	Dataset	Reference
0.00174 [a]	iv, bolus	6	33	26.17 ± 1.72	70.58 ± 9.06	172.3 ± 7.94	-	White American	training	Billington et al. 2019 [9]
8.0	iv, 4 h	10	100	35.7 (21-51)	77.6 (68-85)	177 (169-182)	-	European	training	Martin et al. 2003c [4]
0.05 [b]	po, sol	12	-	(19-55)	-	-	(19-32)	American	test	Prueksaritanont et al. 2017 [10]
5.0	po, -	8	-	(19-55)	-	-	(19-32)	American	test	Prueksaritanont et al. 2014 [11]
10.0	po, caps	12	100	33 (22-42)	80 (68-92)	176 (167-189)	-	European	test	Cooper et al. 2003b [12]
10.0	po, tab	20	100	41 ± 13	82 ± 9	183 ± 7	25 ± 3	White American	test	Csonka et al. 2019 [13]
10.0	po, tab	16	100	28 ± 8 (21-54)	(64-92)	-	24 ± 2 (21-29)	Canadian	test	Huguet et al. 2016 [14]
10.0	po, tab	18	100	41 (31-60)	82 (63-107)	178 (170-189)	-	European	test	Martin et al. 2003b [15]
10.0	po, tab	19	100	37 ± 8 (23-49)	85 ± 7 (68-99)	181 ± 7 (171-197)	26 ± 2 (23-29)	European	training	Stopfer et al. 2016 [5]
10.0	po, tab	13	100	33 ± 11 (19-52)	87 ± 10 (71-104)	182 ± 6 (173-193)	26 ± 2 (22-30)	European	test	Stopfer et al. 2018a [16]
10.0	po, tab	25	100	35 ± 10 (20-55)	84 ± 9 (67-105)	179 ± 7 (163-195)	26 ± 2 (22-29)	European	training	Stopfer et al. 2018b [6]
10.0 [c]	po, tab	11	100	40 ± 12 (25-53)	86 ± 10 (72-104)	181 ± 6 (170-191)	26 ± 3 (22-29)	European	test	Wiebe et al. 2020 [7]
10.0 [c]	po, tab	13	100	34 ± 10 (21-51)	78 ± 10 (62-95)	179 ± 5 (169-186)	24 ± 3 (19-29)	European	test	Wiebe et al. 2020 [7]
10.0	po, -	49	100	38 (20-54)	80 (59-105)	171 (153-184)	-	American	test	Coss et al. 2016 [17]
20.0	po, sol	6	100	44 (36-52)	87 (74-99)	180 (174-185)	-	European	test	Martin et al. 2003d [18]
20.0	po, caps	6	-	-	-	-	-	-	test	FDA et al. 2003 [19]
20.0	po, tab	48	75	33 ± 10	78 ± 13	172 ± 11	26 ± 2	White American	test	Edwards et al. 2017 [20]
20.0	po, tab	9	100	41 (31-60)	82 (63-107)	178 (170-189)	-	European	test	Martin et al. 2003b [15]
20.0	po, tab	7	57	43 ± 14	68 ± 10	-	24 ± 2	White American	training	Wu et al. 2017 [21]
20.0	po, -	29	70	24 ± 11.3	73 ± 10	176 ± 8	24 ± 3	White American	test	Birmingham et al. 2015 [22]
20.0	po, -	16	56	46 (26-58)	77 (63-92)	167 (154-183)	28 (22-31)	White American	test	Jones et al. 2020 [23]
20.0	po, -	35	100	36 ± 10.5	85 ± 10.0	177 ± 8	-	White American	test	Lee et al. 2018 [24]
20.0	po, -	21	95	33 ± 10	84 ± 11	-	26 ± 3	European	test	Martin et al. 2016 [25]
20.0	po, -	31	52	45 ± 13 (20-64)	-	-	26 ± 3	White American	test	Willis et al. 2020 [26]
40.0	po, caps	8	100	(18-33)	(55-88)	(153-188)	-	European	test	Martin et al. 2002a [27]
40.0	po, caps	8	0	(18-33)	(55-88)	(153-188)	-	European	test	Martin et al. 2002a [27]
40.0	po, tab	33	67	31 ± 11 (18-50)	78 ± 12 (53-106)	175 ± 10 (157-200)	25 ± 3 (20-30)	White American	training	Gidal et al. 2017 [28]
40.0	po, tab	9	100	41 (31-60)	82 (63-107)	178 (170-189)	-	European	test	Martin et al. 2003b [15]
40.0	po, tab	10	100	36 (21-51)	78 (68-85)	177 (169-182)	-	European	training	Martin et al. 2003c [4]
40.0	po, -	36	86	30 ± 9	75 ± 11	179 ± 7	23 ± 3	European	test	Lee et al. 2005 [29]

[a] 160 minutes earlier a 5 mg rosuvastatin oral dose was given, to ensure pharmacokinetics comparable to clinical doses, [b] administered as microdose cocktail together with 0.01 mg midazolam, 0.375 mg dabigatran etexilate, 0.01 mg pitavastatin and 0.1 mg atorvastatin, [c] administered as cocktail together with 0.25 mg digoxin, 1 mg furosemide and 10 mg metformin, **-**: not given, **BMI**: body mass index, **caps**: capsule, **iv**: intravenous, **n**: number of individuals studied, **po**: oral, **qd**: once daily, **sol**: solution, **tab**: tablet, **test**: test dataset (model evaluation), **training**: training dataset (model development and parameter optimization)

Table S3.2.1: Rosuvastatin study table *(continued)*

Dose [mg]	Route	n	Male [%]	Age [years]	Weight [kg]	Height [cm]	BMI [kg/m^2]	Ethnicity	Dataset	Reference
80.0	po, tab	14	100	37 (29-51)	81 (70-92)	179 (172-188)	-	European	test	Cooper et al. 2002 [30]
80.0	po, tab	13	100	24 (21-31)	74 (60-83)	182 (170-189)	-	European	training	Cooper et al. 2003a [31]
80.0	po, tab	14	100	38 (25-56)	78 (59-88)	178 (168-186)	-	European	test	Cooper et al. 2003b [12]
80.0	po, tab	14	100	33 (22-44)	76 (59-98)	179 (164-190)	-	European	test	Cooper et al. 2003c [32]
80.0	po, tab	18	100	41 (31-60)	82 (63-107)	178 (170-189)	-	European	test	Martin et al. 2003b [15]
80.0	po, tab	20	85	41 ± 6	76 ± 10	172 ± 8	-	American	test	Schneck et al. 2004 [8]
10.0	po, -, qd	12	50	51 ± 8 (26-56)	81 ± 10 (62-96)	-	-	European	test	Kosoglou et al. 2004 [33]
10.0	po, -, qd	21	92	39 (19-61)	77 (57-100)	-	-	White American	test	Martin et al. 2002b [34]
20.0	po, caps, qd	6	-	-	-	-	-	-	test	FDA et al. 2003 [19]
20.0	po, -, qd	10	0	-	-	-	-	-	test	FDA et al. 2018 [35]
20.0	po, -, qd	32	100	36 ± 11	85 ± 10.0	177 ± 8	-	White American	training	Lee et al. 2018 [24]
40.0	po, -, qd	42	75	37 ± 9 (22-55)	72 ± 10	172 ± 10	-	Canadian	training	Gosai et al. 2008 [36]

[a] 160 minutes earlier a 5 mg rosuvastatin oral dose was given, to ensure pharmacokinetics comparable to clinical doses, [b] administered as microdose cocktail together with 0.01 mg midazolam, 0.375 mg dabigatran etexilate, 0.01 mg pitavastatin and 0.1 mg atorvastatin, [c] administered as cocktail together with 0.25 mg digoxin, 1 mg furosemide and 10 mg metformin, **-**: not given, **BMI**: body mass index, **caps**: capsule, **iv**: intravenous, **n**: number of individuals studied, **po**: oral, **qd**: once daily, **sol**: solution, **tab**: tablet, **test**: test dataset (model evaluation), **training**: training dataset (model development and parameter optimization)

3.3 Rosuvastatin drug-dependent parameters

The drug-dependent parameters of the final rosuvastatin model are summarized in Table S3.3.1. The associated system-dependent parameters are listed in Table S7.0.1.

Table S3.3.1: Rosuvastatin drug-dependent parameters

Parameter	Model	Unit	Source	Literature	Reference	Description
MW	481.54	g/mol	Literature	481.54	[37]	Molecular weight
pKa (acid)	4.3	-	Literature	4.3	[38]	Acid dissociation constant
Solubility (water)	7.8	g/l	Literature	7.8	[39]	Solubility
logP	-0.33	-	Literature	-0.33	[40, 41]	Lipophilicity
fu	11.5	%	Literature	11.5	[42]	Fraction unbound
BCRP K_m	2.02	µmol/l	Literature	2.02	[43]	BCRP Michaelis-Menten constant
BCRP k_{cat}	1.01	1/min	Optimized	-	-	BCRP transport rate constant
CYP2C9 CL_{spec}	1.04E-03	l/µmol·min	Optimized	-	-	CYP2C9 first-order clearance
OAT3 K_m	7.40	µmol/l	Literature	7.40	[44]	OAT3 Michaelis-Menten constant
OAT3 k_{cat}	66851.33	1/min	Optimized	-	-	OAT3 transport rate constant
OATP1B1/1B3 K_m	0.80	µmol/l	Literature	0.80	[43]	OATP1B1/1B3 Michaelis-Menten constant
OATP1B1/1B3 k_{cat}	1124.12	1/min	Optimized	-	-	OATP1B1/1B3 transport rate constant
OATP2B1 K_m	6.42	µmol/l	Literature	6.42	[43]	OATP2B1 Michaelis-Menten constant
OATP2B1 k_{cat}	5.00	1/min	Optimized	-	-	OATP2B1 catalytic rate constant
Pgp K_m	203.00	µmol/l	Literature	203.00	[45]	Pgp Michaelis-Menten constant
Pgp k_{cat}	36.07	1/min	Optimized	-	-	Pgp transport rate constant
GFR fraction	1.00	-	Assumed	-	-	Fraction of filtered drug in the urine
EHC continuous fraction	1.00	-	Assumed	-	-	Fraction of bile continually released
Partition coefficients	Diverse	-	Calculated	PK-Sim	[2]	Cell to plasma partition coefficients
Cellular permeability	1.34E-06	cm/min	Calculated	PK-Sim	[2]	Permeability into the cellular space
Intestinal permeability	1.51E-06	cm/min	Optimized	7.44E-09	Calculated	Transcellular intestinal permeability
P(intracell→interstitial) small intestine	4.31E-04	cm/min	Optimized	2.72E-04	Calculated	Basolateral permeability out of the small intestinal mucosa cells
P(intracell→interstitial) large intestine	3.66E-06	cm/min	Optimized	2.72E-04	Calculated	Basolateral permeability out of the large intestinal mucosa cells
Tablet dissolution Weibull shape	1.77	-	Literature	Extracted (Fig.4b)	[46]	Dissolution profile shape
Tablet dissolution Weibull time	6.25	min	Literature	Extracted (Fig.4b)	[46]	Dissolution time (50% dissolved)

BCRP: breast cancer resistance protein, **CYP2C9**: cytochrome P450 2C9, **EHC**: enterohepatic circulation, **GFR**: glomerular filtration rate, **OAT3**: organic anion transporter 3, **OATP1B1/1B3**: organic anion transporting polypeptide 1B1/1B3, **OATP2B1**: organic anion transporting polypeptide 2B1, **Pgp**: P-glycoprotein, **PK-Sim**: PK-Sim standard calculation method

3.4 Profiles

3.4.1 Semilogarithmic plots - Plasma

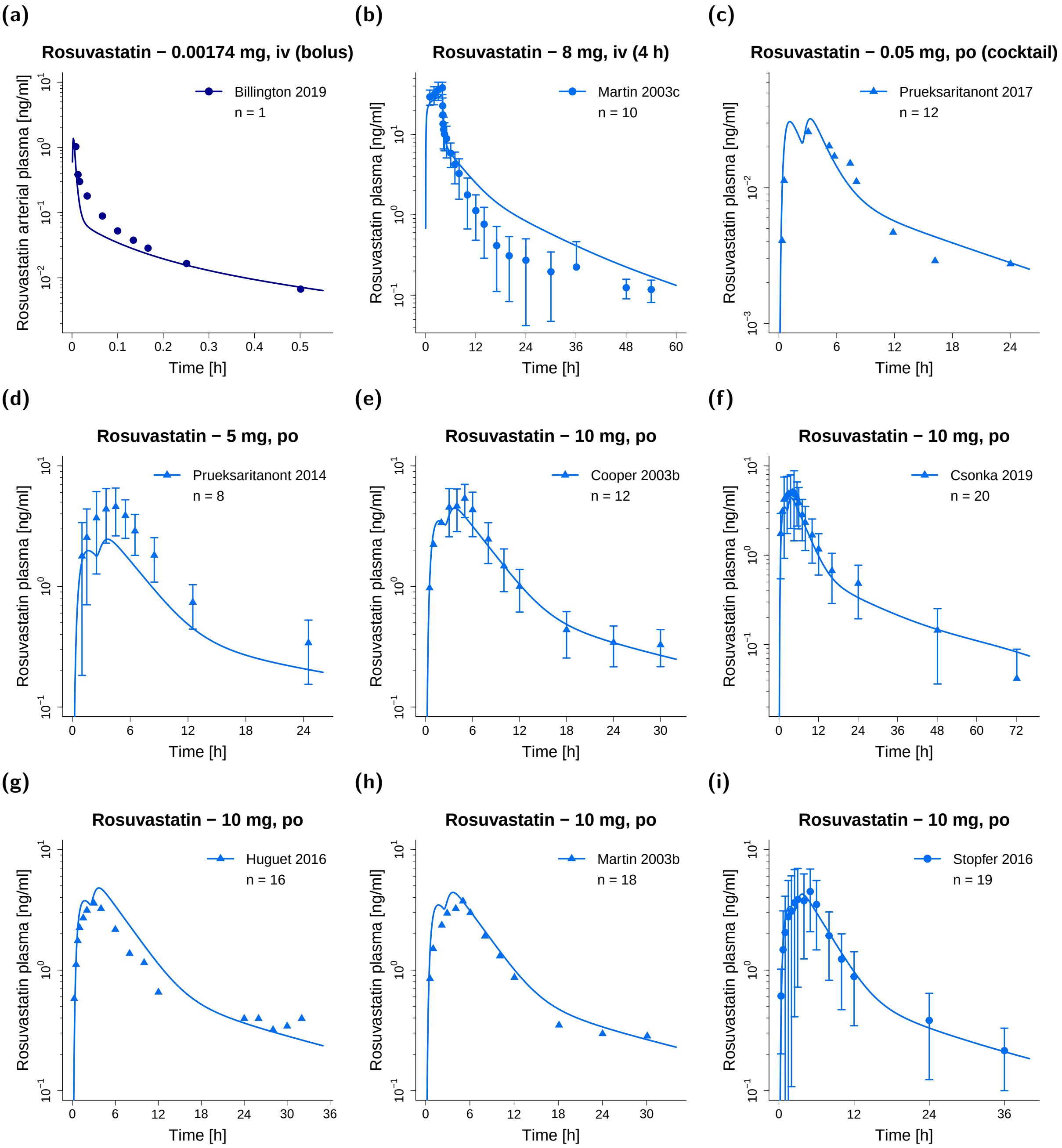

Figure S3.4.1: Rosuvastatin plasma concentration-time profiles (semilogarithmic). Simulations are shown as lines, observed data are shown as dots (training dataset) or triangles (test dataset) ± SD, if available. Details on administration protocols, study populations and literature references are listed in Table S3.2.1

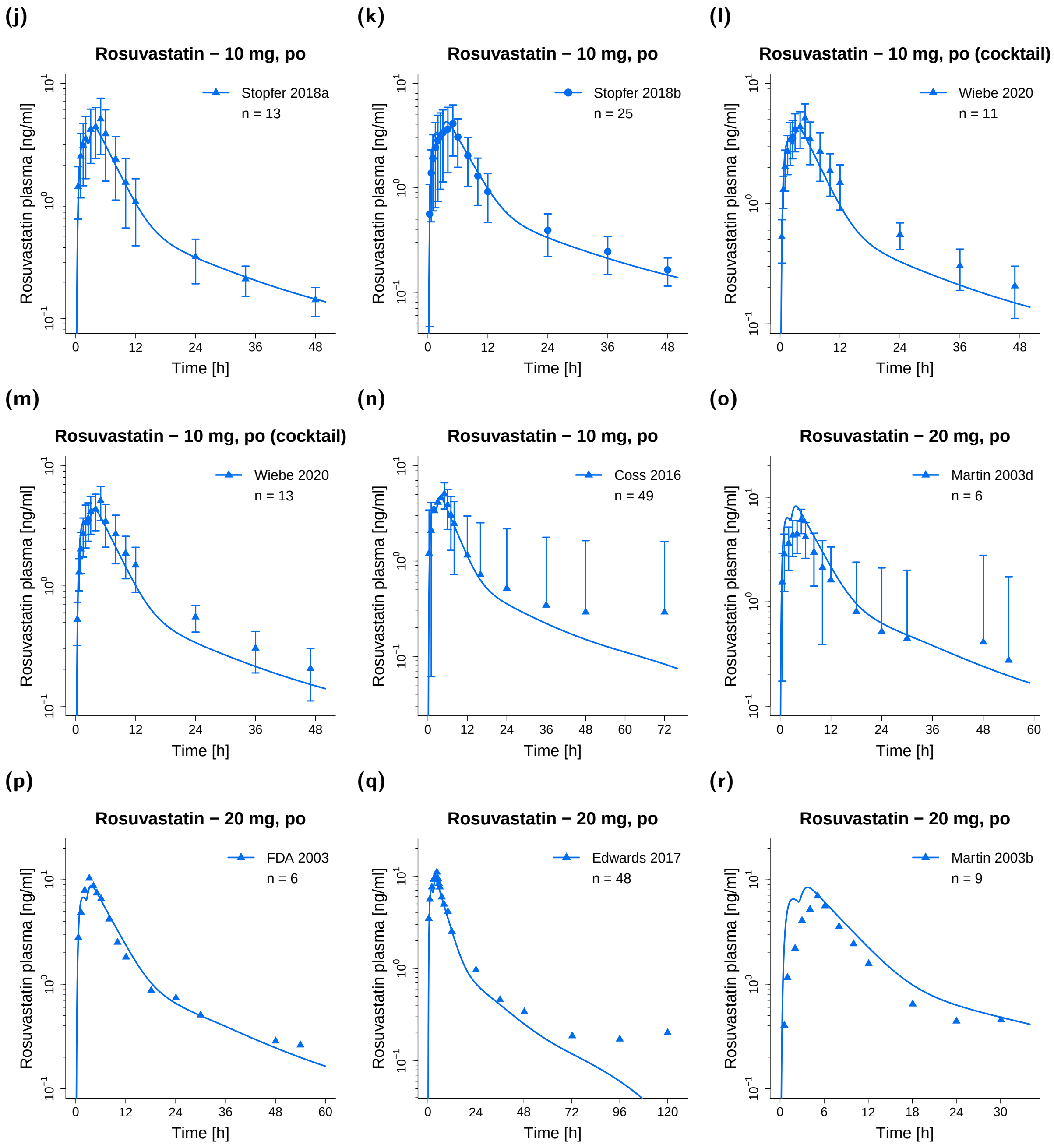

Figure S3.4.1: Rosuvastatin plasma concentration-time profiles (semilogarithmic). Simulations are shown as lines, observed data are shown as dots (training dataset) or triangles (test dataset) ± SD, if available. Details on administration protocols, study populations and literature references are listed in Table S3.2.1 (continued)

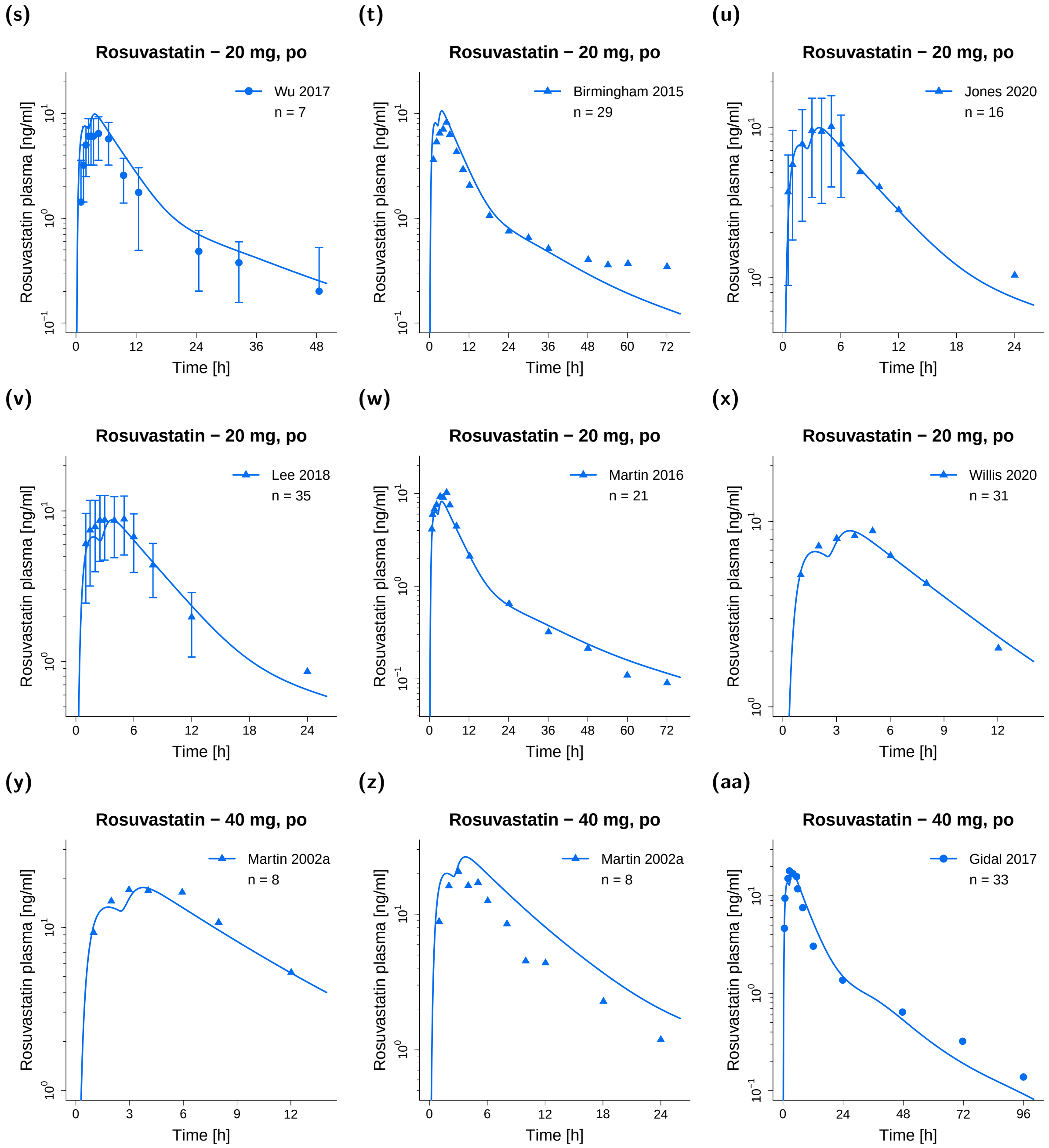

Figure S3.4.1: Rosuvastatin plasma concentration-time profiles (semilogarithmic). Simulations are shown as lines, observed data are shown as dots (training dataset) or triangles (test dataset) ± SD, if available. Details on administration protocols, study populations and literature references are listed in Table S3.2.1 (continued)

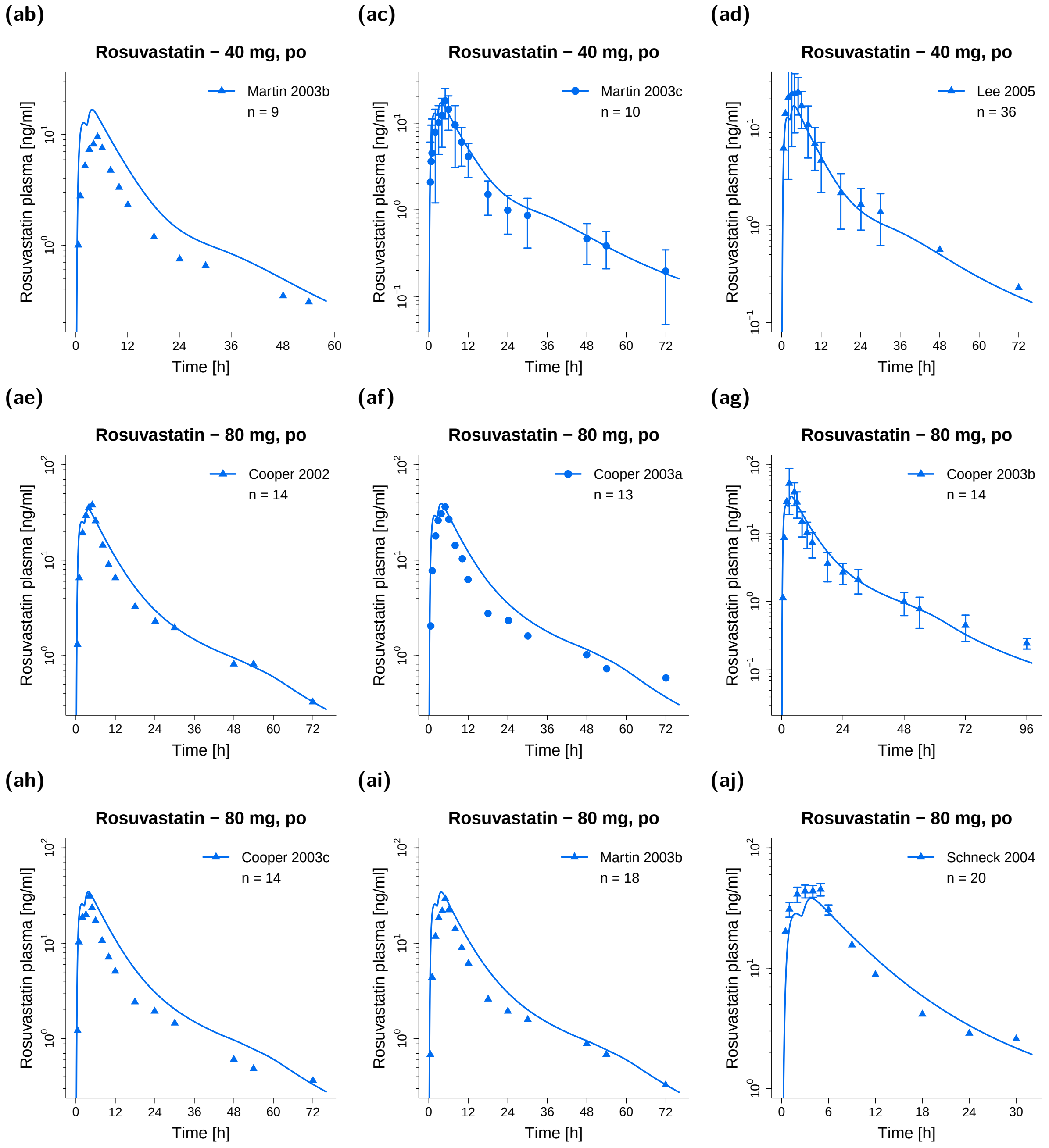

Figure S3.4.1: Rosuvastatin plasma concentration-time profiles (semilogarithmic). Simulations are shown as lines, observed data are shown as dots (training dataset) or triangles (test dataset) ± SD, if available. Details on administration protocols, study populations and literature references are listed in Table S3.2.1 (continued)

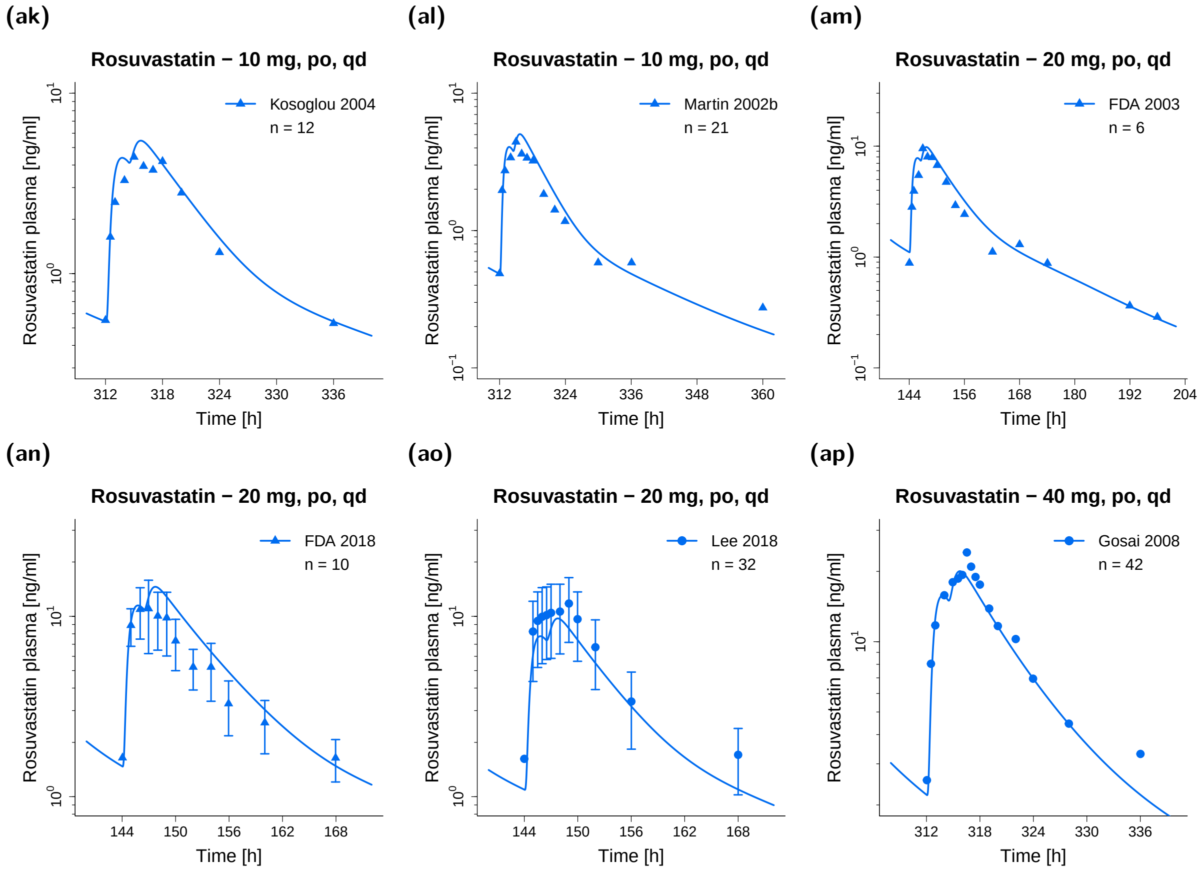

Figure S3.4.1: Rosuvastatin plasma concentration-time profiles (semilogarithmic). Simulations are shown as lines, observed data are shown as dots (training dataset) or triangles (test dataset) ± SD, if available. Details on administration protocols, study populations and literature references are listed in Table S3.2.1 (continued)

3.4.2 Linear plots - Plasma

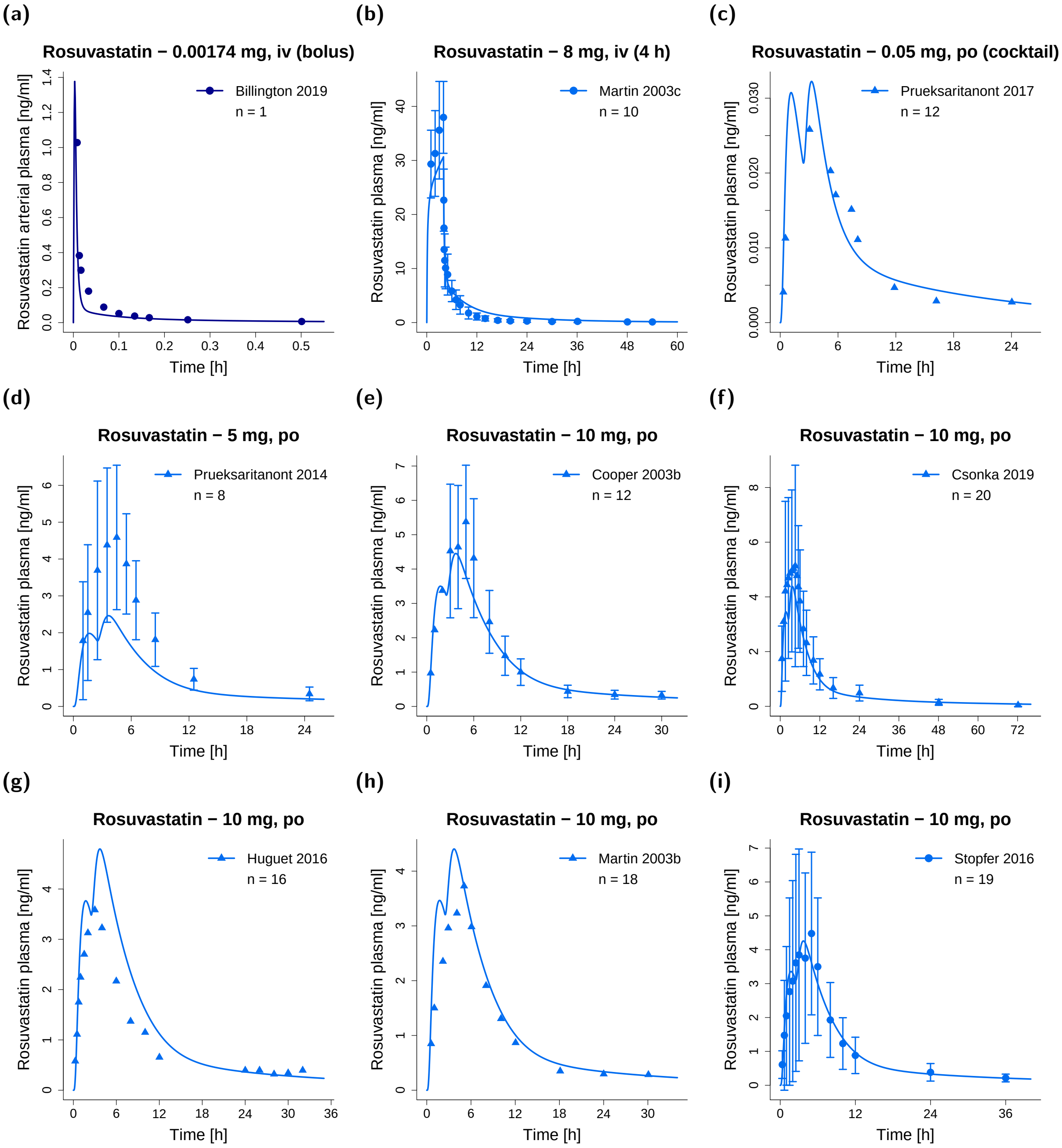

Figure S3.4.2: Rosuvastatin plasma concentration-time profiles (linear). Simulations are shown as lines, observed data are shown as dots (training dataset) or triangles (test dataset) ± SD, if available. Details on administration protocols, study populations and literature references are listed in Table S3.2.1

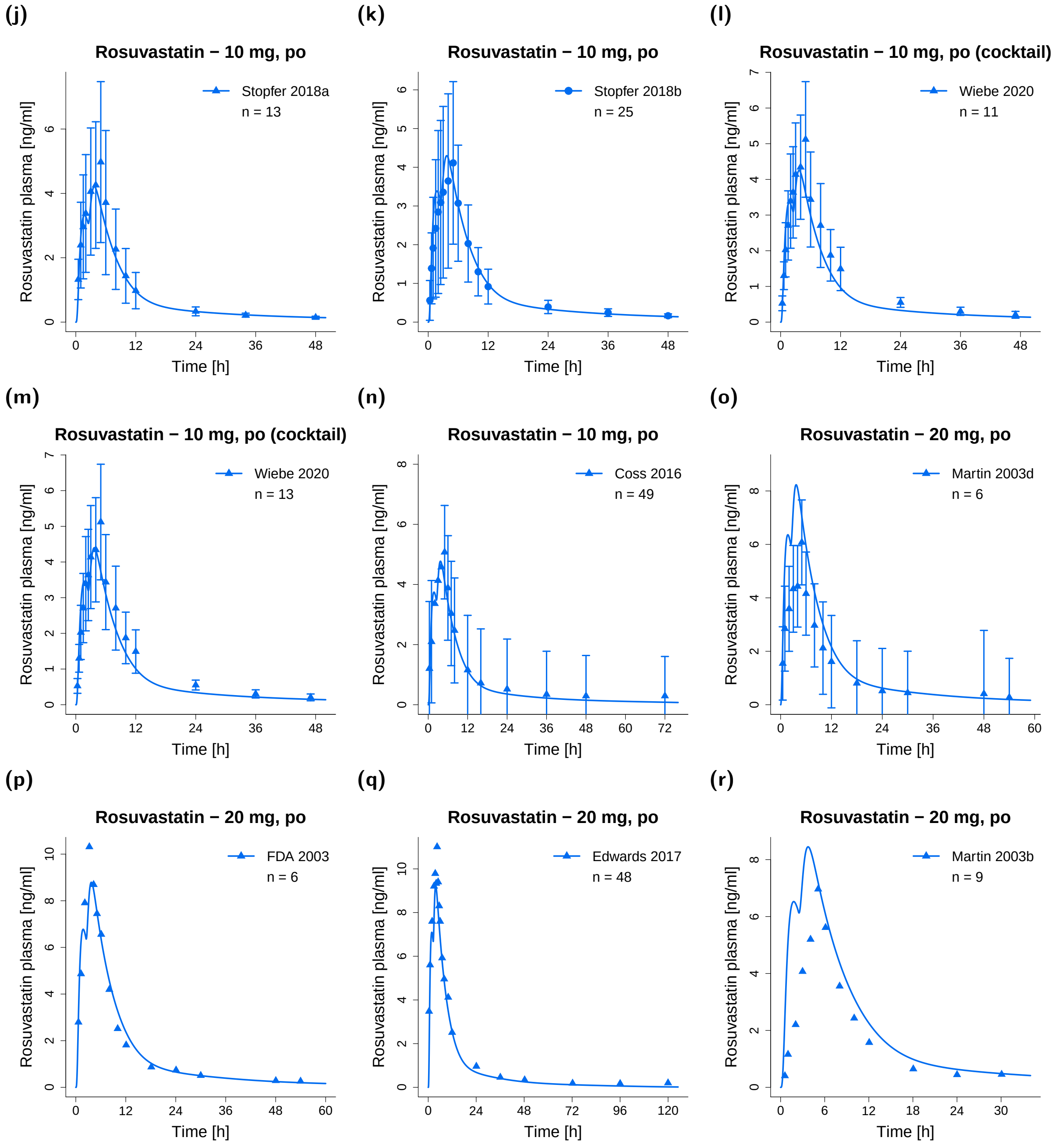

Figure S3.4.2: Rosuvastatin plasma concentration-time profiles (linear). Simulations are shown as lines, observed data are shown as dots (training dataset) or triangles (test dataset) ± SD, if available. Details on administration protocols, study populations and literature references are listed in Table S3.2.1 (continued)

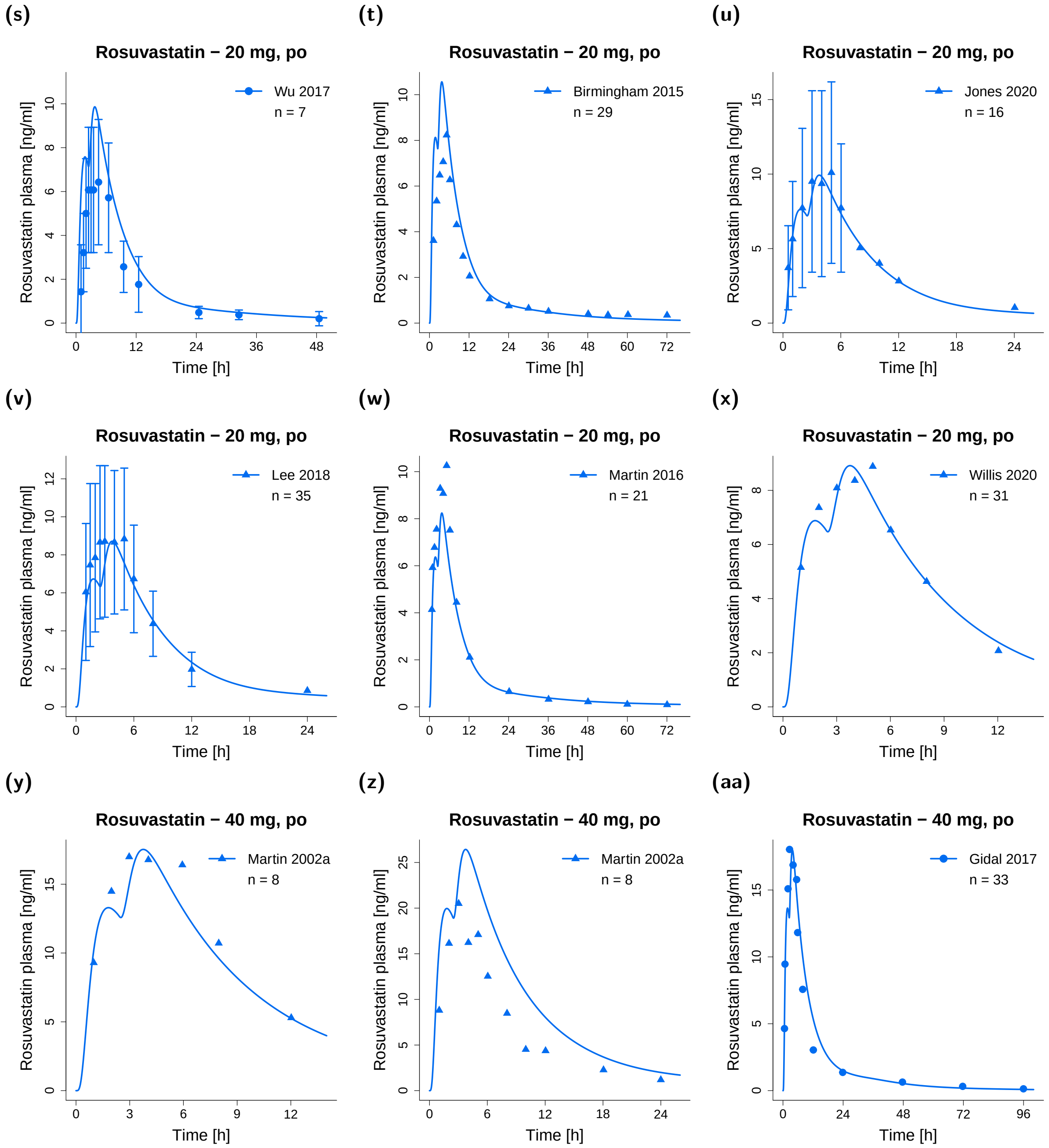

Figure S3.4.2: Rosuvastatin plasma concentration-time profiles (linear). Simulations are shown as lines, observed data are shown as dots (training dataset) or triangles (test dataset) ± SD, if available. Details on administration protocols, study populations and literature references are listed in Table S3.2.1 (continued)

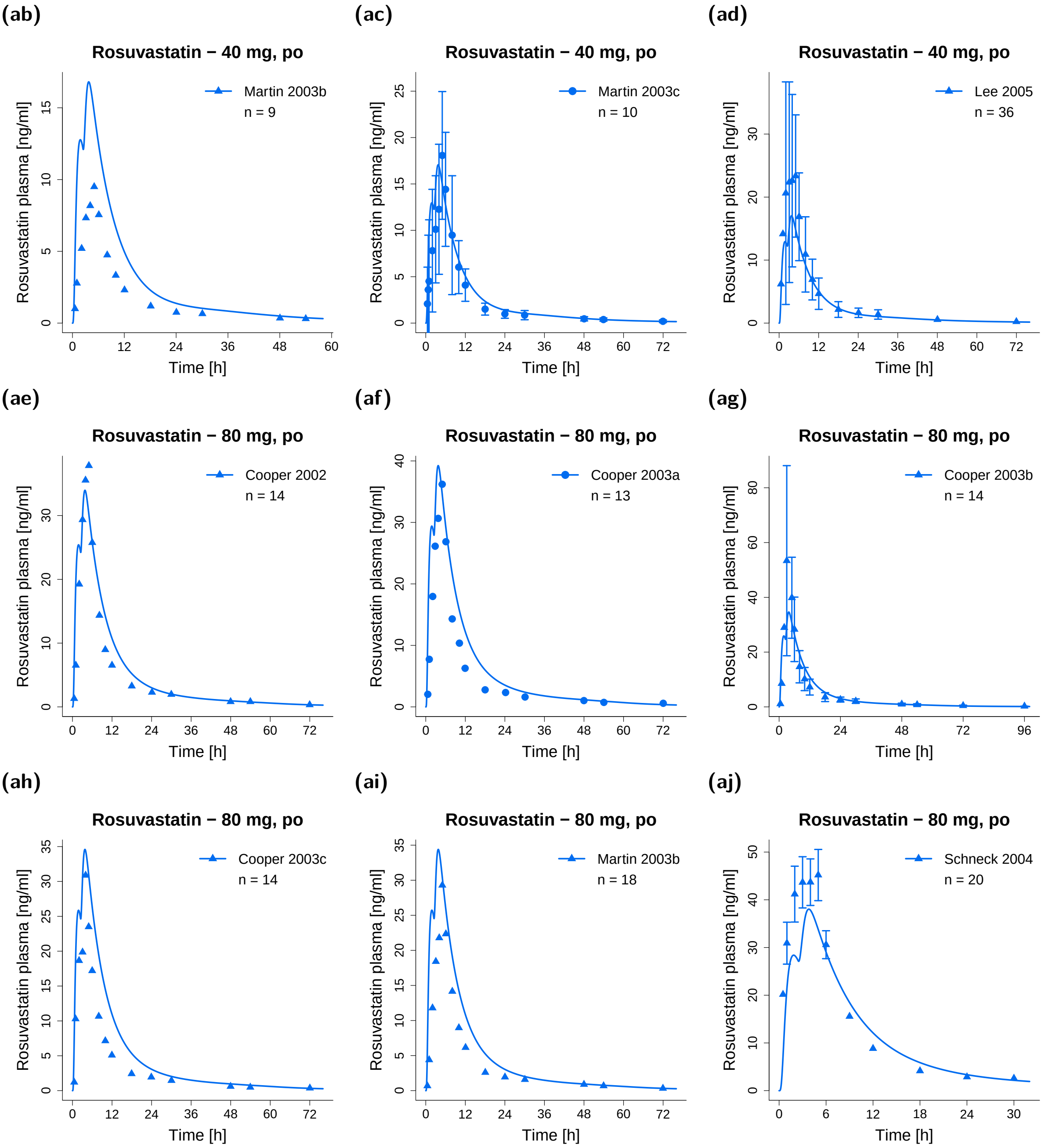

Figure S3.4.2: Rosuvastatin plasma concentration-time profiles (linear). Simulations are shown as lines, observed data are shown as dots (training dataset) or triangles (test dataset) ± SD, if available. Details on administration protocols, study populations and literature references are listed in Table S3.2.1 (continued)

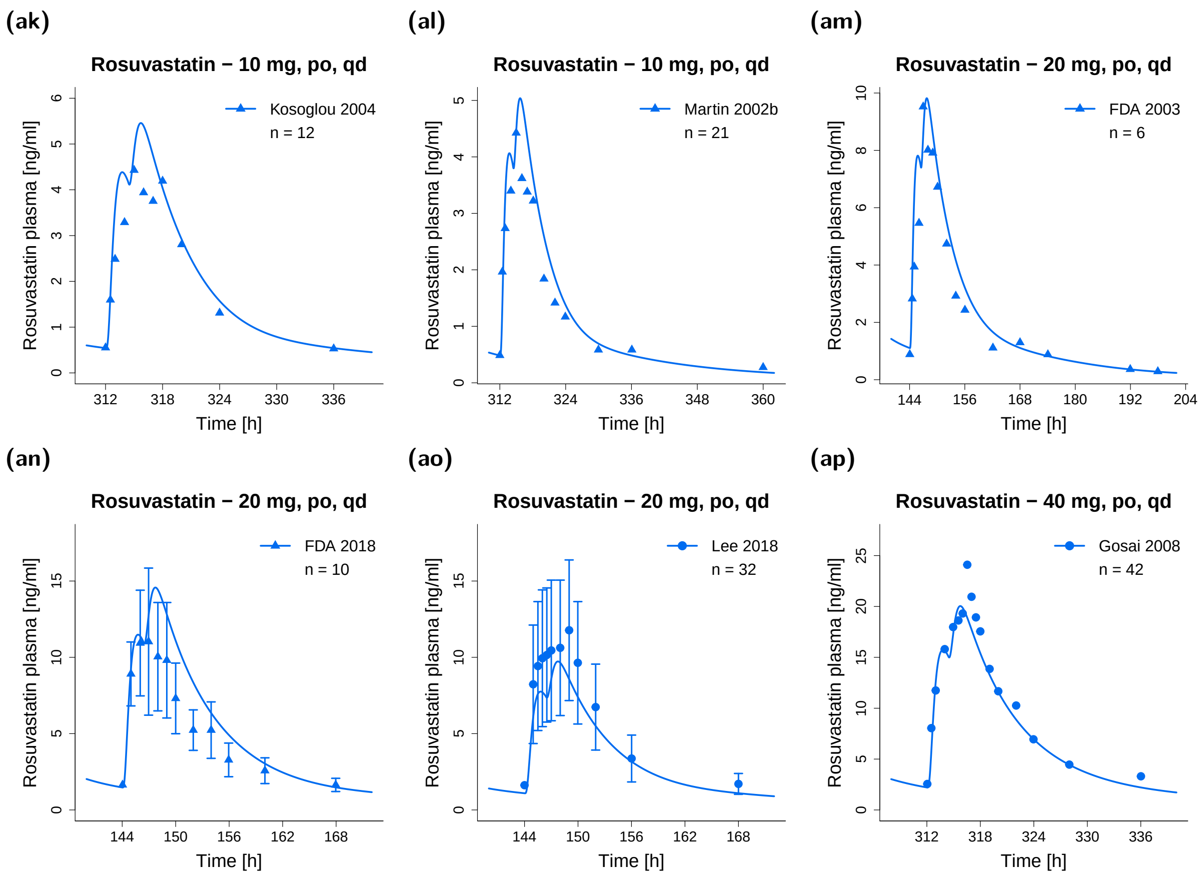

Figure S3.4.2: Rosuvastatin plasma concentration-time profiles (linear). Simulations are shown as lines, observed data are shown as dots (training dataset) or triangles (test dataset) ± SD, if available. Details on administration protocols, study populations and literature references are listed in Table S3.2.1 (continued)

3.4.3 Semilogarithmic plots – PET study

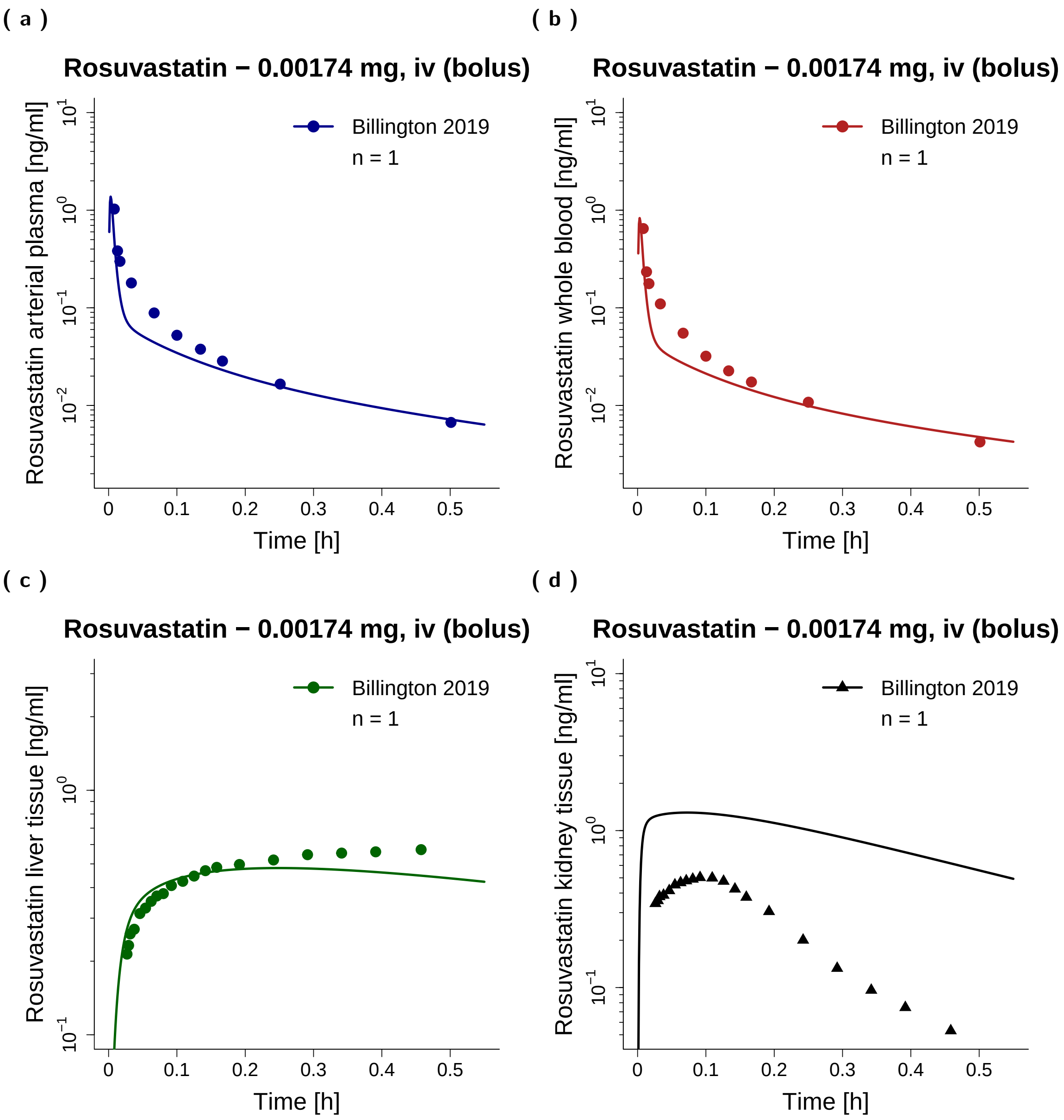

Figure S3.4.3: Rosuvastatin microdose PET study predictions (semilogarithmic). Simulations are shown as lines, observed data are shown as dots (training dataset) or triangles (test dataset) [9]. The gallbladder concentrations measured in this study are presented as fraction of dose excreted in bile in Figure S3.4.5

3.4.4 Linear plots – PET study

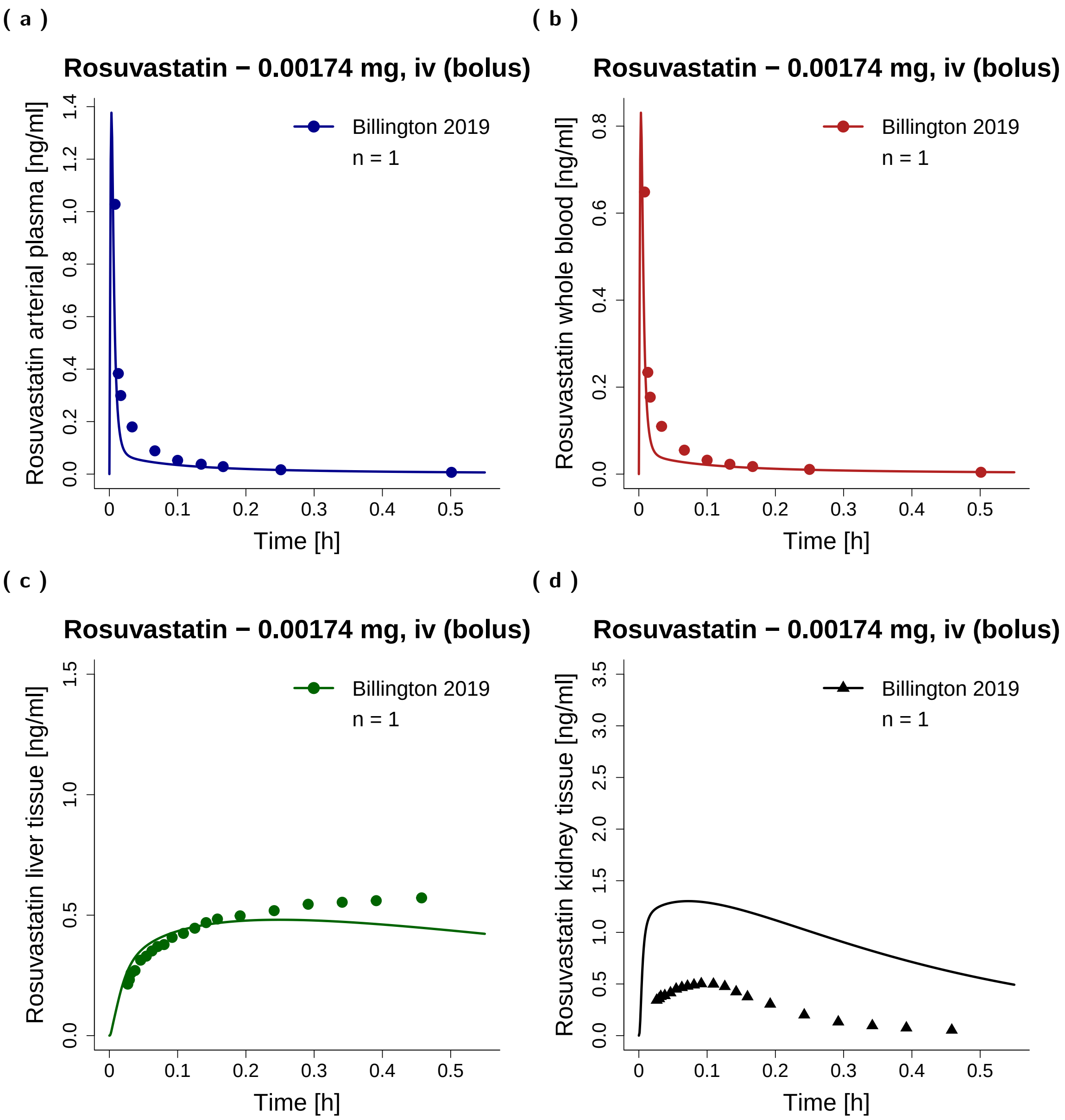

Figure S3.4.4: Rosuvastatin microdose PET study predictions (linear). Simulations are shown as lines, observed data are shown as dots (training dataset) or triangles (test dataset) [9]. The gallbladder concentrations measured in this study are presented as fraction of dose excreted in bile in Figure S3.4.5

3.4.5 Linear plots – Fraction excreted

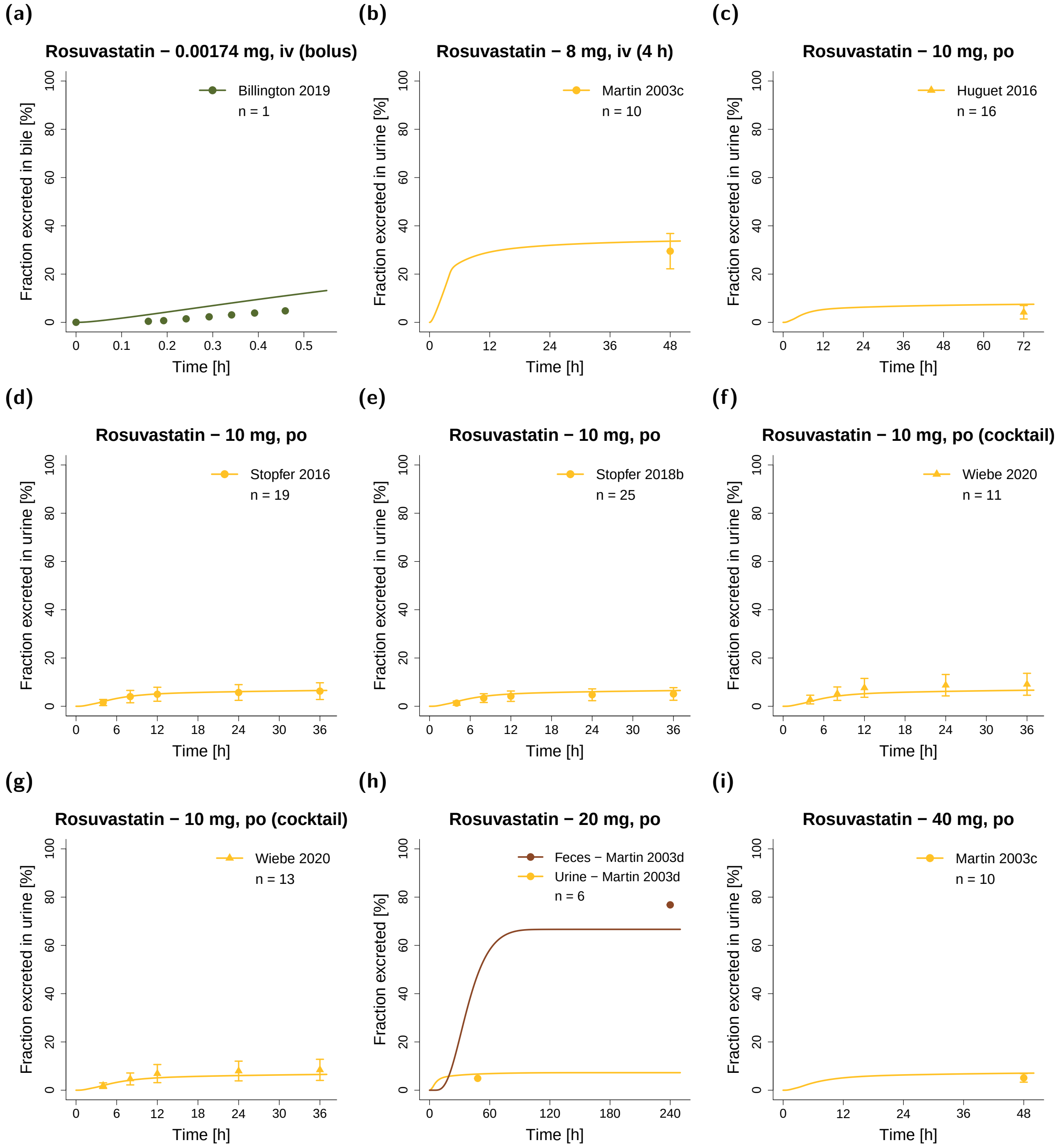

Figure S3.4.5: Rosuvastatin fractions excreted in urine, feces or bile. Simulations are shown as lines, observed data are shown as dots (training dataset) or triangles (test dataset) ± SD, if available. Details on administration protocols, study populations and literature references are listed in Table S3.2.1

3.5 Model evaluation

3.5.1 Plasma concentration goodness-of-fit plots

(a) Training

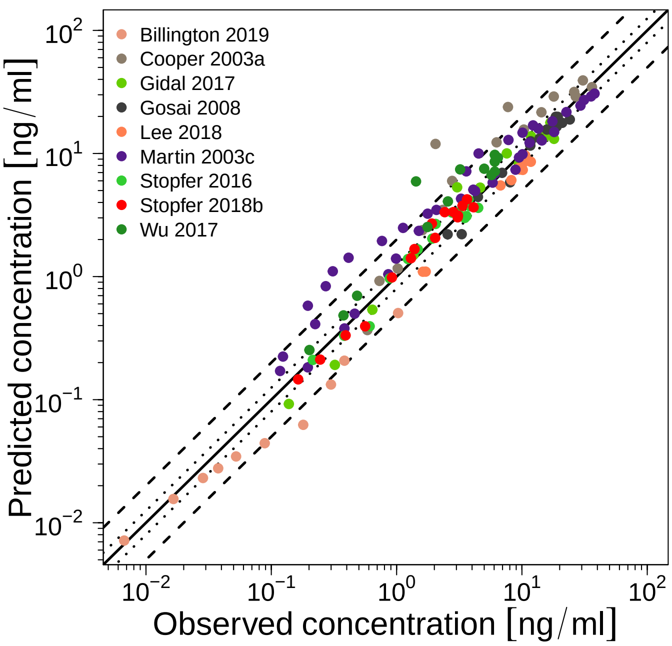

(b) Test

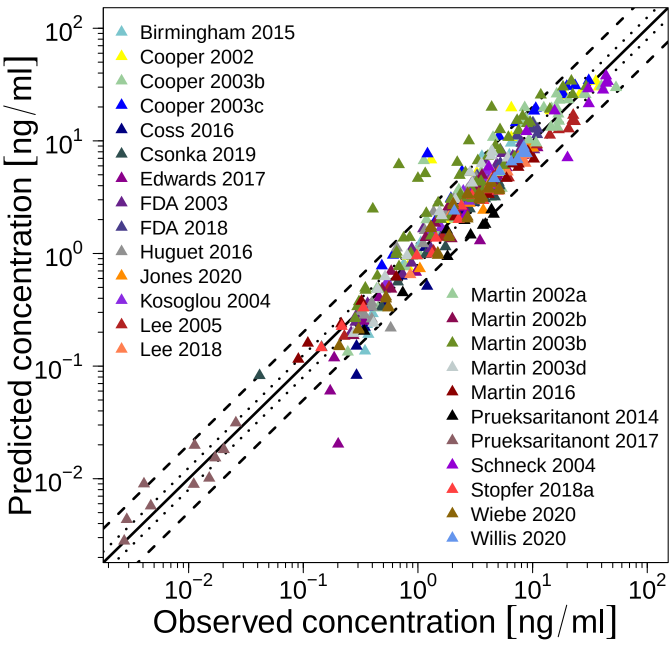

Figure S3.5.1: Predicted versus observed rosuvastatin plasma concentrations of (a) the training and (b) the test dataset. Solid lines (——) mark the line of identity. Dotted lines (······) indicate 1.25-fold, dashed lines (- -) indicate 2-fold deviation. Details on administration protocols, study populations and literature references are listed in Table S3.2.1

3.5.2 Mean relative deviation of plasma concentration predictions

Table S3.5.1: Mean relative deviation (MRD) of rosuvastatin plasma concentration predictions

Route	Dose	MRD	Reference
iv, bolus	0.00174 [a]	1.80	Billington et al. 2019 [9]
iv, 4 h	8.0	1.84	Martin et al. 2003c [4]
po, sol	0.05 [b]	1.46	Prueksaritanont et al. 2017 [10]
po, -	5.0	1.80	Prueksaritanont et al. 2014 [11]
po, caps	10.0	1.18	Cooper et al. 2003b [12]
po, tab	10.0	1.32	Csonka et al. 2019 [13]
po, tab	10.0	1.47	Huguet et al. 2016 [14]
po, tab	10.0	1.33	Martin et al. 2003b [15]
po, tab	10.0	1.19	Stopfer et al. 2016 [5]
po, tab	10.0	1.16	Stopfer et al. 2018a [16]
po, tab	10.0	1.20	Stopfer et al. 2018b [6]
po, tab	10.0 [c]	1.33	Wiebe et al. 2020 [7]
po, tab	10.0 [c]	1.31	Wiebe et al. 2020 [7]
po, -	10.0	1.60	Coss et al. 2016 [17]
po, sol	20.0	1.50	Martin et al. 2003d [18]
po, caps	20.0	1.18	FDA et al. 2003 [19]
po, tab	20.0	1.88	Edwards et al. 2017 [20]
po, tab	20.0	2.17	Martin et al. 2003b [15]
po, tab	20.0	1.74	Wu et al. 2017 [21]
po, -	20.0	1.50	Birmingham et al. 2015 [22]
po, -	20.0	1.19	Jones et al. 2020 [23]
po, -	20.0	1.18	Lee et al. 2018 [24]
po, -	20.0	1.23	Martin et al. 2016 [25]
po, -	20.0	1.08	Willis et al. 2020 [26]
po, caps	40.0	1.12	Martin et al. 2002a [27]
po, caps	40.0	1.66	Martin et al. 2002a [27]
po, tab	40.0	1.33	Gidal et al. 2017 [28]
po, tab	40.0	2.16	Martin et al. 2003b [15]
po, tab	40.0	1.45	Martin et al. 2003c [4]
po, -	40.0	1.31	Lee et al. 2005 [29]
po, tab	80.0	1.74	Cooper et al. 2002 [30]
po, tab	80.0	1.91	Cooper et al. 2003a [31]
po, tab	80.0	1.78	Cooper et al. 2003b [12]
po, tab	80.0	1.95	Cooper et al. 2003c [32]
po, tab	80.0	2.17	Martin et al. 2003b [15]
po, tab	80.0	1.48	Schneck et al. 2004 [8]
po, -, qd	10.0	1.21	Kosoglou et al. 2004 [33]
po, -, qd	10.0	1.27	Martin et al. 2002b [34]
po, caps, qd	20.0	1.28	FDA et al. 2003 [19]
po, -, qd	20.0	1.31	FDA et al. 2018 [35]
po, -, qd	20.0	1.32	Lee et al. 2018 [24]
po, -, qd	40.0	1.18	Gosai et al. 2008 [36]
**Overall MRD**		**1.48 (1.08–2.17)**	
		**39/42 with MRD ≤ 2**	

[a] 160 minutes earlier a 5 mg rosuvastatin oral dose was given, to ensure pharmacokinetics comparable to clinical doses, [b] administered as microdose cocktail together with 0.01 mg midazolam, 0.375 mg dabigatran etexilate, 0.01 mg pitavastatin and 0.1 mg atorvastatin, [c] administered as cocktail together with 0.25 mg digoxin, 1 mg furosemide and 10 mg metformin, **-**: not given, **caps**: capsule, **iv**: intravenous, **po**: oral, **qd**: once daily, **sol**: solution, **tab**: tablet

3.5.3 AUC_{last} and C_{max} goodness-of-fit plots

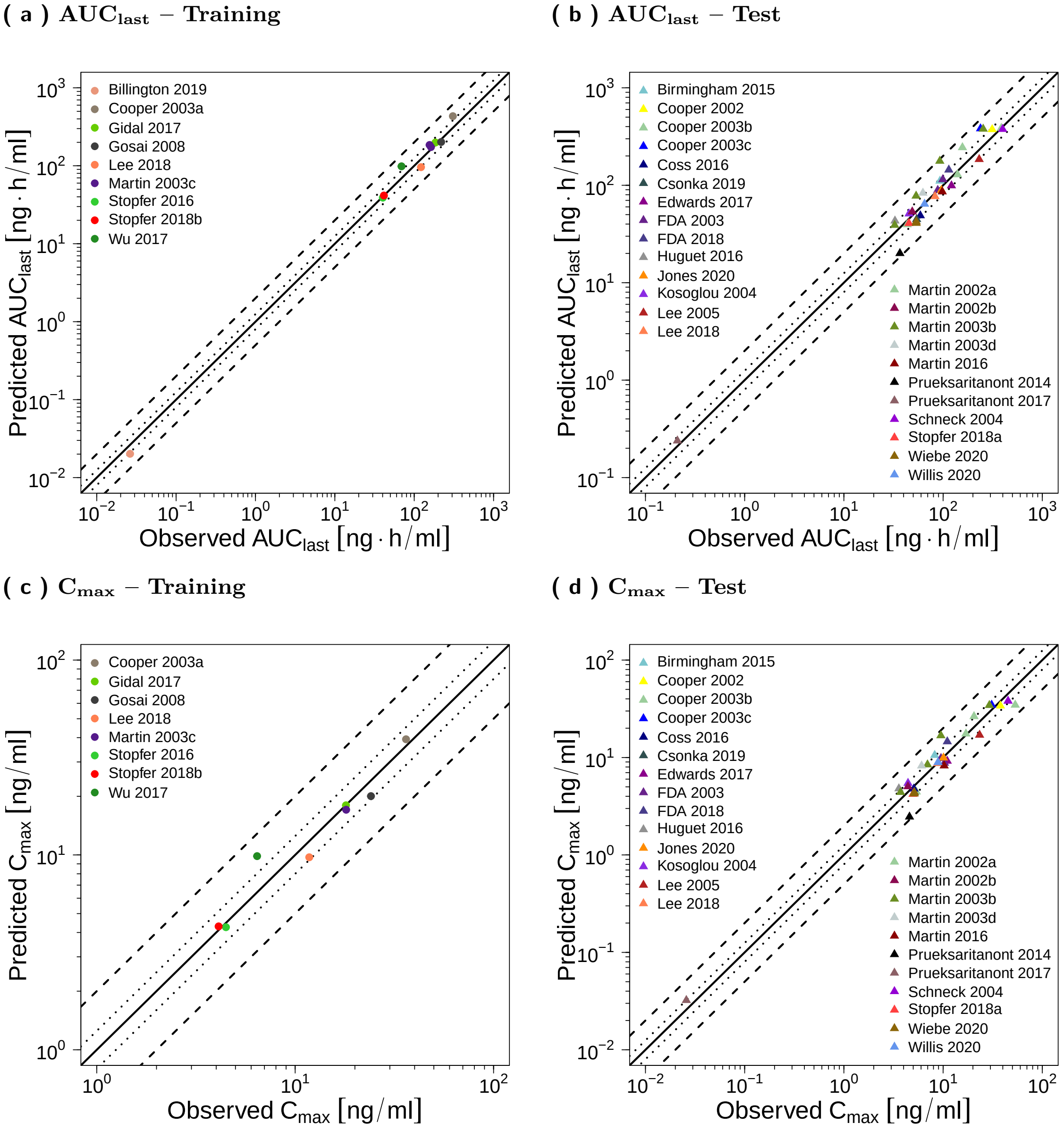

Figure S3.5.2: Predicted versus observed AUC_{last} and C_{max} values for (a,c) the training and (b,d) the test dataset. Solid lines (——) mark the line of identity. Dotted lines (·····) indicate 1.25-fold, dashed lines (- -) indicate 2-fold deviation. Details on administration protocols, study populations and literature references are listed in Table S3.2.1

3.5.4 Geometric mean fold error of predicted AUC_{last} and C_{max} values

Table S3.5.2: Predicted and observed rosuvastatin AUC_{last} and C_{max} values with geometric mean fold errors (GMFEs)

		AUC_{last}			C_{max}			
Route	Dose	Pred [ng·h/ml]	Obs [ng·h/ml]	GMFE	Pred [ng/ml]	Obs [ng/ml]	GMFE	Reference
**Intravenous**								
iv, bolus	0.00174 [a]	0.02	0.03	1.34	-	-	-	Billington et al. 2019 [9]
iv, 4 h	8.0	173.93	163.14	1.07	-	-	-	Martin et al. 2003c [4]
**Oral**								
po, sol	0.05 [b]	0.24	0.21	1.12	0.03	0.03	1.25	Prueksaritanont et al. 2017 [10]
po, -	5.0	20.03	37.46	1.87	2.46	4.59	1.86	Prueksaritanont et al. 2014 [11]
po, caps	10.0	39.55	44.07	1.11	4.45	5.37	1.21	Cooper et al. 2003b [12]
po, tab	10.0	44.76	55.45	1.24	4.38	5.13	1.17	Csonka et al. 2019 [13]
po, tab	10.0	43.19	33.05	1.31	4.80	3.58	1.34	Huguet et al. 2016 [14]
po, tab	10.0	39.00	32.92	1.18	4.40	3.73	1.18	Martin et al. 2003b [15]
po, tab	10.0	39.02	41.00	1.05	4.26	4.48	1.05	Stopfer et al. 2016 [5]
po, tab	10.0	40.71	46.26	1.14	4.21	4.97	1.18	Stopfer et al. 2018a [16]
po, tab	10.0	41.56	42.22	1.02	4.30	4.11	1.05	Stopfer et al. 2018b [6]
po, tab	10.0 [c]	40.87	54.88	1.34	4.25	5.12	1.20	Wiebe et al. 2020 [7]
po, tab	10.0 [c]	42.33	54.88	1.30	4.40	5.12	1.16	Wiebe et al. 2020 [7]
po, -	10.0	48.90	59.54	1.22	4.77	5.07	1.06	Coss et al. 2016 [17]
po, sol	20.0	83.38	63.22	1.32	8.23	6.07	1.35	Martin et al. 2003d [18]
po, caps	20.0	89.01	89.29	1.00	8.79	10.31	1.17	FDA et al. 2003 [19]
po, tab	20.0	99.28	123.77	1.25	9.21	11.01	1.19	Edwards et al. 2017 [20]
po, tab	20.0	77.78	54.22	1.43	8.45	6.96	1.21	Martin et al. 2003b [15]
po, tab	20.0	98.47	71.63	1.37	9.86	6.43	1.53	Wu et al. 2017 [21]
po, -	20.0	110.76	93.77	1.18	10.56	8.23	1.28	Birmingham et al. 2015 [22]
po, -	20.0	89.00	98.51	1.11	9.93	10.10	1.02	Jones et al. 2020 [23]
po, -	20.0	76.91	84.63	1.10	8.71	8.83	1.01	Lee et al. 2018 [24]
po, -	20.0	86.00	101.02	1.17	8.24	10.26	1.24	Martin et al. 2016 [25]
po, -	20.0	64.31	65.90	1.02	8.91	8.88	1.00	Willis et al. 2020 [26]
po, caps	40.0	128.76	141.49	1.10	17.53	16.99	1.03	Martin et al. 2002a [27]
po, caps	40.0	244.39	158.63	1.54	26.43	20.50	1.29	Martin et al. 2002a [27]
po, tab	40.0	198.83	190.05	1.05	17.98	18.03	1.00	Gidal et al. 2017 [28]

[a] 160 minutes earlier a 5 mg rosuvastatin oral dose was given, to ensure pharmacokinetics comparable to clinical doses, [b] administered as microdose cocktail together with 0.01 mg midazolam, 0.375 mg dabigatran etexilate, 0.01 mg pitavastatin and 0.1 mg atorvastatin, [c] administered as cocktail together with 0.25 mg digoxin, 1 mg furosemide and 10 mg metformin, **-**: not given/not calculated, **caps**: capsule, **iv**: intravenous, **obs**: observed, **po**: oral, **pred**: predicted, **qd**: once daily, **sol**: solution, **tab**: tablet

Table S3.5.2: Predicted and observed rosuvastatin AUC_{last} and C_{max} values with geometric mean fold errors (GMFEs) *(continued)*

		AUC_{last}			C_{max}			
Route	Dose	Pred [ng·h/ml]	Obs [ng·h/ml]	GMFE	Pred [ng/ml]	Obs [ng/ml]	GMFE	Reference
po, tab	40.0	177.32	93.84	1.89	16.81	9.50	1.77	Martin et al. 2003b [15]
po, tab	40.0	184.96	158.94	1.16	17.06	18.07	1.06	Martin et al. 2003c [4]
po, -	40.0	184.74	234.87	1.27	17.02	23.35	1.37	Lee et al. 2005 [29]
po, tab	80.0	374.37	318.12	1.18	33.96	37.83	1.11	Cooper et al. 2002 [30]
po, tab	80.0	433.12	311.14	1.39	39.23	36.20	1.08	Cooper et al. 2003a [31]
po, tab	80.0	385.95	392.78	1.02	34.62	53.37	1.54	Cooper et al. 2003b [12]
po, tab	80.0	381.66	241.93	1.58	34.59	30.88	1.12	Cooper et al. 2003c [32]
po, tab	80.0	378.64	257.68	1.47	34.39	29.29	1.17	Martin et al. 2003b [15]
po, tab	80.0	376.34	404.47	1.07	38.04	45.19	1.19	Schneck et al. 2004 [8]
po, -, qd	10.0	51.07	46.58	1.10	5.46	4.43	1.23	Kosoglou et al. 2004 [33]
po, -, qd	10.0	53.56	49.73	1.08	5.04	4.42	1.14	Martin et al. 2002b [34]
po, caps, qd	20.0	114.78	100.98	1.14	9.82	9.52	1.03	FDA et al. 2003 [19]
po, -, qd	20.0	144.03	114.91	1.25	14.57	11.03	1.32	FDA et al. 2018 [35]
po, -, qd	20.0	96.02	123.90	1.29	9.73	11.77	1.21	Lee et al. 2018 [24]
po, -, qd	40.0	201.94	219.39	1.09	20.04	24.10	1.20	Gosai et al. 2008 [36]
**Overall GMFE**				**1.24 (1.00–1.89)**			**1.22 (1.00–1.86)**	
				**42/42 with GMFE ≤ 2**			**40/40 with GMFE ≤ 2**	

[a] 160 minutes earlier a 5 mg rosuvastatin oral dose was given, to ensure pharmacokinetics comparable to clinical doses, [b] administered as microdose cocktail together with 0.01 mg midazolam, 0.375 mg dabigatran etexilate, 0.01 mg pitavastatin and 0.1 mg atorvastatin, [c] administered as cocktail together with 0.25 mg digoxin, 1 mg furosemide and 10 mg metformin, **-**: not given/not calculated, **caps**: capsule, **iv**: intravenous, **obs**: observed, **po**: oral, **pred**: predicted, **qd**: once daily, **sol**: solution, **tab**: tablet

3.5.5 Sensitivity analysis

Sensitivity of the rosuvastatin model to single parameter values (local sensitivity analysis) was calculated as the relative change of the predicted $AUC_{0\text{-}24}$ (Figure S3.5.3) of a 40 mg single dose of rosuvastatin administered as tablet in the fasted state (highest recommended dose). Sensitivity analysis was carried out using a relative parameter perturbation of 1000 % (variation range 10.0, maximum number of 9 steps). Parameters were included into the analysis if they were optimized (luminal intestinal permeability, basolateral intestinal permeability, OATP2B1 kcat, OATP1B1/1B3 kcat, OAT3 kcat, Pgp kcat, BCRP kcat, CYP2C9 CLspec), if they are associated with optimized parameters (OATP2B1 Km, OATP1B1/1B3 Km, OAT3 Km, Pgp Km, BCRP Km), or if they might have a strong impact due to calculation methods used in the model (solubility, lipophilicity, fraction unbound, blood/plasma concentration ratio, GFR fraction).

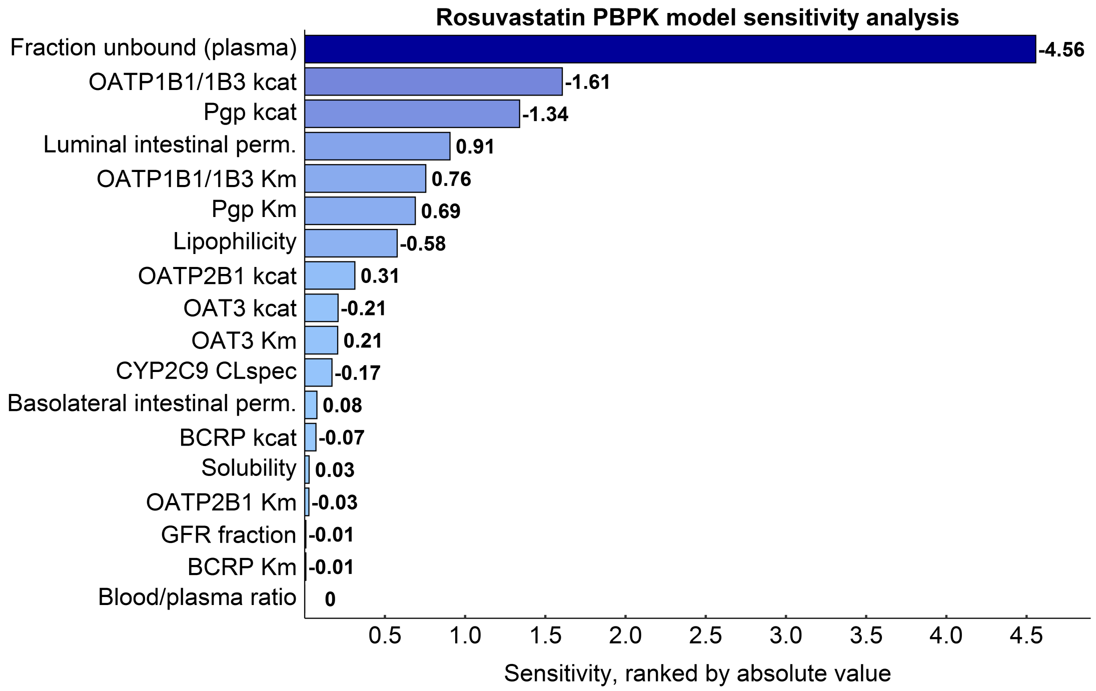

Figure S3.5.3: Rosuvastatin PBPK model sensitivity analysis. Sensitivity of the model to single parameters, calculated as change of the predicted $AUC_{0\text{-}24}$ of a 40 mg single oral dose. For the basolateral intestinal permeability, only the highest value of all 11 different intestinal segments is shown. **GFR**: glomerular filtration rate, **kcat**: transport or metabolic rate constant (turnover number), **Km**: Michaelis-Menten constant, **perm.**: permeability

4 Rifampicin-rosuvastatin drug-drug interaction (DDI)

4.1 DDI modeling

The rifampicin-rosuvastatin DDI was modeled using a previously established whole-body PBPK model of rifampicin [47]. The drug-dependent parameters of this model are reproduced in Table S4.2.1.

The rifampicin-rosuvastatin interaction was simulated as competitive inhibition of OATP2B1, Pgp, BCRP, OATP1B1/1B3 and CYP2C9 by rifampicin. The parameters to model these inhibitions were obtained from literature [11, 48–50] or in-house measurements (rifampicin Pgp IC_{50}), and were included in the rifampicin drug-dependent parameter Table S4.2.1. To account for the impact of rifampicin on the absorption of rosuvastatin, the rosuvastatin dose during the rifampicin-rosuvastatin DDI was modeled as a single dose without a lag time, as indicated by the PopPK analysis.

Details on the predicted clinical DDI studies are given in Table S4.3.1. Model predictions of rosuvastatin plasma concentration-time profiles before and during rifampicin co-administration, compared to observed data, are shown in Figures S4.4.1 and S4.4.2. Predicted compared to observed rosuvastatin fraction excreted in urine before and during rifampicin co-administration are shown in Figure S4.4.3. The correlation of predicted to observed DDI AUC_{last} ratios and DDI C_{max} ratios is shown in Figure S4.5.1. Table S4.5.1 lists the corresponding predicted and observed DDI AUC_{last} ratios, DDI C_{max} ratios, as well as GMFE values.

4.2 Rifampicin drug-dependent parameters

The drug-dependent parameters of the rifampicin model are summarized in Table S4.2.1. The associated system-dependent parameters are listed in Table S7.0.1.

Table S4.2.1: Drug-dependent parameters of the rifampicin PBPK model (adopted from [47])

Parameter	Model	Unit	Source	Literature	Reference	Description
MW	822.94	g/mol	Literature	822.94	[37]	Molecular weight
pKa (acid)	1.70	-	Literature	1.70	[51]	First acid dissociation constant
pKa (base)	7.90	-	Literature	7.90	[51]	Second acid dissociation constant
Solubility (pH 7.5)	2.80	g/l	Literature	2.80	[52]	Solubility
logP	2.50	-	Optimized	1.30, 2.70	[37, 53]	Lipophilicity
fu	17.00	%	Literature	17.00	[54]	Fraction unbound in plasma
B/P ratio	0.89	-	Calculated	0.90 [a]	[55]	Blood/plasma ratio
OATP1B1 K_m	1.50	µmol/l	Literature	1.50	[56]	OATP1B1 Michaelis-Menten constant
OATP1B1 k_{cat}	105.35 [b]	1/min	Optimized	-	-	OATP1B1 transport rate constant
AADAC K_m	195.10	µmol/l	Literature	195.10	[57]	AADAC Michaelis-Menten constant
AADAC k_{cat}	9.87	1/min	Optimized	-	-	AADAC catalytic rate constant
Pgp K_m	55.00	µmol/l	Literature	55.00	[58]	Pgp Michaelis-Menten constant
Pgp k_{cat}	11.12 [b]	1/min	Optimized	-	-	Pgp transport rate constant
GFR fraction	1.00	-	Assumed	-	-	Fraction of filtered drug in the urine
EHC continuous fraction	1.00	-	Assumed	-	-	Fraction of bile continually released
Induction EC_{50}	0.34	µmol/l	Literature	0.80*0.42 [c]	[54, 59]	Conc. for half-maximal induction
E_{max} OATP1B1	0.38	-	Optimized	-	-	Maximum in vivo induction effect
E_{max} AADAC	0.99	-	Optimized	-	-	Maximum in vivo induction effect
E_{max} Pgp	2.50	-	Literature	2.50	[60]	Maximum in vivo induction effect
OATP2B1 K_i	78.20	µmol/l	Literature	78.20	[48]	Conc. for half-maximal inhibition
Pgp K_i	9.10	µmol/l	Measurement	9.10 [d]	in-house	Conc. for half-maximal inhibition
BCRP K_i	14.00	µmol/l	Literature	14.00	[11]	Conc. for half-maximal inhibition
OATP1B1 K_i	0.29	µmol/l	Literature	0.29	[49]	Conc. for half-maximal inhibition
CYP2C9 K_i	150.00	µmol/l	Literature	150.00 [e]	[50]	Conc. for half-maximal inhibition
Partition coefficients	Diverse	-	Calculated	R&R	[61, 62]	Cell to plasma partition coefficients
Cellular permeability	2.93E-05	cm/min	Calculated	PK-Sim	[2]	Permeability into the cellular space
Intestinal permeability	1.24E-05	cm/min	Optimized	3.84E-07	Calculated	Transcellular intestinal permeability

[a] in tuberculosis patients, [b] compared to [47], reference conc. was changed from assumed to literature value and k_{cat} was converted accordingly, [c] in vitro value corrected for binding in the assay, [d] new value, measured with 1 µmol/l digoxin in Caco-2 cells, [e] measured with 50 µmol/l tolbutamide in CYP2C9-expressing microsomes, **AADAC**: arylacetamide deacetylase, **BCRP**: breast cancer resistance protein, **conc.**: concentration, **CYP2C9**: cytochrome P450 2C9, **EHC**: enterohepatic circulation, **GFR**: glomerular filtration rate, **OATP1B1**: organic anion transporting polypeptide 1B1, **OATP2B1**: organic anion transporting polypeptide 2B1, **Pgp**: P-glycoprotein, **PK-Sim**: PK-Sim standard calculation method, **R&R**: Rodgers and Rowland calculation method

4.3 Rifampicin-rosuvastatin clinical DDI studies

The clinical studies used to evaluate the rifampicin-rosuvastatin DDI model performance are summarized in Table S4.3.1.

Table S4.3.1: Rifampicin-rosuvastatin DDI study table

**Perpetrator** Rifampicin	**Victim** Rosuvastatin	**Dose gap [h]**	**n**	**Male [%]**	**Age [years]**	**Weight [kg]**	**Height [cm]**	**BMI [kg/m^2]**	**Ethnicity**	**Dataset**	**Reference**
600 mg, iv, 0.5 h	5.0 mg, po, -	0.5	8	-	(19-55)	-	-	(19-32)	American	test	Prueksaritanont et al. 2014 [11]
600 mg, iv, 0.5 h	20.0 mg, po, tab	0.5	7	57	43 ± 14	68 ± 10	-	24 ± 2	White American	training	Wu et al. 2017 [21]
600 mg, po, caps	0.05 mg [a], po, sol	0	12/8	-	(19-55)	-	-	(19-32)	American	test	Prueksaritanont et al. 2017 [10]
600 mg, po, -	5.0 mg, po, -	0	8	-	(19-55)	-	-	(19-32)	American	test	Prueksaritanont et al. 2014 [11]
600 mg, po, tab	10.0 mg [b], po, tab	0	11	100	40 ± 12 (25-53)	86 ± 10 (72-104)	181 ± 6 (170-191)	26 ± 3 (22-29)	European	training	Wiebe et al. 2020 [7]

[a] administered as microdose cocktail together with 0.01 mg midazolam, 0.375 mg dabigatran etexilate, 0.01 mg pitavastatin and 0.1 mg atorvastatin, [b] administered as cocktail together with 0.25 mg digoxin, 1 mg furosemide and 10 mg metformin, **-**: not given, **BMI**: body mass index, **caps**: capsule, **iv**: intravenous, **n**: number of individuals studied, **po**: oral, **sol**: solution, **tab**: tablet, **test**: test dataset (model evaluation), **training**: training dataset (model development and parameter optimization)

4.4 Profiles

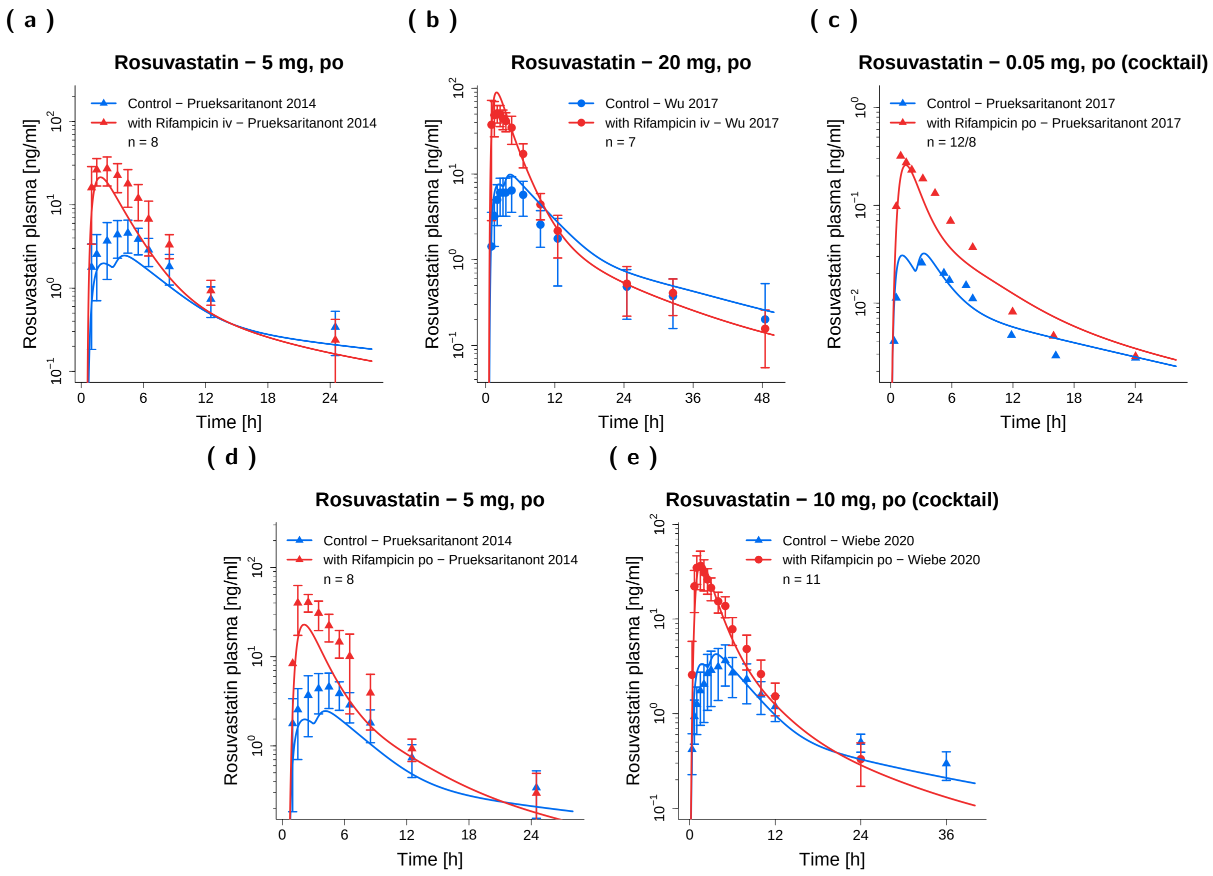

Figure S4.4.1: Rosuvastatin plasma concentration-time profiles (semilogarithmic), before and during rifampicin co-administration. Studies with intravenous rifampicin administration are shown in (a-b), studies with oral rifampicin administration are shown in (c-e). Simulations are shown as lines, observed data are shown as dots (training dataset) or triangles (test dataset) ± SD, if available. Details on administration protocols, study populations and literature references are listed in Table S4.3.1

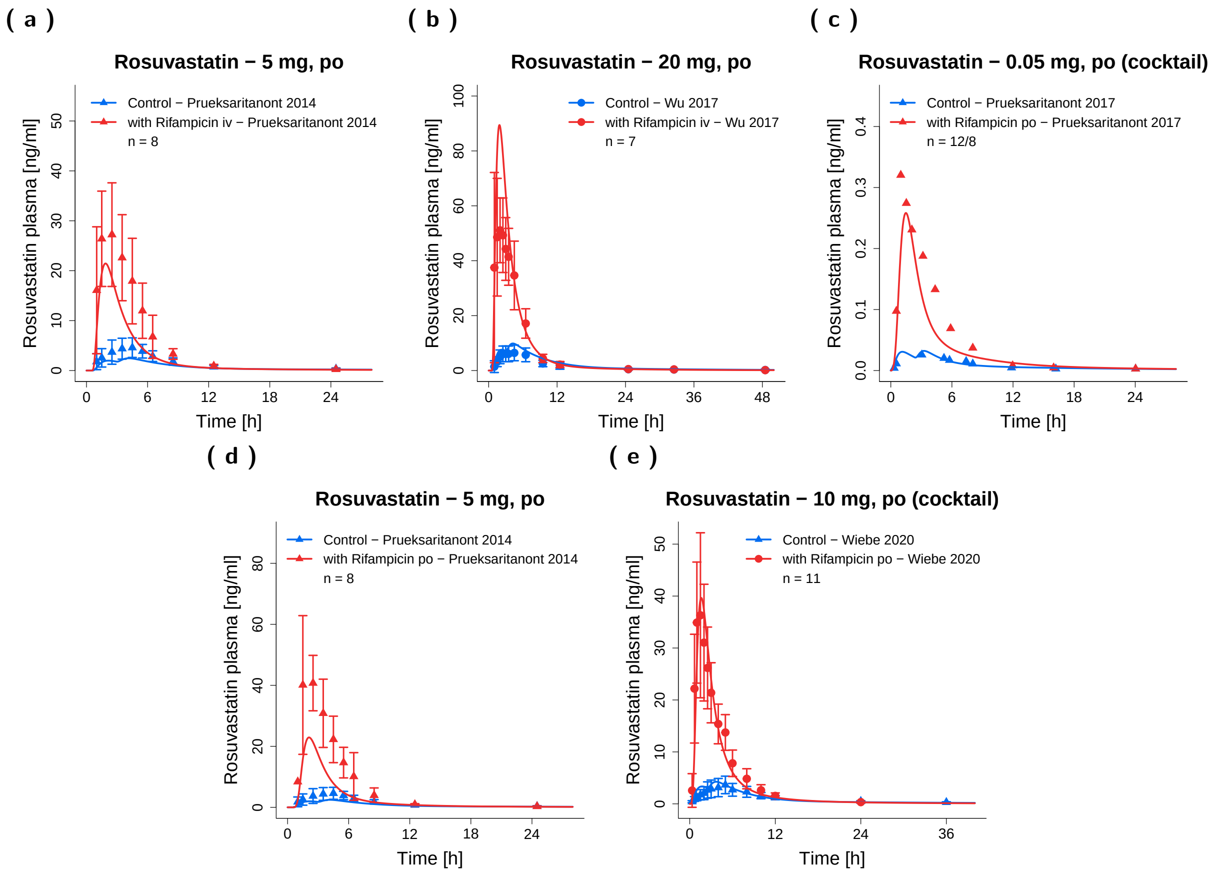

Figure S4.4.2: Rosuvastatin plasma concentration-time profiles (linear), before and during rifampicin co-administration. Studies with intravenous rifampicin administration are shown in (a-b), studies with oral rifampicin administration are shown in (c-e). Simulations are shown as lines, observed data are shown as dots (training dataset) or triangles (test dataset) ± SD, if available. Details on administration protocols, study populations and literature references are listed in Table S4.3.1

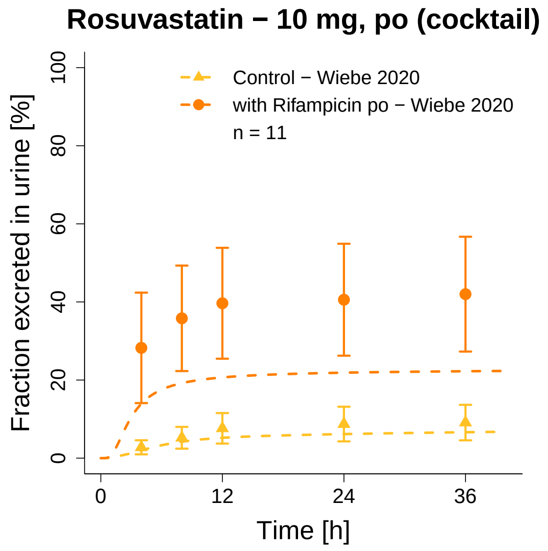

Figure S4.4.3: Rosuvastatin fraction excreted in urine profiles, before and during oral rifampicin co-administration. Simulations are shown as lines, observed data are shown as dots (training dataset) or triangles (test dataset) ± SD. Details on administration protocols, study population and literature reference are listed in Table S4.3.1

4.5 Model evaluation

4.5.1 DDI AUC_{last} and DDI C_{max} ratio goodness-of-fit plots

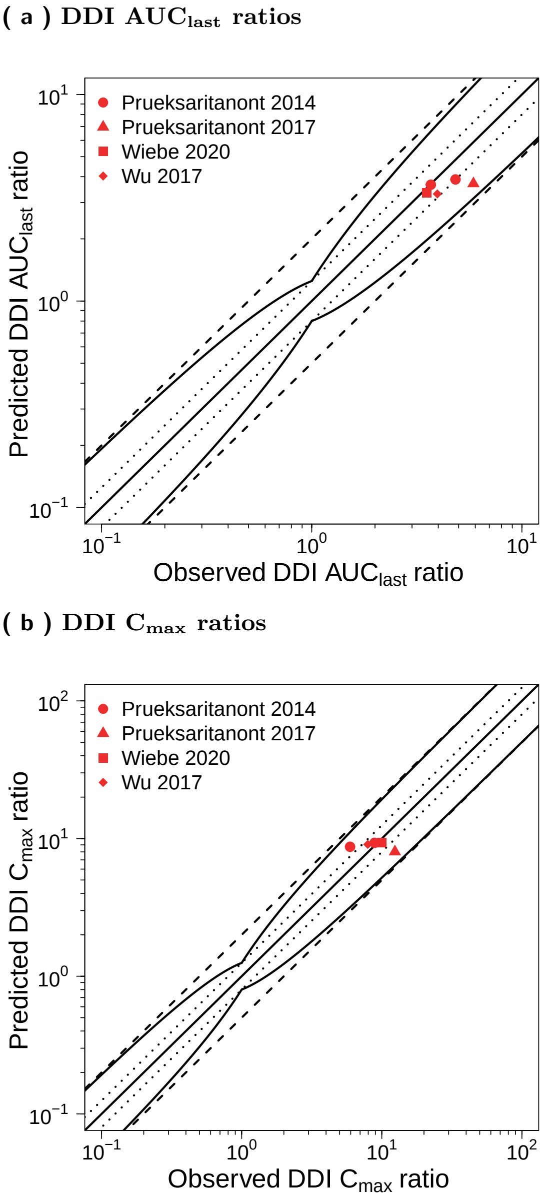

Figure S4.5.1: Predicted versus observed rifampicin-rosuvastatin DDI AUC_{last} ratios and DDI C_{max} ratios. The straight solid lines (—) mark the line of identity. Dotted lines (·····) indicate 1.25-fold, dashed lines (- -) indicate 2-fold deviation. The curved solid lines illustrate the DDI prediction success limits suggested by Guest et al. [63]. Details on administration protocols, study populations and literature references are listed in Table S4.3.1

4.5.2 Geometric mean fold error of predicted DDI AUC_{last} and DDI C_{max} ratios

Table S4.5.1: Predicted and observed rifampicin-rosuvastatin DDI AUC_{last} ratios and DDI C_{max} ratios with geometric mean fold errors (GMFEs)

**Perpetrator**	**Victim**			**DDI AUC_{last} ratio**			**DDI C_{max} ratio**			
Rifampicin	Rosuvastatin	Dose gap [h]	n	Pred	Obs	GMFE	Pred	Obs	GMFE	Reference
600 mg, iv, 0.5 h	5.0 mg, po, -	0.5	8	3.88	4.83	1.10	9.31	8.88	1.18	Prueksaritanont et al. 2014 [11]
600 mg, iv, 0.5 h	20.0 mg, po, tab	0.5	7	3.30	3.96	1.20	9.07	7.94	1.14	Wu et al. 2017 [21]
600 mg, po, caps	0.05 mg [a], po, sol	0	12/8	3.70	5.88	1.59	8.02	12.41	1.55	Prueksaritanont et al. 2017 [10]
600 mg, po, -	5.0 mg, po, -	0	8	3.66	3.69	1.01	8.71	5.94	1.47	Prueksaritanont et al. 2014 [11]
600 mg, po, tab	10.0 mg [b], po, tab	0	11	3.35	3.52	1.05	9.32	9.96	1.07	Wiebe et al. 2020 [7]
**Overall GMFE**						**1.19 (1.01–1.59)**			**1.28 (1.07–1.55)**	
						**5/5 with GMFE ≤ 2**			**5/5 with GMFE ≤ 2**	

[a] administered as microdose cocktail together with 0.01 mg midazolam, 0.375 mg dabigatran etexilate, 0.01 mg pitavastatin and 0.1 mg atorvastatin,
[b] administered as cocktail together with 0.25 mg digoxin, 1 mg furosemide and 10 mg metformin, **-**: not given, **caps**: capsule, **iv**: intravenous,
n: number of individuals studied, **obs**: observed, **po**: oral, **pred**: predicted, **sol**: solution, **tab**: tablet

5 Gemfibrozil-rosuvastatin drug-drug interaction (DDI)

5.1 DDI modeling

The gemfibrozil-rosuvastatin DDI was modeled using a previously established whole-body parent-metabolite PBPK model of gemfibrozil with its metabolite gemfibrozil 1-O-β-glucuronide [64]. The drug-dependent parameters of this model are reproduced in Table S5.2.1.

The gemfibrozil-rosuvastatin interaction was simulated as competitive inhibition of OATP1B1/1B3, OAT3 and CYP2C9 by gemfibrozil and of OATP1B1/1B3 and OAT3 by gemfibrozil glucuronide. The parameters to model these inhibitions were obtained from literature [65–67], and were included in the gemfibrozil and gemfibrozil 1-O-β-glucuronide drug-dependent parameter Table S5.2.1. The rosuvastatin dose during the gemfibrozil-rosuvastatin DDI was modeled using the split dose approach to describe the still slow absorption of rosuvastatin during this DDI, as indicated by the clinically observed data and the PopPK analysis.

Details on the predicted clinical DDI study are given in Table S5.3.1. Model predictions of rosuvastatin plasma concentration-time profiles before and during gemfibrozil co-administration, compared to observed data, are shown in Figure S5.4.1. Gemfibrozil was administered twice daily for seven days; rosuvastatin was administered as a single dose together with the gemfibrozil morning dose on day 4 (72 h). The strong competitive inhibition of rosuvastatin uptake from the blood into liver and kidney via OATP1B1/1B3 and OAT3 leads to short increases of the simulated rosuvastatin plasma concentrations after every further administration of gemfibrozil (84 h, 96 h, 108 h). The correlation of predicted to observed DDI AUC_{last} ratios and DDI C_{max} ratios is shown in Figure S5.5.1. Table S5.5.1 lists the corresponding predicted and observed DDI AUC_{last} ratios, DDI C_{max} ratios, as well as GMFE values.

5.2 Gemfibrozil and gemfibrozil 1-O-β-glucuronide drug-dependent parameters

The drug-dependent parameters of the gemfibrozil parent-metabolite model are summarized in Table S5.2.1. The associated system-dependent parameters are listed in Table S7.0.1.

Table S5.2.1: Drug-dependent parameters of the gemfibrozil and gemfibrozil 1-O-β-glucuronide PBPK model (adopted from [64])

Parameter	Model	Unit	Source	Literature	Reference	Model	Unit	Source	Literature	Reference	Description
	**Gemfibrozil**					**Gemfibrozil 1-O-β-glucuronide**					
MW	250.33	g/mol	Literature	250.33	[37]	426.46	g/mol	Literature	426.46	[37]	Molecular weight
pKa (acid)	4.70	-	Literature	4.70	[68]	2.68	-	Literature	2.68	[69]	Acid dissociation constant
Solubility (pH)	0.17 (5.9)	g/l	Literature	0.17 (5.9)	[70]	0.79 (7.0)	g/l	Literature	0.79 (7.0)	[37]	Solubility
logP	2.80	-	Literature	2.80, 4.30, 4.77	[69–71]	1.41	-	Optimized	1.22, 2.44	[37]	Lipophilicity
fu	0.65	%	Literature	0.65, 2.1, 3.0	[69, 72, 73]	11.50	%	Literature	11.50, 17.1	[72, 73]	Fraction unbound in plasma
Liver uptake K_m	2.39	µmol/l	Optimized	-	-	-	-	-	-	-	Liver uptake Michaelis-Menten constant
Liver uptake k_{cat}	59.42	1/min	Optimized	-	-	-	-	-	-	-	Liver uptake transport rate constant
OATP1B1 K_m	-	-	-	-	-	0.43	µmol/l	Optimized	-	-	OATP1B1 Michaelis-Menten constant
OATP1B1 k_{cat}	-	-	-	-	-	207.63 [a]	1/min	Optimized	-	-	OATP1B1 transport rate constant
UGT2B7 K_m	2.20	µmol/l	Literature	2.20	[74]	-	-	-	-	-	UGT2B7 Michaelis-Menten constant
UGT2B7 k_{cat}	548.28 [a]	1/min	Optimized	-	-	-	-	-	-	-	UGT2B7 catalytic rate constant
MRP2 K_m	-	-	-	-	-	21.49	µmol/l	Optimized	-	-	MRP2 Michaelis-Menten constant
MRP2 k_{cat}	-	-	-	-	-	125.08 [a]	1/min	Optimized	-	-	MRP2 transport rate constant
GFR fraction	1.00	-	Assumed	-	-	1.00	-	Assumed	-	-	Fraction of filtered drug in the urine
EHC continuous fraction	1.00	-	Assumed	-	-	1.00	-	Assumed	-	-	Fraction of bile continually released
OATP1B1 K_i	15.10	µmol/l	Literature	15.10 [b]	[65]	7.60	µmol/l	Literature	7.60 [b]	[65]	Conc. for half-maximal inhibition
OAT3 K_i	1.47	µmol/l	Literature	1.47 [c]	[66]	9.05	µmol/l	Literature	9.05 [c]	[66]	Conc. for half-maximal inhibition
CYP2C9 K_i	5.80	µmol/l	Literature	5.80 [d]	[67]	-	-	-	-	-	Conc. for half-maximal inhibition
Partition coefficients	Diverse	-	Calculated	Berezh	[75]	Diverse	-	Calculated	PK-Sim	[2]	Cell to plasma partition coefficients
Cellular permeability	7.38E-02	cm/min	Calculated	CdS	[2]	1.22E-04 [e]	cm/min	Calculated	PK-Sim	[76]	Permeability into the cellular space
Intestinal permeability	6.62E-03	cm/min	Optimized	1.62E-04	Calculated	5.98E-07 [e]	cm/min	Calculated	5.98E-07	Calculated	Transcellular intestinal permeability
Tablet Weibull time	24.45	min	Optimized	-	[77–79]	-	-	-	-	-	Dissolution time (50% dissolved)
Tablet Weibull shape	1.56	-	Optimized	-	[77–79]	-	-	-	-	-	Dissolution profile shape

-: not available or process not implemented, [a] compared to [64], reference conc. was changed from assumed to literature value and k_{cat} was converted accordingly, [b] measured with 2-100 µmol/l pravastatin in OATP1B1-expressing oocytes, [c] measured with 0.1 µmol/l pravastatin in human kidney slices, [d] measured with 25-250 µmol/l tolbutamide in pooled human liver microsomes, [e] values were accidentally inverted in [64], **Berezh**: Berezhkovskiy calculation method, **CdS**: Charge-dependent Schmitt calculation method, **conc.**: concentration, **CYP2C9**: cytochrome P450 2C9, **EHC**: enterohepatic circulation, **GFR**: glomerular filtration rate, **MRP2**: multidrug resistance-associated protein 2, **OAT3**: organic anion transporter 3, **OATP1B1**: organic anion transporting polypeptide 1B1, **PK-Sim**: PK-Sim standard calculation method, **UGT2B7**: uridine 5'-diphospho-glucuronosyltransferase 2B7

5.3 Gemfibrozil-rosuvastatin clinical DDI studies

The clinical studies used to evaluate the gemfibrozil-rosuvastatin DDI model performance are summarized in Table S5.3.1.

Table S5.3.1: Gemfibrozil-rosuvastatin DDI study table

**Perpetrator** Gemfibrozil	**Victim** Rosuvastatin	**Dose gap** [**h**]	**n**	**Male** [%]	**Age** [**years**]	**Weight** [**kg**]	**Height** [**cm**]	**BMI** [**kg/m²**]	**Ethnicity**	**Dataset**	**Reference**
600 mg, po, caps, bid	80.0 mg, po, tab	0	20	85	41 ± 6	76 ± 10	172 ± 8	-	American	training	Schneck et al. 2004 [8]

-: not given, **bid**: twice daily, **BMI**: body mass index, **caps**: capsule, **n**: number of individuals studied, **po**: oral, **tab**: tablet, **training**: training dataset (model development and parameter optimization)

5.4 Profiles

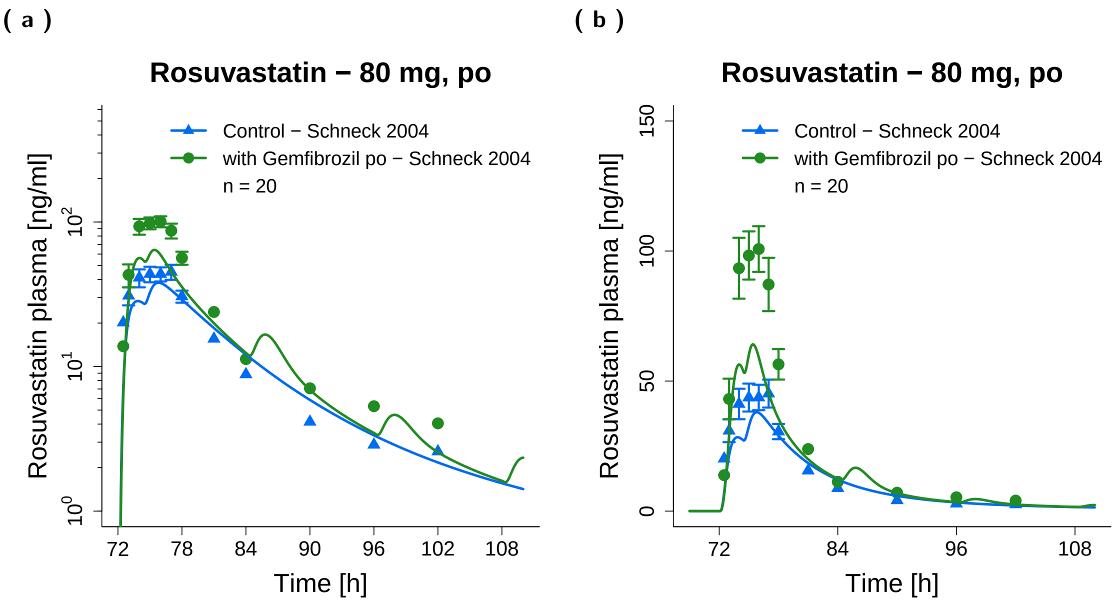

Figure S5.4.1: Rosuvastatin plasma concentration-time profiles (semilogarithmic and linear), before and during gemfibrozil co-administration. Simulations are shown as lines, observed data are shown as dots (training dataset) or triangles (test dataset) ± SD, if available. Details on administration protocols, study population and literature reference are listed in Table S5.3.1

5.5 Model evaluation

5.5.1 DDI AUC$_{last}$ and DDI C$_{max}$ ratio goodness-of-fit plots

(a) DDI AUC$_{last}$ ratios

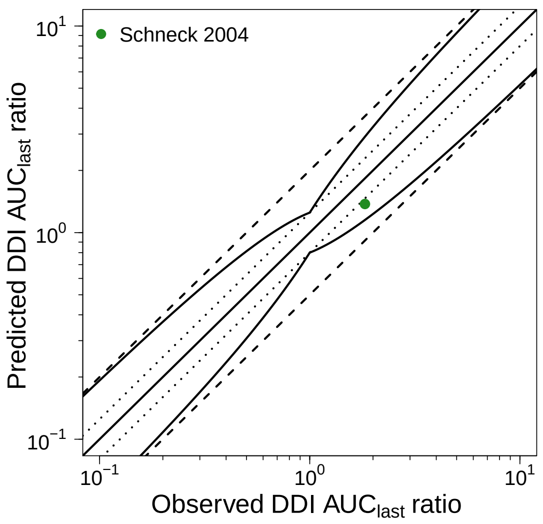

(b) DDI C$_{max}$ ratios

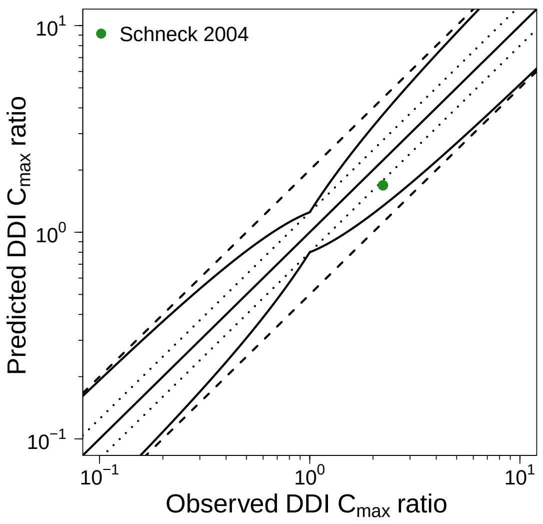

Figure S5.5.1: Predicted versus observed gemfibrozil-rosuvastatin DDI AUC$_{last}$ ratios and DDI C$_{max}$ ratios. The straight solid lines (—–) mark the line of identity. Dotted lines (·····) indicate 1.25-fold, dashed lines (- -) indicate 2-fold deviation. The curved solid lines illustrate the DDI prediction success limits suggested by Guest et al. [63]. Details on administration protocols, study population and literature reference are listed in Table S5.3.1

5.5.2 Geometric mean fold error of predicted DDI AUC_{last} and DDI C_{max} ratios

Table S5.5.1: Predicted and observed gemfibrozil-rosuvastatin DDI AUC_{last} ratios and DDI C_{max} ratios with geometric mean fold errors (GMFEs)

**Perpetrator**	**Victim**			**DDI AUC_{last} ratio**			**DDI C_{max} ratio**			
Gemfibrozil	Rosuvastatin	Dose gap [h]	n	Pred	Obs	GMFE	Pred	Obs	GMFE	Reference
600 mg, po, caps, bid	80.0 mg, po, tab	0	20	1.38	1.83	1.33	1.69	2.23	1.32	Schneck et al. 2004 [8]
**Overall GMFE**						**1.33**<br>**1/1 with GMFE ≤ 2**			**1.32**<br>**1/1 with GMFE ≤ 2**	

bid: twice daily, **caps**: capsule, **n**: number of individuals studied, **obs**: observed, **po**: oral, **pred**: predicted, **tab**: tablet

6 Probenecid-rosuvastatin drug-drug interaction (DDI)

6.1 DDI modeling

The probenecid-rosuvastatin DDI was modeled using a previously established whole-body PBPK model of probenecid [80]. The drug-dependent parameters of this model are reproduced in Table S6.2.1.

The probenecid-rosuvastatin interaction was simulated as competitive inhibition of OATP1B1/1B3 and OAT3 by probenecid. The parameters to model these inhibitions were obtained from literature [81, 82], and were included in the probenecid drug-dependent parameter Table S6.2.1. To account for the impact of probenecid on the absorption of rosuvastatin, the rosuvastatin dose during the probenecid-rosuvastatin DDI was modeled as a single dose without a lag time, as indicated by the PopPK analysis.

Details on the predicted clinical DDI study are given in Table S6.3.1. Model predictions of rosuvastatin plasma concentration-time profiles before and during probenecid co-administration, compared to observed data, are shown in Figure S6.4.1. Predicted compared to observed rosuvastatin fraction excreted in urine before and during probenecid co-administration are shown in Figure S6.4.2. The correlation of predicted to observed DDI AUC_{last} ratios and DDI C_{max} ratios is shown in Figure S6.5.1. Table S6.5.1 lists the corresponding predicted and observed DDI AUC_{last} ratios, DDI C_{max} ratios, as well as GMFE values.

6.2 Probenecid drug-dependent parameters

The drug-dependent parameters of the probenecid model are summarized in Table S6.2.1. The associated system-dependent parameters are listed in Table S7.0.1.

Table S6.2.1: Drug-dependent parameters of the probenecid PBPK model (adopted from [80])

Parameter	Model	Unit	Source	Literature	Reference	Description
MW	285.36	g/mol	Literature	285.36	[37]	Molecular weight
pKa (acid)	3.70	-	Literature	3.01, 3.70	[83, 84]	Acid dissociation constant
Solubility	0.74 (HIF)	g/l	Literature	0.74 (HIF), 1.29 (pH 6.50), 1.63 (FaSSIF)	[85]	Solubility
logP	1.34	-	Optimized	-0.52, -0.23, 0.13, 3.21, 3.70	[83, 84, 86, 87]	Lipophilicity
fu	11.70	%	Literature	6.20, 7.56, 11.70	[88–90]	Fraction unbound in plasma
OAT3 K_m	12.18	µmol/l	Optimized	-	-	OAT3 Michaelis-Menten constant
OAT3 k_{cat}	1966.57	1/min	Optimized	-	-	OAT3 transport rate constant
UGT1A9 K_m	198.30	µmol/l	Literature	198.30	[91]	UGT1A9 Michaelis-Menten constant
UGT1A9 k_{cat}	74.92	1/min	Optimized	-	-	UGT1A9 catalytic rate constant
GFR fraction	0.03	-	Optimized	-	[89, 90]	Fraction of filtered drug in the urine
EHC continuous fraction	1.00	-	Assumed	-	-	Fraction of bile continually released
OATP1B1 K_i	39.80	µmol/l	Literature	39.80 [a]	[81]	Conc. for half-maximal inhibition
OAT3 K_i	7.25	µmol/l	Literature	7.25	[82]	Conc. for half-maximal inhibition
Partition coefficients	Diverse	-	Calculated	PK-Sim	[2]	Cell to plasma partition coefficients
Cellular permeability	1.17E-3	cm/min	Calculated	CdS norm	[2]	Permeability into the cellular space
Intestinal permeability	3.97E-4	cm/min	Optimized	3.12E-6	Calculated	Transcellular intestinal permeability
Tablet fasted Weibull time	44.25	min	Optimized	-	[7, 92–94]	Dissolution time (50% dissolved)
Tablet fasted Weibull shape	0.58	-	Optimized	-	[7, 92–94]	Dissolution profile shape

[a] measured with 3.0 µmol/l of 2',7'-dichlorofluorescein in OATP1B1-expressing human embryonic kidney 293 cells, **CdS norm**: charge-dependent Schmitt normalized to PK-Sim calculation method, **conc.**: concentration, **EHC**: enterohepatic circulation, **FaSSIF**: fasted state simulated intestinal fluid, **GFR**: glomerular fitration rate, **HIF**: human intestinal fluid, **OAT3**: organic anion transporter 3, **OATP1B1**: organic anion transporting polypeptide 1B1, **PK-Sim**: PK-Sim standard calculation method, **UGT1A9**: uridine 5'-diphospho-glucuronosyltransferase 1A9

6.3 Probenecid-rosuvastatin clinical DDI studies

The clinical studies used to evaluate the probenecid-rosuvastatin DDI model performance are summarized in Table S6.3.1.

Table S6.3.1: Probenecid-rosuvastatin DDI study table

**Perpetrator** Probenecid	**Victim** Rosuvastatin	**Dose gap [h]**	**n**	**Male** [%]	**Age** [years]	**Weight** [kg]	**Height** [cm]	**BMI** [kg/m²]	**Ethnicity**	**Dataset**	**Reference**
1000 mg, po, tab, bid	10.0 mg [a], po, tab	1	13	100	34 ± 10 (21-51)	78 ± 10 (62-95)	179 ± 5 (169-186)	24 ± 3 (19-29)	European	training	Wiebe et al. 2020 [7]

[a] administered as cocktail together with 0.25 mg digoxin, 1 mg furosemide and 10 mg metformin, **bid**: twice daily, **BMI**: body mass index, **n**: number of individuals studied, **po**: oral, **tab**: tablet, **training**: training dataset (model development and parameter optimization)

6.4 Profiles

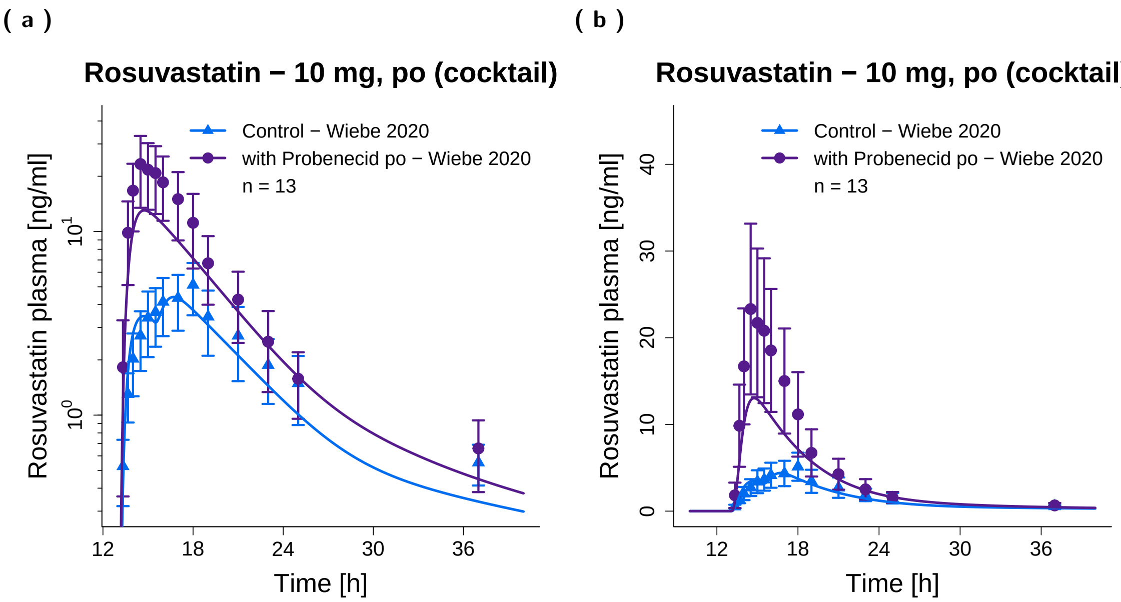

Figure S6.4.1: Rosuvastatin plasma concentration-time profiles (semilogarithmic and linear), before and during probenecid co-administration. Simulations are shown as lines, observed data are shown as dots (training dataset) or triangles (test dataset) ± SD. Details on administration protocols, study population and literature reference are listed in Table S6.3.1

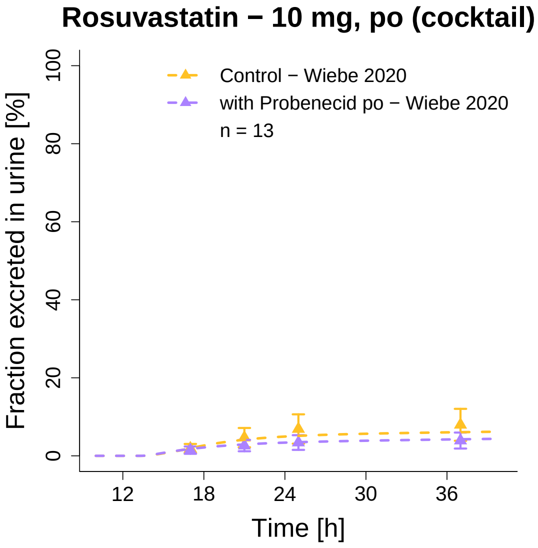

Figure S6.4.2: Rosuvastatin fraction excreted in urine profiles, before and during probenecid co-administration. Simulations are shown as lines, observed data are shown as triangles (test dataset) ± SD. Details on administration protocols, study population and literature reference are listed in Table S6.3.1

6.5 Model evaluation

6.5.1 DDI AUC_{last} and DDI C_{max} ratio goodness-of-fit plots

(a) DDI AUC_{last} ratios

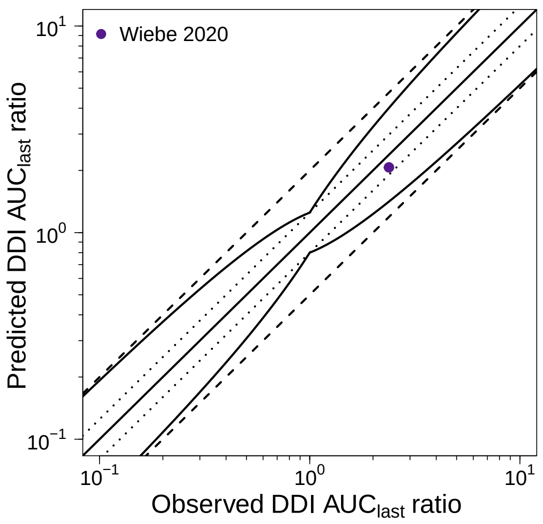

(b) DDI C_{max} ratios

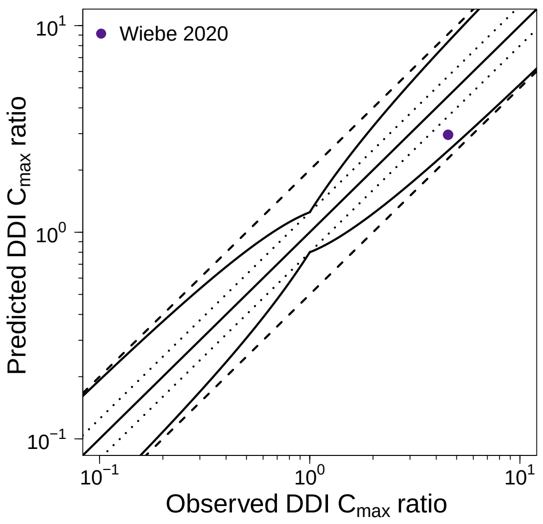

Figure S6.5.1: Predicted versus observed probenecid-rosuvastatin DDI AUC_{last} ratios and DDI C_{max} ratios. The straight solid lines (—) mark the line of identity. Dotted lines (·····) indicate 1.25-fold, dashed lines (- -) indicate 2-fold deviation. The curved solid lines illustrate the DDI prediction success limits suggested by Guest et al. [63]. Details on administration protocols, study population and literature reference are listed in Table S6.3.1

6.5.2 Geometric mean fold error of predicted DDI AUC_{last} and DDI C_{max} ratios

Table S6.5.1: Predicted and observed probenecid-rosuvastatin DDI AUC_{last} ratios and DDI C_{max} ratios with geometric mean fold errors (GMFEs)

**Perpetrator**	**Victim**			**DDI AUC_{last} ratio**			**DDI C_{max} ratio**			
Probenecid	Rosuvastatin	Dose gap [h]	n	Pred	Obs	GMFE	Pred	Obs	GMFE	Reference
1000 mg, po, tab, bid	10 mg [a], po, tab	1	13	2.07	2.38	1.15	2.96	4.55	1.54	Wiebe et al. 2020 [7]
**Overall GMFE**						**1.15**<br>**1/1 with GMFE ≤ 2**			**1.54**<br>**1/1 with GMFE ≤ 2**	

[a] administered as cocktail together with 0.25 mg digoxin, 1 mg furosemide and 10 mg metformin, **bid**: twice daily, **n**: number of individuals studied, **obs**: observed, **po**: oral, **pred**: predicted, **tab**: tablet

7 System-dependent parameters

Details on the expression of metabolizing enzymes and drug transporters implemented to model the pharmacokinetics of rosuvastatin, rifampicin, gemfibrozil with gemfibrozil 1-O-β-glucuronide, and probenecid are summarized in Table S7.0.1.

Table S7.0.1: System-dependent parameters

	Reference concentration					Half-life [h]	
**Enzyme/Transporter**	**Mean** [a]	**GeoSD** [b]	**Expression profile** [c]	**Localization**	**Direction**	**Liver**	**Intestine**
AADAC	1.00 [d] [95]	1.40 [e]	RT-PCR [98]	Intracellular	-	36	23
CYP2C9	3.84 [99]	2.01 [1]	RT-PCR [100]	Intracellular	-	104	23
UGT1A9	0.197 [f] [96, 101]	1.12 [1]	RT-PCR [98]	Intracellular	-	36	23
UGT2B7	0.095 [f] [96, 101]	1.60 [1]	EST [102]	Intracellular	-	36	23
BCRP	0.025 [g] [97, 103]	1.35 [103]	Array [104]	Apical	Efflux	36	23
MRP2	0.057 [g] [97, 105]	1.49 [105]	Array [104]	Apical	Efflux	36	23
OAT3	0.092 [f] [96, 106]	1.53 [106]	RT-PCR [107]	Basolateral	Influx	36	-
OATP1B1	0.074 [g] [97]	1.54 [97]	RT-PCR [107]	Basolateral	Influx	36	-
OATP2B1	0.110 [g] [97]	1.41 [97]	Array [104]	Apical/Basolateral	Influx	36	23
Pgp	0.077 [f] [96, 106]	1.46 [106]	RT-PCR [107], intestinal mucosa → factor 3.57 [47]	Apical	Efflux	36	23

AADAC: arylacetamide deacetylase, **BCRP**: breast cancer resistance protein, **CYP2C9**: cytochrome P450 2C9, **MRP2**: multidrug resistance-associated protein 2, **OAT3**: organic anion transporter 3, **OATP1B1**: organic anion transporting polypeptide 1B1, **OATP2B1**: organic anion transporting polypeptide 2B1, **Pgp**: P-glycoprotein, **UGT1A9**: uridine 5'-diphospho-glucuronosyltransferase 1A9, **UGT2B7**: uridine 5'-diphospho-glucuronosyltransferase 2B7, [a] µmol protein/l in the tissue of highest expression, [b] geometric standard deviation of the reference concentration, [c] in the different organs (PK-Sim® expression database profile), [d] if no information was available, the mean reference concentration was set to 1.0 µmol/l and the catalytic rate constant (k_{cat}) was optimized [95], [e] if no information was available, a moderate variability of 35 % CV was assumed (= 1.40 GeoSD), [f] calculated from enzyme or transporter amount per mg membrane protein × 26.2 mg human kidney microsomal protein per g kidney [96], [g] calculated from transporter amount per mg membrane protein × 37.0 mg human membrane protein per g liver [97]

References

[1] Open Systems Pharmacology Suite Community (2018) PK-Sim® Ontogeny Database Documentation, Version 7.3. https://github.com/Open-Systems-Pharmacology/OSPSuite.Documentation/blob/master/PK-SimOntogenyDatabaseVersion7.3.pdf, accessed: 2020-02-25

[2] Open Systems Pharmacology Suite Community (2018) Open Systems Pharmacology Suite Manual, Version 7.4. https://github.com/Open-Systems-Pharmacology/OSPSuite.Documentation/blob/master/OpenSystemsPharmacologySuite.pdf, accessed: 2020-02-25

[3] Wang Q, Zheng M, Leil T (2017) Investigating Transporter-Mediated Drug-Drug Interactions Using a Physiologically Based Pharmacokinetic Model of Rosuvastatin. CPT: pharmacometrics & systems pharmacology 6(4):228–238

[4] Martin PD, Warwick MJ, Dane AL, Brindley C, Short T (2003) Absolute oral bioavailability of rosuvastatin in healthy white adult male volunteers. Clinical therapeutics 25(10):2553–63

[5] Stopfer P, Giessmann T, Hohl K, Sharma A, Ishiguro N, Taub ME, Zimdahl-Gelling H, Gansser D, Wein M, Ebner T, Müller F (2016) Pharmacokinetic Evaluation of a Drug Transporter Cocktail Consisting of Digoxin, Furosemide, Metformin, and Rosuvastatin. Clinical pharmacology and therapeutics 100(3):259–67

[6] Stopfer P, Giessmann T, Hohl K, Hutzel S, Schmidt S, Gansser D, Ishiguro N, Taub ME, Sharma A, Ebner T, Müller F (2018) Optimization of a drug transporter probe cocktail: potential screening tool for transporter-mediated drug-drug interactions. British journal of clinical pharmacology 84(9):1941–1949

[7] Wiebe ST, Giessmann T, Hohl K, Schmidt-Gerets S, Hauel E, Jambrecina A, Bader K, Ishiguro N, Taub ME, Sharma A, Ebner T, Mikus G, Fromm MF, Müller F, Stopfer P (2020) Validation of a Drug Transporter Probe Cocktail Using the Prototypical Inhibitors Rifampin, Probenecid, Verapamil, and Cimetidine. Clinical pharmacokinetics

[8] Schneck DW, Birmingham BK, Zalikowski JA, Mitchell PD, Wang Y, Martin PD, Lasseter KC, Brown CDA, Windass AS, Raza A (2004) The effect of gemfibrozil on the pharmacokinetics of rosuvastatin. Clinical pharmacology and therapeutics 75(5):455–63

[9] Billington S, Shoner S, Lee S, Clark-Snustad K, Pennington M, Lewis D, Muzi M, Rene S, Lee J, Nguyen TB, Kumar V, Ishida K, Chen L, Chu X, Lai Y, Salphati L, Hop CECA, Xiao G, Liao M, Unadkat JD (2019) Positron Emission Tomography Imaging of [11 C]Rosuvastatin Hepatic Concentrations and Hepatobiliary Transport in Humans in the Absence and Presence of Cyclosporin A. Clinical pharmacology and therapeutics 106(5):1056–1066

[10] Prueksaritanont T, Tatosian DA, Chu X, Railkar R, Evers R, Chavez-Eng C, Lutz R, Zeng W, Yabut J, Chan GH, Cai X, Latham AH, Hehman J, Stypinski D, Brejda J, Zhou C, Thornton B, Bateman KP, Fraser I, Stoch SA (2017) Validation of a microdose probe drug cocktail for clinical drug interaction assessments for drug transporters and CYP3A. Clinical pharmacology and therapeutics 101(4):519–530

[11] Prueksaritanont T, Chu X, Evers R, Klopfer SO, Caro L, Kothare PA, Dempsey C, Rasmussen S, Houle R, Chan G, Cai X, Valesky R, Fraser IP, Stoch SA (2014) Pitavastatin is a more sensitive and selective organic anion-transporting polypeptide 1B clinical probe than rosuvastatin. British journal of clinical pharmacology 78(3):587–98

[12] Cooper KJ, Martin PD, Dane AL, Warwick MJ, Schneck DW, Cantarini MV (2003) Effect of itraconazole on the pharmacokinetics of rosuvastatin. Clinical pharmacology and therapeutics 73(4):322–9

[13] Csonka D, Bruderer S, Schultz A, Soergel M, Stepanova R, Sabattini G, Perez-Ruixo JJ (2019) Effect of Macitentan on the Pharmacokinetics of the Breast Cancer Resistance Protein Substrates, Rosuvastatin and Riociguat, in Healthy Male Subjects. Clinical drug investigation 39(12):1223–1232

[14] Huguet J, Lu J, Gaudette F, Chiasson JL, Hamet P, Michaud V, Turgeon J (2016) No effects of pantoprazole on the pharmacokinetics of rosuvastatin in healthy subjects. European journal of clinical pharmacology 72(8):925–31

[15] Martin PD, Warwick MJ, Dane AL, Cantarini MV (2003) A double-blind, randomized, incomplete crossover trial to assess the dose proportionality of rosuvastatin in healthy volunteers. Clinical therapeutics 25(8):2215–24

[16] Stopfer P, Giessmann T, Hohl K, Sharma A, Ishiguro N, Taub ME, Jungnik A, Gansser D, Ebner T, Müller F (2018) Effects of Metformin and Furosemide on Rosuvastatin Pharmacokinetics in Healthy Volunteers: Implications for Their Use as Probe Drugs in a Transporter Cocktail. European journal of drug metabolism and pharmacokinetics 43(1):69–80

[17] Coss CC, Jones A, Dalton JT (2016) Pharmacokinetic drug interactions of the selective androgen receptor modulator GTx-024(Enobosarm) with itraconazole, rifampin, probenecid, celecoxib and rosuvastatin. Investigational new drugs 34(4):458–67

[18] Martin PD, Warwick MJ, Dane AL, Hill SJ, Giles PB, Phillips PJ, Lenz E (2003) Metabolism, excretion, and pharmacokinetics of rosuvastatin in healthy adult male volunteers. Clinical therapeutics 25(11):2822–35

[19] US Food and Drug Administration (FDA) (2003) Approval Package for Application Number 21-366 (Crestor), Clinical Pharmacology and Biopharmaceutics Review

[20] Edwards JE, Eliot L, Parkinson A, Karan S, MacConell L (2017) Assessment of Pharmacokinetic Interactions Between Obeticholic Acid and Caffeine, Midazolam, Warfarin, Dextromethorphan, Omeprazole, Rosuvastatin, and Digoxin in Phase 1 Studies in Healthy Subjects. Advances in therapy 34(9):2120–2138

[21] Wu HF, Hristeva N, Chang J, Liang X, Li R, Frassetto L, Benet LZ (2017) Rosuvastatin Pharmacokinetics in Asian and White Subjects Wild Type for Both OATP1B1 and BCRP Under Control and Inhibited Conditions. Journal of pharmaceutical sciences 106(9):2751–2757

[22] Birmingham BK, Bujac SR, Elsby R, Azumaya CT, Wei C, Chen Y, Mosqueda-Garcia R, Ambrose HJ (2015) Impact of ABCG2 and SLCO1B1 polymorphisms on pharmacokinetics of rosuvastatin, atorvastatin and simvastatin acid in Caucasian and Asian subjects: a class effect? European journal of clinical pharmacology 71(3):341–55

[23] Jones NS, Yoshida K, Salphati L, Kenny JR, Durk MR, Chinn LW (2020) Complex DDI by Fenebrutinib and the Use of Transporter Endogenous Biomarkers to Elucidate the Mechanism of DDI. Clinical pharmacology and therapeutics 107(1):269–277

[24] Lee J, Rhee SJ, Lee S, Yu KS (2018) Evaluation of drug interactions between fimasartan and rosuvastatin after single and multiple doses in healthy Caucasians. Drug design, development and therapy 12:787–794

[25] Martin P, Gillen M, Ritter J, Mathews D, Brealey C, Surry D, Oliver S, Holmes V, Severin P, Elsby R (2016) Effects of Fostamatinib on the Pharmacokinetics of Oral Contraceptive, Warfarin, and the Statins Rosuvastatin and Simvastatin: Results From Phase I Clinical Studies. Drugs in R&D 16(1):93–107

[26] Willis BA, Andersen SW, Ayan-Oshodi M, James DE, Liffick E, Hillgren K, Guo Y, Monk SA (2020) Assessment of Transporter Polymorphisms as a Factor in a BCRP Drug Interaction Study With Lanabecestat. Journal of clinical pharmacology 60(1):107–116

[27] Martin PD, Dane AL, Nwose OM, Schneck DW, Warwick MJ (2002) No effect of age or gender on the pharmacokinetics of rosuvastatin: a new HMG-CoA reductase inhibitor. Journal of clinical pharmacology 42(10):1116–21

[28] Gidal BE, Mintzer S, Schwab M, Schutz R, Kharidia J, Blum D, Grinnell T, Sunkaraneni S (2017) Evidence for a pharmacokinetic interaction between eslicarbazepine and rosuvastatin: Potential effects on xenobiotic transporters. Epilepsy research 135:64–70

[29] Lee E, Ryan S, Birmingham B, Zalikowski J, March R, Ambrose H, Moore R, Lee C, Chen Y, Schneck D (2005) Rosuvastatin pharmacokinetics and pharmacogenetics in white and Asian subjects residing in the same environment. Clinical pharmacology and therapeutics 78(4):330–41

[30] Cooper KJ, Martin PD, Dane AL, Warwick MJ, Schneck DW, Cantarini MV (2002) The effect of fluconazole on the pharmacokinetics of rosuvastatin. European journal of clinical pharmacology 58(8):527–31

[31] Cooper KJ, Martin PD, Dane AL, Warwick MJ, Raza A, Schneck DW (2003) Lack of effect of ketoconazole on the pharmacokinetics of rosuvastatin in healthy subjects. British journal of clinical pharmacology 55(1):94–9

[32] Cooper KJ, Martin PD, Dane AL, Warwick MJ, Raza A, Schneck DW (2003) The effect of erythromycin on the pharmacokinetics of rosuvastatin. European journal of clinical pharmacology 59(1):51–6

[33] Kosoglou T, Statkevich P, Yang B, Suresh R, Zhu Y, Boutros T, Maxwell SE, Tiessen R, Cutler DL (2004) Pharmacodynamic interaction between ezetimibe and rosuvastatin. Current medical research and opinion 20(8):1185–95

[34] Martin PD, Mitchell PD, Schneck DW (2002) Pharmacodynamic effects and pharmacokinetics of a new HMG-CoA reductase inhibitor, rosuvastatin, after morning or evening administration in healthy volunteers. British journal of clinical pharmacology 54(5):472–7

[35] US Food and Drug Administration (FDA) (2018) Approval Package for NDA 210450 (Orilissa), Multi-Disciplinary Review and Evaluation

[36] Gosai P, Liu J, Doyle RT, Johnson J, Carter R, Sica D, McKenney JM (2008) Effect of omega-3-acid ethyl esters on the steady-state plasma pharmacokinetics of rosuvastatin in healthy adults. Expert opinion on pharmacotherapy 9(17):2947–53

[37] Wishart DS, Knox C, Guo AC, Shrivastava S, Hassanali M, Stothard P, Chang Z, Woolsey J (2006) DrugBank: a comprehensive resource for in silico drug discovery and exploration. Nucleic Acids Research 34(Supplement 1):D668–D672

[38] Riccardi KA, Tess DA, Lin J, Patel R, Ryu S, Atkinson K, Di L, Li R (2019) A Novel Unified Approach to Predict Human Hepatic Clearance for Both Enzyme- and Transporter-Mediated Mechanisms Using Suspended Human Hepatocytes. Drug metabolism and disposition: the biological fate of chemicals 47(5):484–492

[39] Australian Therapeutic Goods Administration (TGA) (2011) Crestor - Product Information

[40] McTaggart F, Buckett L, Davidson R, Holdgate G, McCormick A, Schneck D, Smith G, Warwick M (2001) Preclinical and clinical pharmacology of Rosuvastatin, a new 3-hydroxy-3-methylglutaryl coenzyme A reductase inhibitor. The American journal of cardiology 87(5A):28B–32B

[41] Jones HM, Barton HA, Lai Y, Bi YA, Kimoto E, Kempshall S, Tate SC, El-Kattan A, Houston JB, Galetin A, Fenner KS (2012) Mechanistic pharmacokinetic modeling for the prediction of transporter-mediated disposition in humans from sandwich culture human hepatocyte data. Drug metabolism and disposition: the biological fate of chemicals 40(5):1007–17

[42] US Food and Drug Administration (FDA) (2003) Approval Package for Application Number 21-366 (Crestor), Pharmacology Review Part 2

[43] Kitamura S, Maeda K, Wang Y, Sugiyama Y (2008) Involvement of multiple transporters in the hepatobiliary transport of rosuvastatin. Drug metabolism and disposition: the biological fate of chemicals 36(10):2014–23

[44] Windass AS, Lowes S, Wang Y, Brown CDA (2007) The contribution of organic anion transporters OAT1 and OAT3 to the renal uptake of rosuvastatin. The Journal of pharmacology and experimental therapeutics 322(3):1221–7

[45] Goard CA, Mather RG, Vinepal B, Clendening JW, Martirosyan A, Boutros PC, Sharom FJ, Penn LZ (2010) Differential interactions between statins and P-glycoprotein: implications for exploiting statins as anticancer agents. International journal of cancer 127(12):2936–48

[46] Seo KS, Han HK (2019) Multilayer-Coated Tablet of Clopidogrel and Rosuvastatin: Preparation and In Vitro/In vivo Characterization. Pharmaceutics 11(7)

[47] Hanke N, Frechen S, Moj D, Britz H, Eissing T, Wendl T, Lehr T (2018) PBPK models for CYP3A4 and P-gp DDI prediction: A modeling network of rifampicin, itraconazole, clarithromycin, midazolam, alfentanil, and digoxin. CPT: Pharmacometrics & Systems Pharmacology 7(10):647–659

[48] Zhang Y, Panfen E, Fancher M, Sinz M, Marathe P, Shen H (2019) Dissecting the Contribution of OATP1B1 to Hepatic Uptake of Statins Using the OATP1B1 Selective Inhibitor Estropipate. Molecular pharmaceutics 16(6):2342–2353

[49] Bi YA, Costales C, Mathialagan S, West M, Eatemadpour S, Lazzaro S, Tylaska L, Scialis R, Zhang H, Umland J, Kimoto E, Tess DA, Feng B, Tremaine LM, Varma MVS, Rodrigues AD (2019) Quantitative Contribution of Six Major Transporters to the Hepatic Uptake of Drugs: "SLC-Phenotyping" Using Primary Human Hepatocytes. The Journal of pharmacology and experimental therapeutics 370(1):72–83

[50] Yoshida K, Maeda K, Sugiyama Y (2012) Transporter-mediated drug–drug interactions involving OATP substrates: predictions based on in vitro inhibition studies. Clinical pharmacology and therapeutics 91(6):1053–64

[51] Merck Research Laboratories (2006) The Merck Index 14th edition: Rifampin. Merck & Co., Inc., Whitehouse Station, NJ, USA

[52] Boman G, Ringberger VA (1974) Binding of rifampicin by human plasma proteins. European journal of clinical pharmacology 7(5):369–73

[53] Baneyx G, Parrott N, Meille C, Iliadis A, Lavé T (2014) Physiologically based pharmacokinetic modeling of CYP3A4 induction by rifampicin in human: influence of time between substrate and inducer administration. European journal of pharmaceutical sciences : official journal of the European Federation for Pharmaceutical Sciences 56:1–15

[54] Templeton IE, Houston JB, Galetin A (2011) Predictive utility of in vitro rifampin induction data generated in fresh and cryopreserved human hepatocytes, Fa2N-4, and HepaRG cells. Drug metabolism and disposition: the biological fate of chemicals 39(10):1921–9

[55] Loos U, Musch E, Jensen JC, Mikus G, Schwabe HK, Eichelbaum M (1985) Pharmacokinetics of oral and intravenous rifampicin during chronic administration. Klinische Wochenschrift 63(23):1205–11

[56] Tirona RG, Leake BF, Wolkoff AW, Kim RB (2003) Human organic anion transporting polypeptide-C (SLC21A6) is a major determinant of rifampin-mediated pregnane X receptor activation. The Journal of pharmacology and experimental therapeutics 304(1):223–8

[57] Nakajima A, Fukami T, Kobayashi Y, Watanabe A, Nakajima M, Yokoi T (2011) Human arylacetamide deacetylase is responsible for deacetylation of rifamycins: rifampicin, rifabutin, and rifapentine. Biochemical pharmacology 82(11):1747–56

[58] Collett A, Tanianis-Hughes J, Hallifax D, Warhurst G (2004) Predicting P-glycoprotein effects on oral absorption: correlation of transport in Caco-2 with drug pharmacokinetics in wild-type and mdr1a(-/-) mice in vivo. Pharmaceutical research 21(5):819–26

[59] Shou M, Hayashi M, Pan Y, Xu Y, Morrissey K, Xu L, Skiles GL (2008) Modeling, prediction, and in vitro in vivo correlation of CYP3A4 induction. Drug metabolism and disposition: the biological fate of chemicals 36(11):2355–70

[60] Greiner B, Eichelbaum M, Fritz P, Kreichgauer HP, von Richter O, Zundler J, Kroemer HK (1999) The role of intestinal P-glycoprotein in the interaction of digoxin and rifampin. The Journal of clinical investigation 104(2):147–53

[61] Rodgers T, Leahy D, Rowland M (2005) Physiologically based pharmacokinetic modeling 1: Predicting the tissue distribution of moderate-to-strong bases. Journal of Pharmaceutical Sciences 94(6):1259–1276

[62] Rodgers T, Rowland M (2006) Physiologically based pharmacokinetic modelling 2: Predicting the tissue distribution of acids, very weak bases, neutrals and zwitterions. Journal of Pharmaceutical Sciences 95(6):1238–1257

[63] Guest EJ, Aarons L, Houston JB, Rostami-Hodjegan A, Galetin A (2011) Critique of the two-fold measure of prediction success for ratios: Application for the assessment of drug-drug interactions. Drug Metabolism and Disposition 39(2):170–173

[64] Türk D, Hanke N, Wolf S, Frechen S, Eissing T, Wendl T, Schwab M, Lehr T (2019) Physiologically Based Pharmacokinetic Models for Prediction of Complex CYP2C8 and OATP1B1

(SLCO1B1) Drug-Drug-Gene Interactions: A Modeling Network of Gemfibrozil, Repaglinide, Pioglitazone, Rifampicin, Clarithromycin and Itraconazole. Clinical pharmacokinetics 58(12):1595–1607

[65] Nakagomi-Hagihara R, Nakai D, Tokui T, Abe T, Ikeda T (2007) Gemfibrozil and its glucuronide inhibit the hepatic uptake of pravastatin mediated by OATP1B1. Xenobiotica; the fate of foreign compounds in biological systems 37(5):474–86

[66] Watanabe T, Kusuhara H, Watanabe T, Debori Y, Maeda K, Kondo T, Nakayama H, Horita S, Ogilvie BW, Parkinson A, Hu Z, Sugiyama Y (2011) Prediction of the overall renal tubular secretion and hepatic clearance of anionic drugs and a renal drug-drug interaction involving organic anion transporter 3 in humans by in vitro uptake experiments. Drug metabolism and disposition: the biological fate of chemicals 39(6):1031–8

[67] Wen X, Wang JS, Backman JT, Kivistö KT, Neuvonen PJ (2001) Gemfibrozil is a potent inhibitor of human cytochrome P450 2C9. Drug metabolism and disposition: the biological fate of chemicals 29(11):1359–61

[68] Fernández L, Machín R, Zornoza A, Vélaz I, Martín C, Martínez-Ohárriz MC (2011) Mechanism of sorption and release of a weak acid from β-cyclodextrin polymers. Journal of Inclusion Phenomena and Macrocyclic Chemistry 69(3-4):411–5

[69] Varma MVS, Lai Y, Kimoto E, Goosen TC, El-Kattan AF, Kumar V (2013) Mechanistic modeling to predict the transporter- and enzyme-mediated drug-drug interactions of repaglinide. Pharmaceutical research 30(4):1188–99

[70] Luner PE, Babu SR, Radebaugh GW (1994) The effects of bile salts and lipids on the physicochemical behavior of gemfibrozil. Pharmaceutical research 11(12):1755–60

[71] Westerhoff P, Yoon Y, Snyder S, Wert E (2005) Fate of endocrine-disruptor, pharmaceutical, and personal care product chemicals during simulated drinking water treatment processes. Environmental science & technology 39(17):6649–63

[72] Shitara Y, Hirano M, Sato H, Sugiyama Y (2004) Gemfibrozil and its glucuronide inhibit the organic anion transporting polypeptide 2 (OATP2/OATP1B1:SLC21A6)-mediated hepatic uptake and CYP2C8-mediated metabolism of cerivastatin: analysis of the mechanism of the clinically relevant drug-drug interaction. The Journal of pharmacology and experimental therapeutics 311(1):228–36

[73] Sabordo L, Sallustio BC, Evans AM, Nation RL (2000) Hepatic disposition of the acyl glucuronide 1-O-gemfibrozil-beta-D-glucuronide: effects of clofibric acid, acetaminophen, and acetaminophen glucuronide. The Journal of pharmacology and experimental therapeutics 295(1):44–50

[74] Mano Y, Usui T, Kamimura H (2007) The UDP-glucuronosyltransferase 2B7 isozyme is responsible for gemfibrozil glucuronidation in the human liver. Drug metabolism and disposition: the biological fate of chemicals 35(11):2040–4

[75] Berezhkovskiy LM (2004) Volume of distribution at steady state for a linear pharmacokinetic system with peripheral elimination. Journal of pharmaceutical sciences 93(6):1628–40

[76] Kawai R, Lemaire M, Steimer JL, Bruelisauer A, Niederberger W, Rowland M (1994) Physiologically based pharmacokinetic study on a cyclosporin derivative, SDZ IMM 125. Journal of pharmacokinetics and biopharmaceutics 22(5):327–65

[77] Honkalammi J, Niemi M, Neuvonen PJ, Backman JT (2011) Dose-dependent interaction between gemfibrozil and repaglinide in humans: strong inhibition of CYP2C8 with subtherapeutic gemfibrozil doses. Drug metabolism and disposition: the biological fate of chemicals 39(10):1977–86

[78] Honkalammi J, Niemi M, Neuvonen PJ, Backman JT (2012) Gemfibrozil is a strong inactivator of CYP2C8 in very small multiple doses. Clinical pharmacology and therapeutics 91(5):846–55

[79] Backman JT, Honkalammi J, Neuvonen M, Kurkinen KJ, Tornio A, Niemi M, Neuvonen PJ (2009) CYP2C8 activity recovers within 96 hours after gemfibrozil dosing: estimation of CYP2C8 half-life using repaglinide as an in vivo probe. Drug metabolism and disposition: the biological fate of chemicals 37(12):2359–66

[80] Britz H, Hanke N, Taub ME, Wang T, Prasad B, Fernandez É, Stopfer P, Nock V, Lehr T (2020) Physiologically Based Pharmacokinetic Models of Probenecid and Furosemide to Predict Transporter Mediated Drug-Drug Interactions. Pharmaceutical research 37(12):250

[81] Izumi S, Nozaki Y, Komori T, Takenaka O, Maeda K, Kusuhara H, Sugiyama Y (2016) Investigation of Fluorescein Derivatives as Substrates of Organic Anion Transporting Polypeptide (OATP) 1B1 To Develop Sensitive Fluorescence-Based OATP1B1 Inhibition Assays. Molecular pharmaceutics 13(2):438–48

[82] Tsuruya Y, Kato K, Sano Y, Imamura Y, Maeda K, Kumagai Y, Sugiyama Y, Kusuhara H (2016) Investigation of Endogenous Compounds Applicable to Drug-Drug Interaction Studies Involving the Renal Organic Anion Transporters, OAT1 and OAT3, in Humans. Drug metabolism and disposition: the biological fate of chemicals 44(12):1925–1933

[83] Avdeef A (2001) Physicochemical profiling (solubility, permeability and charge state). Current topics in medicinal chemistry 1(4):277–351

[84] Avdeef A (2003) Absorption and Drug Development: Solubility, Permeability, and Charge State. John Wiley & Sons, Inc., Hoboken, NJ

[85] Söderlind E, Karlsson E, Carlsson A, Kong R, Lenz A, Lindborg S, Sheng JJ (2010) Simulating fasted human intestinal fluids: understanding the roles of lecithin and bile acids. Molecular pharmaceutics 7(5):1498–507

[86] DAYTON PG, YU TF, CHEN W, BERGER L, WEST LA, GUTMAN AB (1963) The physiological disposition of probenecid, including renal clearance, in man, studied by an improved method for its estimation in biological material. The Journal of pharmacology and experimental therapeutics 140(3):278–86

[87] Hansch C, Leo A, Hoekman D (1995) Exploring QSAR: Hydrophobic, electronic, and steric constants. American Chemical Society, Washington, DC

[88] Shen H, Holenarsipur VK, Mariappan TT, Drexler DM, Cantone JL, Rajanna P, Singh Gautam S, Zhang Y, Gan J, Shipkova PA, Marathe P, Humphreys WG (2019) Evidence for the Validity of Pyridoxic Acid (PDA) as a Plasma-Based Endogenous Probe for OAT1 and OAT3 Function in Healthy Subjects. The Journal of pharmacology and experimental therapeutics 368(1):136–145

[89] Vree TB, Van Ewijk-Beneken Kolmer EW, Wuis EW, Hekster YA, Broekman MM (1993) Interindividual variation in the capacity-limited renal glucuronidation of probenecid by humans. Pharmacy world & science : PWS 15(5):197–202

[90] Vree TB, Van Ewijk-Beneken Kolmer EW, Wuis EW, Hekster YA (1992) Capacity-limited renal glucuronidation of probenecid by humans. A pilot Vmax-finding study. Pharmaceutisch weekblad Scientific edition 14(5):325–31

[91] Ito Y, Fukami T, Yokoi T, Nakajima M (2014) An orphan esterase ABHD10 modulates probenecid acyl glucuronidation in human liver. Drug metabolism and disposition: the biological fate of chemicals 42(12):2109–16

[92] Emanuelsson BM, Beermann B, Paalzow LK (1987) Non-linear elimination and protein binding of probenecid. European journal of clinical pharmacology 32(4):395–401

[93] Selen A, Amidon GL, Welling PG (1982) Pharmacokinetics of probenecid following oral doses to human volunteers. Journal of pharmaceutical sciences 71(11):1238–42

[94] Landersdorfer CB, Kirkpatrick CMJ, Kinzig M, Bulitta JB, Holzgrabe U, Drusano GL, Sörgel F (2009) Competitive inhibition of renal tubular secretion of gemifloxacin by probenecid. Antimicrobial agents and chemotherapy 53(9):3902–7

[95] Meyer M, Schneckener S, Ludewig B, Kuepfer L, Lippert J (2012) Using expression data for quantification of active processes in physiologically based pharmacokinetic modeling. Drug metabolism and disposition: the biological fate of chemicals 40(5):892–901

[96] Scotcher D, Billington S, Brown J, Jones CR, Brown CDA, Rostami-Hodjegan A, Galetin A (2017) Microsomal and cytosolic scaling factors in dog and human kidney cortex and application for in vitro-in vivo extrapolation of renal metabolic clearance. Drug Metabolism and Disposition 45(5):556–568

[97] Prasad B, Evers R, Gupta A, Hop CECA, Salphati L, Shukla S, Ambudkar SV, Unadkat JD (2014) Interindividual variability in hepatic organic anion-transporting polypeptides and P-Glycoprotein (ABCB1) protein expression: Quantification by liquid chromatography tandem mass spectroscopy and influence of genotype, age, and sex. Drug Metabolism and Disposition 42(1):78–88

[98] Nishimura M, Naito S (2006) Tissue-specific mRNA expression profiles of human phase I metabolizing enzymes except for cytochrome P450 and phase II metabolizing enzymes. Drug metabolism and pharmacokinetics 21(5):357–74

[99] Rodrigues AD (1999) Integrated cytochrome P450 reaction phenotyping: attempting to bridge the gap between cDNA-expressed cytochromes P450 and native human liver microsomes. Biochemical pharmacology 57(5):465–480

[100] Nishimura M, Yaguti H, Yoshitsugu H, Naito S, Satoh T (2003) Tissue distribution of mRNA expression of human cytochrome P450 isoforms assessed by high-sensitivity real-time reverse transcription PCR. Yakugaku Zasshi 123(5):369–375

[101] Margaillan G, Rouleau M, Fallon JK, Caron P, Villeneuve L, Turcotte V, Smith PC, Joy MS, Guillemette C (2015) Quantitative profiling of human renal UDP-glucuronosyltransferases and glucuronidation activity: a comparison of normal and tumoral kidney tissues. Drug metabolism and disposition: the biological fate of chemicals 43(4):611–9

[102] National Center for Biotechnology Information (NCBI) (2019) Expressed Sequence Tags (EST) from UniGene

[103] Prasad B, Lai Y, Lin Y, Unadkat JD (2013) Interindividual variability in the hepatic expression of the human breast cancer resistance protein (BCRP/ABCG2): effect of age, sex, and genotype. Journal of pharmaceutical sciences 102(3):787–93

[104] Kolesnikov N, Hastings E, Keays M, Melnichuk O, Tang YA, Williams E, Dylag M, Kurbatova N, Brandizi M, Burdett T, Megy K, Pilicheva E, Rustici G, Tikhonov A, Parkinson H, Petryszak R, Sarkans U, Brazma A (2015) ArrayExpress update—simplifying data submissions. Nucleic Acids Research 43(D1):D1113–D1116

[105] Deo AK, Prasad B, Balogh L, Lai Y, Unadkat JD (2012) Interindividual variability in hepatic expression of the multidrug resistance-associated protein 2 (MRP2/ABCC2): quantification by liquid chromatography/tandem mass spectrometry. Drug metabolism and disposition: the biological fate of chemicals 40(5):852–5

[106] Prasad B, Johnson K, Billington S, Lee C, Chung GW, Brown CDA, Kelly EJ, Himmelfarb J, Unadkat JD (2016) Abundance of drug transporters in the human kidney cortex as quantified by quantitative targeted proteomics. Drug Metabolism and Disposition 44(12):1920–1924

[107] Nishimura M, Naito S (2005) Tissue-specific mRNA expression profiles of human ATP-binding cassette and solute carrier transporter superfamilies. Drug Metabolism and Pharmacokinetics 20(6):452–477
